# Dynamic and Static Nature of XH-∗-π and YX-∗-π (X = F, Cl, Br, and I; Y = X and F) in the Distorted π-System of Corannulene Elucidated with QTAIM Dual Functional Analysis

**DOI:** 10.3390/molecules28104219

**Published:** 2023-05-21

**Authors:** Satoko Hayashi, Takahiro Kato, Yuji Sugibayashi, Waro Nakanishi

**Affiliations:** Faculty of Systems Engineering, Wakayama University, 930 Sakaedani, Wakayama 640-8510, Japan; s226068@wakayama-u.ac.jp (T.K.); sugibayashiy@qualtec.co.jp (Y.S.); nakanisi@sys.wakayama-u.ac.jp (W.N.)

**Keywords:** ab initio calculations, quantum theory of atoms-in-molecules (QTAIM), corannulene, hydrogen halides, halogens

## Abstract

The dynamic and static nature of the XH-∗-π and YX-∗-π (X = F, Cl, Br, and I; Y = X and F) interactions in the distorted π-system of corannulene (π(C_20_H_10_)) is elucidated with a QTAIM dual functional analysis (QTAIM-DFA), where asterisks emphasize the presence of bond critical points (BCPs) on the interactions. The static and dynamic nature originates from the data of the fully optimized and perturbed structures, respectively, in QTAIM-DFA. On the convex side, H in F–H-∗-π(C_20_H_10_) and each X in Y–X-∗-π(C_20_H_10_) join to C of the central five-membered ring in π(C_20_H_10_) through a bond path (BP), while each H in X–H-∗-π(C_20_H_10_) does so to the midpoint of C=C in the central five-membered ring for X = Cl, Br, or I. On the concave side, each X in F–X-∗-π(C_20_H_10_) also joins to C of the central five-membered ring with a BP for X = H, Cl, Br, and I; however, the interactions in other adducts are more complex than those on the convex side. Both H and X in X–H-∗-π(C_20_H_10_) (X = Cl and Br) and both Fs in F–F-∗-π(C_20_H_10_) connect to the three C atoms in each central five-membered ring (with three BPs). Two, three, and five BPs were detected for the Cl–Cl, I–H, Br–Br, and I–I adducts, where some BPs do not stay on the central five-membered ring in π(C_20_H_10_). The interactions are predicted to have a vdW to CT-MC nature. The interactions on the concave side seem weaker than those on the convex side for X–H-∗-π(C_20_H_10_), whereas the inverse trend is observed for Y–X-∗-π(C_20_H_10_) as a whole. The nature of the interactions in the π(C_20_H_10_) adducts of the convex and concave sides is examined in more detail, employing the adducts with X–H and F–X placed on their molecular axis together with the π(C_24_H_12_) and π(C_6_H_6_) adducts.

## 1. Introduction

Hydrogen bonds (HBs) [1,2,3,4,5,6,7,8,9,10,11,12] and halogen bonds (XBs) [13,14,15,16] are fundamentally important because of their molecular association ability due to the stabilization of the energy system. HBs and XBs are applied in a wide variety of fields in the chemical and biological sciences [17,18,19] such as crystal engineering, supramolecular soft matter, and nanoparticles. The nature of HBs and XBs has also been discussed based on the theoretical background with the structural aspects [15,20] containing the σ-hole developed on the halogen atoms in XBs. The XH-∗-π and YX-∗-π adducts also form when π-orbitals interact with hydrogen halides and halogen or interhalogen molecules. They are referred to as π-HBs and π-XBs, respectively. In this case, the electrophilic σ*-orbitals of the molecules interact attractively with the π-orbitals, similarly to the case of the n-orbitals on the heteroatoms.

We recently reported the dynamic and static nature of π-HBs and π-XBs in the coronene π-system (π(C_24_H_12_)) [21,22], together with those in benzene (π(C_6_H_6_)) [23,24,25], naphthalene (π(C_10_H_8_)) [26], and anthracene (π(C_14_H_10_)) [27]. We have also been very interested in the behavior of π-HBs and π-XBs in distorted π-systems. π-Electron systems, such as π(C_24_H_12_), seem moderately rigid and moderately flexible. As a result, distorted π-systems such as corannulene (C_20_H_10_) [28,29,30,31] and sumanene (C_21_H_12_) [32,33] will form, where π(C_20_H_10_) and π(C_21_H_12_) are found in fullerenes as partial structures. It must be of particular interest to clarify the differences in the reactivity between the planar and distorted π-systems. The π-systems of coronene (π(C_24_H_12_)) and corannulene (π(C_20_H_10_)) must be the attractive candidates for the purpose. In the case of the bowl-shaped π(C_20_H_10_), the π-orbitals are extended to the convex (cv) side, while they will shrink to the concave (cc) side. This electronic structure will play an important role in the formation of the adducts between corannulene and XH and XY. These clarifications will allow us to anticipate the interactions of bowl-shaped aromatic ring compounds.

**Scheme 1:**
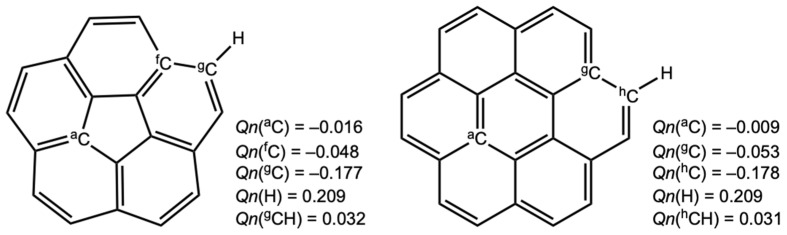
Natural charges (*Qn*) on C and H atoms in corannulene (left) and coronene (right) evaluated with the NPA under the M06-2X/BSS-A//MP2/BSS-A.

The main characteristics of the π-system in corannulene, together with the differences from that of coronene, are explained as follows. Both corannulene and coronene are the neutral aromatic hydrocarbons of the condensed benzene structures, where the central rings of corannulene and coronene contain five and six membered rings of the π-systems, respectively. The π-system of the five membered ring will be more negative, relative to the case of that of the six membered ring, since both π-systems tend to be stabilized by the formation of the 6π electron system. This factor can be examined based on the charge distributions in corannulene and coronene calculated based on the natural population analysis (NPA) [34]. Scheme 1 showed the charge (*Qn*) evaluated by NPA. The ^a^C atom of the central five-membered ring in corannulene (*Qn*(^a^C) = –0.016) is more negatively charged than that of coronene (*Qn*(^a^C) = –0.009) (*cf*.: *Qn*(^f^C) = –0.048 in corannulene and *Qn*(^g^C) = –0.050 in coronene). On the other hand, *Qn*(C–H) (= 0.032) of the outside ring in corannulene is charged positively slightly more than that in coronene (*Qn*(C–H) = 0.031), where *Qn*(C–H) = 0.000 for benzene.

**Figure 1:**
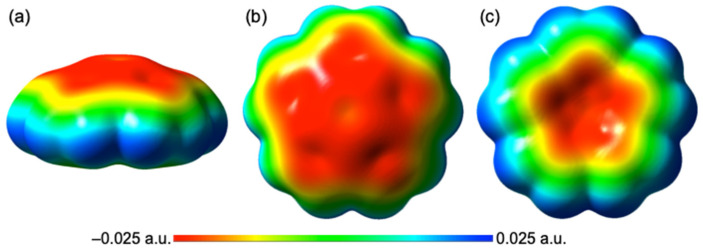
EPS for corannulene evaluated with MP2/BSS-A. From the (**a**) lateral side, (**b**) cv side, and (**c**) cc side.

**Scheme 2:**
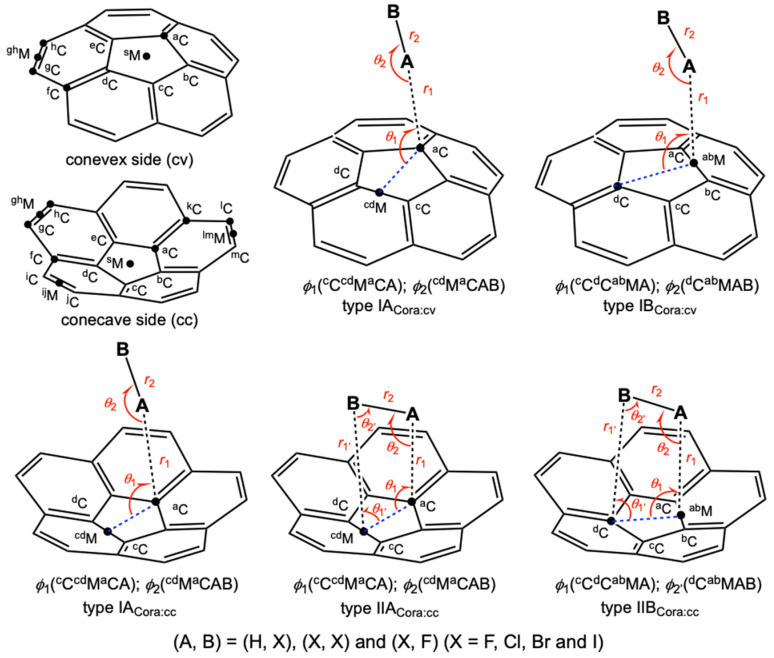
Convex (cv) and concave (cc) sides of corannulene and definition of structural types of B–A⋯π(C_20_H_10_), to be clarified, where (A, B) = (H, X), (X, X), or (X, F) (X = F, Cl, Br, and I) with structural parameters.

In the bowl-shaped π-system of C_20_H_10_, the π-orbitals are extended to the cv side, while they will shrink to the cc side. Namely, the electron density *ρ*(***r***) must be more widely extended on the cv side relative to the case of the cc side, as expected. The expectation can be visualized by the electron potential surface (EPS) on the cv and cc sides of corannulene. Figure 1 shows EPS on the cv and cc sides of corannulene, together with the lateral view. The electron–electron repulsive factor between π(C_20_H_10_) and B–A in B–A-∗-π(C_20_H_10_) (B–A = X–H, X–X, and F–X) will also play an important role in the interaction distances. The interaction distances are named *r*_1_ (see Scheme 2). Such a repulsion could be larger on the cc side than that on the cv side in the bowl-shaped π(C_20_H_10_), although *r*_1_ decreases as the CT interaction of the π(C_20_H_10_)→σ*(X–Y: X = X or F) type increases. The A and/or B dependence in *r*_1_ is of interest.

The nature of the XH-∗-π in XH-∗-π(C_20_H_10_) and YX-∗-π in YX-∗-π(C_20_H_10_) will be elucidated, keeping in mind the above viewpoints, comparing that of the nature in the π(C_24_H_12_) (and π(C_6_H_6_)), after the optimizations of the adducts.

Scheme 2 illustrates the structures of corannulene adducts B–A⋯π(C_20_H_10_) (B–A = Y–X: X = F, Cl, Br, and I; Y = H, X, and F) to be clarified. Scheme 2 defines the types of structures together with the structural parameters. The optimized structures of B–A⋯π(C_20_H_10_) are referred to as type I_Cora_ if B–A interacts with the corannulene π-system through only one site of B–A. In this case, B–A is aligned close to the molecular axis of corannulene. Type I_Cora_ are referred to as type IA_Cora_ and type IB_Cora_, respectively, if B–A interacts with a carbon atom or a midpoint of a C=C bond. The bowl-shaped π-system of π(C_20_H_10_) extends more widely over the outside area of cv but more narrowly to the inside area of cc. The structures of the adducts are controlled by the different electronic structures of the two sides. Type I_Cora_ are referred to as type I_Cora:cv_ and I_Cora:cc_, respectively, if B–A interacts with π(C_20_H_10_) on the cv and cc sides, respectively. The structure is type IIB_Cora_ when B–A seems to interact with π(C_20_H_10_) through both sides of B–A, which is observed on the cc side.

**Scheme 3:**
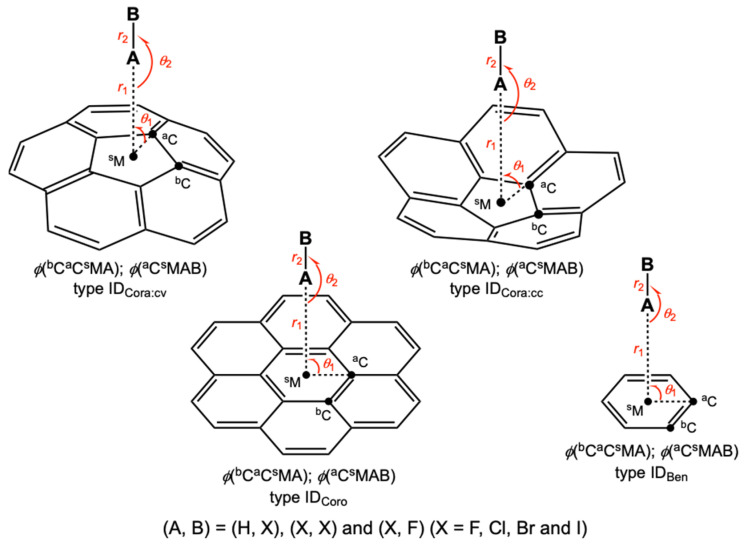
Type ID structures of A–B⋯π and the definitions of the structural parameters for π of π(C_20_H_10_) on the convex (cv) and concave (cc) sides, π(C_24_H_12_), and π(C_6_H_6_), where (A, B) = (H, X), (X, X), or (X, F) (X = F, Cl, Br, and I).

Scheme 3 shows the structures of B–A⋯π (B–A = Y–X: X = F, Cl, Br, and I; Y = H, X, and F) for π of π(C_20_H_10_) (both the cv and cc sides), π(C_24_H_12_), and π(C_6_H_6_). The structures are limited to those for B–A in B–A⋯π being placed on the molecular axis. The structures are referred to as the ID type. The differences and similarities in the nature of the interactions between the planar and distorted π-systems together with the cv and cc sides are examined employing the ID type, shown in Scheme 3. The ID-type structure enables comparison of the nature under the same conditions, where the optimized structures are very different for the adducts with the three π-systems.

The nature of the interactions is analyzed employing the quantum theory of atoms-in-molecules dual functional analysis (QTAIM-DFA) [35,36,37], which we proposed, after the QTAIM approach introduced by Bader [38,39]. A bond critical point (BCP, ∗) is an important concept in QTAIM, which appears on each bond path (PB). QTAIM functions for the interactions in question are calculated at the BCPs. *ρ*(***r***) at BCP is denoted by *ρ*_b_(***r***_c_), as are other QTAIM functions, such as the total electron energy densities *H*_b_(***r***_c_), potential energy densities *V*_b_(***r***_c_), and kinetic energy densities *G*_b_(***r***_c_). A chemical bond or interaction between A and B is denoted by A–B, which corresponds to a BP in QTAIM. We use A-∗-B for BP, where the asterisk emphasizes the existence of a BCP in A–B [35,36].

Equations (1) and (2) show the relationships among the functions (*cf*.: Virial theorem for Equation (2)) [38,39].

*H*_b_(***r***_c_) = *G*_b_(***r***_c_) + *V*_b_(***r***_c_) (1)

(*ћ*^2^/8*m*)∇^2^*ρ*_b_(***r***_c_) = *H*_b_(***r***_c_) − *V*_b_(***r***_c_)/2 = *G*_b_(***r***_c_) + *V*_b_(***r***_c_)/2 (2)

*H*_b_(***r***_c_) is plotted versus *H*_b_(***r***_c_) − *V*_b_(***r***_c_)/2 in QTAIM-DFA. Data from the perturbed structures around fully optimized structures are employed for the plots, in addition to the fully optimized ones [35,36,37]. The perturbed structures in this work are generated by using the coordinates derived from the compliance constants of the internal vibrations *C_ii_* [40,41,42,43,44]. The method is named CIV. CIV is recognized to be the most reliable method to generate the perturbed structures; however, it cannot be applied when BP starts at least one BCP. The perturbed structures are also generated by using the normal coordinates of the (best-fitted) internal vibrations [45,46]. The method is named NIV, which is also reliable. However, we must be careful when the (best-fitted) internal vibrations are not located in the interactions in question. Results with CIV and/or NIV are discussed in the text, selecting the more approximating one if there are some differences.

Data from the fully optimized structures are analyzed using the polar coordinate (*R*, *θ*) representation, which corresponds to the static nature of the interactions [35,36,37,40]. Each interaction plot, for the data from both the perturbed and the fully optimized structures, is expressed by (*θ*_p_, *κ*_p_). While *θ*_p_ corresponds to the tangent line of the plot, *κ*_p_ is the curvature. *θ* and *θ*_p_ are measured from the *y*-axis and the *y*-direction, respectively. We proposed the concept of the “dynamic nature of interactions” based on (*θ*_p_, *κ*_p_) [35,36,37,40]. We named (*R*, *θ*) and (*θ*_p_, *κ*_p_) the QTAIM-DFA parameters. (See also footnotes of Table 1 for the definition). QTAIM-DFA is applied to typical chemical bonds, and interactions and rough criteria are established. QTAIM-DFA and the criteria are explained in the Supplementary Materials using Schemes S1–S3, Figures S1 and S2, Table S1, and Equations (S1)–(S7). The basic concept of the QTAIM approach is also explained.

The analyzed results of QTAIM-DFA will help to elucidate the nature of the interactions and a better understanding of the adducts formed by the interactions. QTAIM-DFA must be one of the best methodologies to elucidate the nature of the interactions. QTAIM-DFA will not only classify the interactions but also elucidate the nature. With the results, we will be able to access to the nature of the interactions in the adduct between corannulene and XH, XX, and FX (X = F, Cl, Br, and I) by elucidating the nature of the interactions with the method.

The dynamic and static nature of π-HBs and π-XBs in the bowl-shaped corannulene π-system (π(C_20_H_10_)) is elucidated with QTAIM-DFA after the clarification of the structural feature. Herein, we present the results of the investigations on the nature of X–H-∗-π(C_20_H_10_), X–X-∗-π(C_20_H_10_), and F–X-∗-π(C_20_H_10_) (X = F, Cl, Br, and I), where the nature is classified and characterized by employing the criteria as a reference. The differences and similarities in the nature of the interactions in B–A-∗-π for π of π(C_20_H_10_) (both cv and cc sides), π(C_24_H_12_) [21], and π(C_6_H_6_) [23,24,25] are also discussed.

## 2. Methodological Details of the Calculations

Calculations were performed by employing the Gaussian 09 program package [47]. The 6-311G(2d,p) basis sets for C and H were employed for the calculations, with the basis sets of the 6-311 + G(3df) for F, Cl, and Br, and the (7433211/743111/7411/2 + 1s1p) type for I, implemented from the Sapporo Basis Set Factory [48]. The basis set system is named basis set system-A (BSS-A). The Møller–Plesset second-order energy correlation (MP2) level [49,50,51] was applied to BSS-A (MP2/BSS-A). Optimized structures were confirmed by a frequency analysis. QTAIM functions were calculated using the AIM2000 [52,53] and AIMAll [54] programs with the same method as the optimizations. The optimized structures were not corrected with the BSSE method. A natural bond orbital analysis (NBO) and natural population analysis (NPA) were calculated with M06-2X/BSS-A//MP2/BSS-A [34,55].

Equation (3) explains the process to generate the perturbed structures with CIV [40,41,42,43,44]. The coordinates derived from the *C_ii_* values (**C***_i_*) are used to generate the *i*-th perturbed structures in question (**S***_iw_*). **C***_i_* is added to the standard orientation of the fully optimized structure (**S**_o_) in the matrix representation. The coefficient *g_iw_* in Equation (3) controls the difference in the structures between **S***_iw_* and **S**_o_: *g_iw_* are determined to satisfy Equation (4) for an interaction in question, where *r* and *r*_o_ show the interaction distances in question in the perturbed and fully optimized structures, respectively, with *a*_o_ of the Bohr radius (0.52918 Å). In the case of NIV, the process can be similarly explained by replacing **C***_i_* in Equation (3) to the (best-fitted) normal coordinates of the *i*-th internal vibration (**N***_i_*), which is formulated by **S***_iw_* = **S**_o_ + *g_iw_*· **N***_i_*. The **C***_i_* and **N***_i_* values of five digits are used for the generation.

**S***_iw_* = **S**_o_ + *g_iw_*· **C***_i_* (3)

*r* = *r*_o_ + *wa*_o_ (*w* = (0), ±0.05, and ±0.1; *a*_o_ = 0.52918 Å) (4)

*y* = *c*_o_ + *c*_1_*x* + *c*_2_*x*^2^ + *c*_3_*x*^3^ (*R*_c_^2^: square of the correlation coefficient) (5)

In the QTAIM-DFA treatment, *H*_b_(***r***_c_) is plotted versus *H*_b_(***r***_c_) − *V*_b_(***r***_c_)/2 for data of five points of *w* = 0, ±0.05, and ±0.1 in Equation (4) unless otherwise noted. Each plot is analyzed using a regression curve of the cubic function as shown in Equation (5), where (*x*, *y*) = (*H*_b_(***r***_c_) − *V*_b_(***r***_c_)/2, *H*_b_(***r***_c_)) (*R_c_*^2^ (square of correlation coefficient) > 0.99999 in usual) [37].

## 3. Results and Discussion

### 3.1. Optimizations of B–A⋯π(C_20_H_10_) (B–A = X–H, X–X, and F–X)

**Figure 2:**
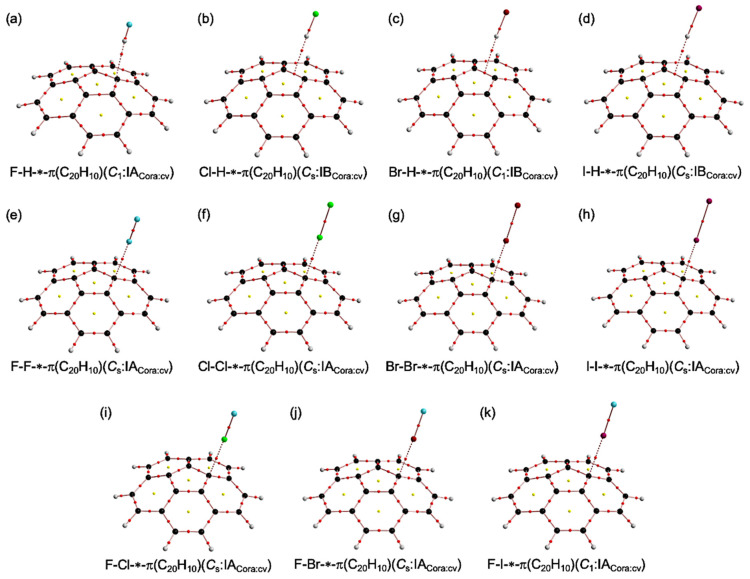
Molecular graphs for X–H-∗-π(C_20_H_10_), X–X-∗-π(C_20_H_10_), and F–X-∗-π(C_20_H_10_) on the convex (cv) side, evaluated with MP2/BSS-A: F–H-∗-π(C_20_H_10_) (*C*_1_: type IA_Cora:cv_) (**a**), Cl–H-∗-π(C_20_H_10_) (*C*_s_: type IB_Cora:cv_) (**b**), Br–H-∗-π(C_20_H_10_) (*C*_1_: type IB_Cora:cv_) (**c**), I–H-∗-π(C_20_H_10_) (*C*_s_: type IB_Cora:cv_) (**d**), F–F-∗-π(C_20_H_10_) (*C*_s_: type IB_Cora:cv_) (**e**), Cl–Cl-∗-π(C_20_H_10_) (*C*_s_: type IA_Cora:cv_) (**f**), Br–Br-∗-π(C_20_H_10_) (*C*_s_: type IA_Cora:cv_) (**g**), I–I-∗-π(C_20_H_10_) (*C*_s_: type IA_Cora:cv_) (**h**), F–Cl-∗-π(C_20_H_10_) (*C*_s_: type IA_Cora:cv_) (**i**), F–Br-∗-π(C_20_H_10_) (*C*_s_: type IA_Cora:cv_) (**j**), and F–I-∗-π(C_20_H_10_) (*C*_1_: type IA_Cora:cv_) (**k**). BPs are drawn as pink lines, BCPs as red dots, and CCPs (cage critical points) as green dots. Carbon atoms are indicated in black and hydrogen atoms are in grey, with fluorine, chlorine, bromine, and iodine atoms in dark yellow, green, dark brown, and dark purple, respectively.

**Figure 3:**
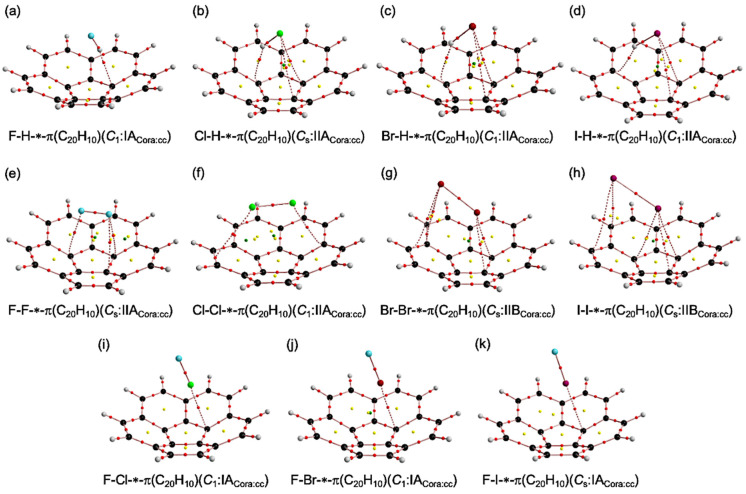
Molecular graphs for X–H-∗-π(C_20_H_10_), X–X-∗-π(C_20_H_10_), and F–X-∗-π(C_20_H_10_) on the concave (cc) side, evaluated with BSS-A: F–H-∗-π(C_20_H_10_) (*C*_1_: type IA_Cora:cc_) (**a**), Cl–H-∗-π(C_20_H_10_) (*C*_s_: type IIA_Cora:cc_) (**b**), Br–H-∗-π(C_20_H_10_) (*C*_1_: type IIA_Cora:cc_) (**c**), I–H-∗-π(C_20_H_10_) (*C*_1_: type IIA_Cora:cc_) (**d**), F–F-∗-π(C_20_H_10_) (*C*_s_: type IIA_Cora:cc_) (**e**), Cl–Cl-∗-π(C_20_H_10_) (*C*_1_: type IIA_Cora:cc_) (**f**), Br–Br-∗-π(C_20_H_10_) (*C*_s_: type IIB_Cora:cc_) (**g**), I–I-∗-π(C_20_H_10_) (*C*_s_: type IIB_Cora:cc_) (**h**), F–Cl-∗-π(C_20_H_10_) (*C*_1_: type IA_Cora:cc_) (**i**), F–Br-∗-π(C_20_H_10_) (*C*_1_: type IA_Cora:cc_) (**j**), and F–I-∗-π(C_20_H_10_) (*C*_s_: type IA_Cora:cc_) (**k**). BPs are drawn as pink lines, BCPs as red dots, RCPs (ring critical points) as yellow dots, and CCPs (cage critical points) as green dots. Carbon atoms are indicated in black and hydrogen atoms are in grey, with fluorine, chlorine, bromine, and iodine atoms in dark yellow, green, dark brown, and dark purple, respectively.

The optimizations of B–A⋯π(C_20_H_10_), where B–A = X–H, X–X, and F–X (X = F, Cl, Br, and I), were started with MP2/BSS-A, putting B–A on various places close to the symmetry axis (^s^M), some carbon atoms (^a^C, ^f^C, and/or ^g^C) and/or the midpoint of the outside C=C bond (^gh^M) on the cv and cc sides (see Scheme 2 and Scheme 3 for the definitions). On the cv side, all optimizations of B–A⋯π(C_20_H_10_) converged to type IA_Cora:cv_, except for type IB_Cora:cv_ of X–H⋯π(C_20_H_10_) (X = Cl, Br, and I). For the cc side, they converged to type IA_Cora:cc_ for F–H⋯π(C_20_H_10_) and F–X⋯π(C_20_H_10_) (X = Cl, Br, and I), type IIA_Cora:cc_ for X–H⋯π(C_20_H_10_) (X = Cl, Br, and I) and X–X⋯π(C_20_H_10_) (X = F and Cl), and type IIB_Cora:cc_ for X–X⋯π(C_20_H_10_) (X = Br and I). The optimized *C*_1_ structures were further optimized assuming the *C*_s_ structures when the *C*_1_ structures were very close to the *C*_s_ symmetry. The structural parameters are summarized in Table S2 of the Supplementary Materials. The optimized structures are not shown in the figures, but they can be found as molecular graphs (see Figure 2 and Figure 3). They are drawn on the optimized structures.

Next, molecular graphs are examined after clarification of the structural feature of B–A⋯π(C_20_H_10_).

### 3.2. Molecular Graphs for B–A-∗-π(C_20_H_10_) (B–A = X–H, X–X, and F–X)

The molecular graphs are drawn on the optimized structures with MP2/BSS-A for X–H-∗-π(C_20_H_10_), X–X-∗-π(C_20_H_10_), and F–X-∗-π(C_20_H_10_), where X = F, Cl, Br, and I. Figure 2 and Figure 3 illustrate the molecular graphs on the cv and cc sides of π(C_20_H_10_), respectively. All BCPs expected are clearly detected, containing those for the XH-∗-π, XX-∗-π, and YX-∗-π interactions in question, together with the additional ones. The BPs in question appear clearly, with BCPs, ring critical points (RCPs), and cage critical points (CCPs), if any. The structural features of the species are well visualized by the molecular graphs.

In the reaction on the convex side of corannulene, only the structure of the monodentate coordination to the central five-membered ring was optimized with HX, XX, and FX. On the other hand, on the concave side, similarly, bidentate to pentadentate structures were optimized for coordination to the central five-membered ring, except for HF and FX. In the adducts of X–H-∗-π(C_20_H_10_), X–X-∗-π(C_20_H_10_), and F–X-∗-π(C_20_H_10_), electrons will flow from the corannulene to the components; thus, the electron density will increase on the end of the components.

As shown in Figure 2 and Figure 3, BPs for B–A-∗-π(C_20_H_10_) (B–A = X–H, X–X, and F–X) seem almost straight at first glance. BPs, as defined in QTAIM, is the connection of the minima of the electron density, where a BCP exists for each BP between two interacting atoms, although BP sometimes starts from a BCP of a bond. The BPs are not necessarily the shortest path. In other words, the lengths of BPs (*r*_BP_) and the straight-line distances (*R*_SL_) are approximately equal when the interaction characteristics are relatively simple, but they differ greatly when they are not. Such things often happen in the case of weak interactions. To further examine the behavior of the BPs, *r*_BP_ and *R*_SL_ were calculated. The values are collected in Table S4 of the Supplementary Materials, together with the differences between them Δ*r*_BP_ (=*r*_BP_ − *R*_SL_). The Δ*r*_BP_ values are less than 0.10 Å for all BPs in question, except for Br–H-∗-π(C_20_H_10_) (*C*_1_: type IIA_Cora:cc_; 0.180 Å), Br–H-∗-π(C_20_H_10_) (*C*_1_: type IB_Cora:cv_; 0.681 Å), and Cl–Cl-∗-π(C_20_H_10_) (*C*_1_: type IIA_Cora:cc_; Δ*r*_BP_ = 0.701 Å). The *r*_BP_ is plotted versus *R*_SL_, which is shown in Figure S4 of the Supplementary Materials. The plot gave a very good correlation (*y* = 0.995*x* + 0.035: *R*_c_^2^ = 0.998), if omitted Br–H-∗-π(C_20_H_10_) (*C*_1_: type IIA_Cora:cc_), Br–H-∗-π(C_20_H_10_) (*C*_1_: type IB_Cora:cv_), and Cl–Cl-∗-π(C_20_H_10_) (*C*_1_: type IIA_Cora:cc_). Consequently, all BPs in question can be approximated as straight lines except for the three cases. There must exist some reasons for the large Δ*r*_BP_ values. Typical cases where large Δ*r*_BP_ values are observed are shown below. BPs often curve in the area (very) close to atoms. A BP appears when a maximum line of *ρ*(***r***) connects two atoms. In this case, the maximum line does not often direct toward the second atom just after it starts the first atom. Such a case is also observed in which a BP directs to a BCP of another bond, where it reaches not the BCP but an atom corresponding to the BCP.

### 3.3. Survey of B–A-∗-π(C_20_H_10_) (B–A = X–H, X–X, and F–X)

The energies for the formation of the adducts from the components, Δ*E* [= *E*(B–A∙∙∙π(C_20_H_10_))–(*E*(B–A) + *E*(C_20_H_10_)): B–A = X–H, X–X, and F–X], were calculated with MP2/BSS-A. The Δ*E*_ES_ and Δ*E*_ZP_ values are collected in Table S3 of the Supplementary Materials, where Δ*E*_ES_ and Δ*E*_ZP_ stand for Δ*E* on the energy surface and those with the collections by the zero-point energy, respectively, together with the second-perturbation energies corresponding to the donor-acceptor interaction from π(C=C) to σ*(X–H) or σ*(Y–X), calculated with M06-2X/BSS-A//MP2/BSS-A.

To confirm the validity of the argument using energy surfaces, we checked the correlation between Δ*E*_ZP_ and Δ*E*_ES_. Δ*E*_ZP_ is plotted versus Δ*E*_ES_, which is shown in Figure S3 of the Supplementary Materials. The plots gave (very) good correlations: *y* = 1.015*x* + 2.67: *R*_c_^2^ = 0.996 (*n* (number of data points) = 11) for cv, *y* = 1.025*x* + 3.08: *R*_c_^2^ = 0.999 (*n* = 11) for cc, and *y* = 1.021*x* + 2.90: *R*_c_^2^ = 0.998 (*n* = 22) for all. Therefore, Δ*E*_ES_ can be used for the discussion of Δ*E*.

**Figure 4:**
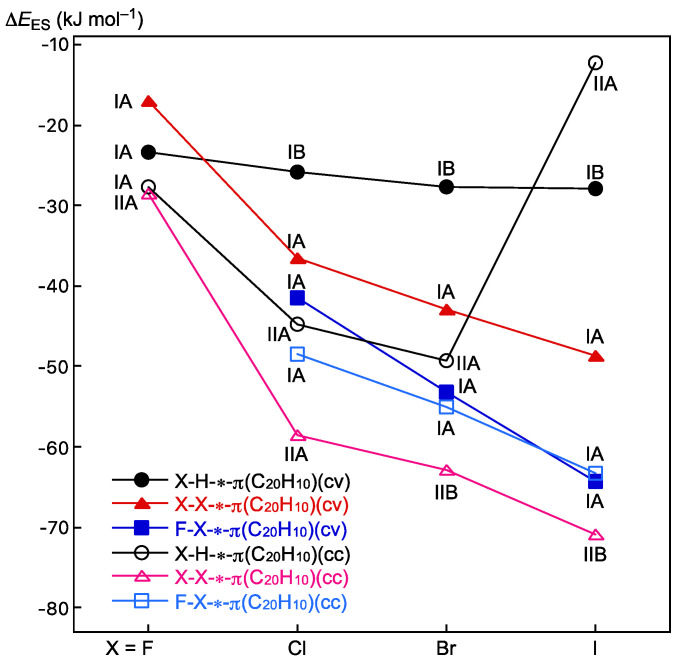
Plots of Δ*E*_ES_ of B–A-∗-π(C_20_H_10_) (B–A = X–H, X–X, and F–X) on the cv and cc sides versus halogens (X) calculated with M06-2X/BSS-A//MP2/BSS-A.

Figure 4 shows the plots of Δ*E*_ES_ of B–A-∗-π(C_20_H_10_) (B–A = X–H, X–X, and F–X) on the cv and cc sides versus halogens (X) calculated with M06-2X/BSS-A//MP2/BSS-A. The energies for the formation of the adducts from the components were more stabilized on the concave side except for I–H-∗-π(^f^C) (IIA_Cora:cc_) and F–I-∗-π(^a^C) (IA_Cora:cc_) and become more stable as the atomic number of the halogen increases. I–H-∗-π(^f^C) (IIA_Cora:cc_) is out of trend because the H interacts with the outer carbon ^f^C (Figure 2d). The stabilization energy is almost the same for F–I-∗-π(^a^C) (IA_Cora:cc_) and F–I-∗-π(^a^C) (IA_Cora:cv_). X–X-∗-π was also more stabilized than X–H-∗-π, and X–Y-∗-π was more stabilized than X–X-∗-π except for I–H-∗-π(^f^C) (*C*_1_: IIA_Cora:cc_).

**Figure 5:**
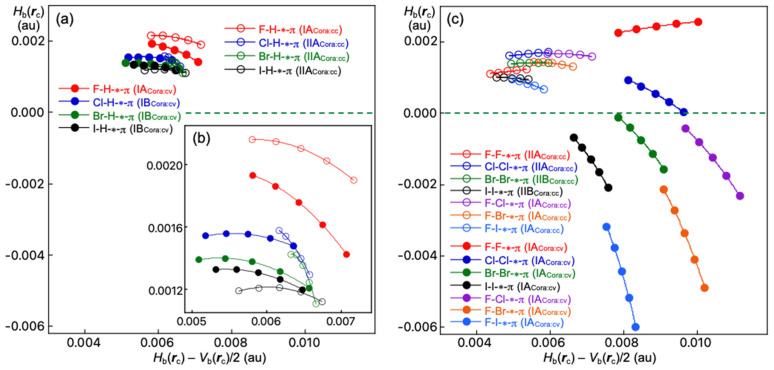
QTAIM-DFA plots of *H*_b_(***r***_c_) versus *H*_b_(***r***_c_)–*V*_b_(***r***_c_)/2 for X–H-∗-π(C_20_H_10_) (**a**), the partial one (**b**), and Y–X-∗-π(C_20_H_10_) (**c**), calculated with MP2/BSS-A. Keys are shown in the figure, in which solid and hollow marks correspond to the cv and cc sides of corannulene, respectively, in X–H-∗-π(C_20_H_10_) and Y–X-∗-π(C_20_H_10_).

QTAIM functions were calculated for B–A-∗-π(C_20_H_10_) (B–A = X–H, X–X, and F–X). Table 1 lists the values. Figure 5 shows a plot of *H*_b_(***r***_c_) versus *H*_b_(***r***_c_)–*V*_b_(***r***_c_)/2 for the interactions in question. Data shown in Table 1 are employed for the plots together with those from the perturbed structures generated with CIV and NIV, although the interactions are limited to the main interactions. The nature of the interactions is clarified by analyzing the plots in Figure 5, according to Equations (S1)–(S4) of the Supplementary Materials.

### 3.4. Nature of B–A-∗-π(C_20_H_10_) (B–A = X–H, X–X, and F–X)

Table 1 lists the *ρ*(***r***), *H*_b_(***r***_c_) − *V*_b_(***r***_c_)/2, and *H*_b_(***r***_c_) values of the QTAIM functions. Table 1 collects the QTAIM-DFA parameters of (*R*, *θ*) and (*θ*_p_, *κ*_p_), the analyzed results, compliance constants *C_ii_* for CIV, employed to generate the perturbed structures, and/or the frequencies corresponding to the interval vibrations employed to generate the perturbed structures with NIV. The values for the main interactions are given in Table 1, while those for the additional interactions on the cv and cc side are in Table S8 of the Supplementary Materials, although the definition is tentative. The *θ*_p_, values for the main interactions with NIV, are plotted versus those with CIV. The plot is shown in Figure S7 of the Supplementary Materials. The correlation is very good (*y* = 0.955*x* + 5.0; *R*_c_^2^ = 0.995), if all data is analyzed except for those from F–H-∗-π(C_20_H_10_) (cv), Cl–H-∗-π(C_20_H_10_) (cc), and Br–H-∗-π(C_20_H_10_) (cc). Only one BP connects the components of the adducts on the cv side, which results in the very good correlation. In the case of the cc side, the correlation is also very good, where the data of Cl–H-∗-π(C_20_H_10_) (cc) and Br–H-∗-π(C_20_H_10_) (cc) are neglected. The results come from the very complex interaction style on the cc side, where only one BP connects the components of the adducts for F–H-∗-π(C_20_H_10_) and F–X-∗-π(C_20_H_10_) (X = Cl, Br, and I), whereas the components of the adducts are connected by the multi-BPs for others.

The trends in *θ* and *θ*_p_ are summarized in Equations (6)–(13). The *θ* value of X–H-∗-π(C_20_H_10_) on the cv side seems smaller than the corresponding values on the cc side except for F–H-∗-π(C_20_H_10_). The *θ* value on the cv side becomes larger in the order shown in Equation (6). The order on the cc side shown in Equation (7) seems very similar to that on the cv side. In the case of Y–X-∗-π(C_20_H_10_), the *θ* value on the cv side is larger than the corresponding value on the cc side, except for F–F-∗-π(C_20_H_10_), as shown in Equations (8) and (9). The *θ* value of Y–X-∗-π(C_20_H_10_) on both the cv and cc sides becomes larger in a similar order, as shown in Equations (8) and (9), respectively. The *θ* values on the cc side seem much smaller than the corresponding values on the cv side except for F–F-∗-π(C_20_H_10_). The bend structure of Y–X-∗-π(C_20_H_10_) (*C*_s_: IIA_Cora:cc_) must be responsible for the much smaller *θ* values on the cc side. The *θ*_p_ values for X–H-∗-π(C_20_H_10_) and Y–X-∗-π(C_20_H_10_) show the same trends as those in the *θ* values.

Order in *θ* of X–H-∗-π(C_20_H_10_) on the cv side:

F–H- (*θ* = 74.7°: IA) ≈ Cl–H- (74.9°: IB) < Br–H- (76.7°: IB) < I–H- (77.5°: IB) (6)

Order in *θ* of X–H-∗-π(C_20_H_10_) on the cc side:

F–H- (*θ* = 72.0°: IA) < Cl–H- (76.9°: IIA) < Br–H- (78.2°: IIA) < I–H- (78.9°: IIA) (7)

Order in *θ* of Y–X-∗-π(C_20_H_10_) on the cv side:

F–F- (*θ* = 74.7°: IA) < Cl–Cl- (86.4°: IA) < Br–Br- (95.0°: IA) < F–Cl- (96.9°: IA) < I–I- (100.3°: IA) < F–Br- (109.3°: IA) < F–I- (119.2°: IA) (8)

Order in *θ* of Y–X-∗-π(C_20_H_10_) on the cc side:

Cl–Cl- (*θ* = 72.8°: IIA) < Br–Br- (75.4°: IIB) ≈ F–F- (76.3°: IIA) < F–Cl- (75.6°: IA) < F–Br- (76.9°: IA) < I–I- (78.7°: IIB) < F–I- (81.1°: IA) (9)

Order in *θ*_p_ of X–H-∗-π(C_20_H_10_) with NIV on the cv side:

Cl–H- (*θ*_p_ = 88.5°: IB) < I–H- (95.9°: IB) ≈ Br–H- (96.2°: IB) < F–H- (111.4°: IA) (10)

Order in *θ*_p_ of X–H-∗-π(C_20_H_10_) with NIV on the cc side:

I–H- (*θ*_p_ = 92.2°: IIA) < F–H- (100.2°: IA) < Cl–H- (125.4°: IIA) < Br–H- (137.7°: IIA) (11)

Order in *θ*_p_ of Y–X-∗-π(C_20_H_10_) with NIV on the cv side:

F–F- (*θ*_p_ = 81.8°: IA) < Cl–Cl- (120.6°: IA) < Br–Br- (139.3°: IA) < F–Cl- (141.8°: IA) < I–I- (145.7°: IA) < F–Br- (157.7°: IA) < F–I- (164.1°: IA) (12)

Order in *θ*_p_ of Y–X-∗-π(C_20_H_10_) with NIV on the cc side:

Cl–Cl- (84.5°: IIA) < Br–Br- (88.5°: IIB) < F–F- (*θ*_p_ = 89.3°: IIA) < F–Cl- (93.8°: IA) < I–I- (94.1°: IIB) < F–Br- (97.1°: IA) < F–I- (107.7°: IA) (13)

Order in *θ*_p_ of X–H-∗-π(C_20_H_10_) with CIV on the cc side:

I–H- (*θ*_p_ = 93.5°: IIA) < F–H- (95.9°: IA) < Cl–H- (108.6°: IIA) < Br–H- (117.2°: IIA) (14)

Order in *θ*_p_ of Y–X-∗-π(C_20_H_10_) with CIV on the cv side:

F–F- (*θ*_p_ = 81.9°: IA) < Cl–Cl- (122.2°: IA) < Br–Br- (141.4°: IA) < F–Cl- (143.2°: IA) < I–I- (147.9°: IA) < F–Br- (159.2°: IA) < F–I- (165.0°: IA) (15)

Order in *θ*_p_ of Y–X-∗-π(C_20_H_10_) with CIV on the cc side:

F–F- (*θ*_p_ = 84.6°: IIA) < Br–Br- (86.3°: IIB) < I–I- (91.2°: IIB) < F–Cl- (95.0°: IA) < F–Br- (99.9°: IA) < F–I- (109.2°: IIB) (16)

The *θ*_p_ values with NIV are discussed first, then those with CIV, since data with CIV are lacking in some cases. The *θ*_p_ values of X–H-∗-π(C_20_H_10_) on both the cv and cc sides seem to increase normally except for F–H-∗-π(C_20_H_10_) on the cv side and I–H-∗-π(C_20_H_10_) on the cc side, as shown in Equations (10), (11), and (14), respectively. The values on the cc side seem larger than those on the cv side, as a whole. The bend IIA structures on the cc side versus the IB structures on the cv side for X–H-∗-π(C_20_H_10_) (X = Cl, Br, and I) must be responsible for the trend in *θ*_p_. The differences in the electronic structures around the cv and cc sides also contribute to the results.

The trends in *θ*_p_ with CIV are shown in Equations (12), (13), (15), and (16); the order in *θ*_p_ of Y–X-∗-π(C_20_H_10_) on the cc side seems similar to that on the cv side. However, the magnitudes of *θ*_p_ on the cv side are much larger than those on the cc side except for F–F-∗-π(C_20_H_10_). The bend structures on the cc sides are mainly responsible for the results.

The nature of the H-∗-π and X-∗-π interactions is discussed next. In QTAIM-DFA, the *θ* values classify the interactions in question, while the *θ*_p_ values characterize them. The *θ* values of 45° < *θ* < 180° (*H*_b_(***r***_c_) − *V*_b_(***r***_c_)/2 > 0) correspond to the closed shell (CS) interactions, which are subdivided into the *pure* CS (*p*-CS) interactions of 45° < *θ* < 90° (*H*_b_(***r***_c_) > 0) and the *regular* CS (*r*-CS) interactions of 90° < *θ* < 180° (*H*_b_(***r***_c_) < 0). All interactions in Table 1 are classified by the *p*-CS and *r*-CS interactions since 68.8° < *θ* < 119.2°. In the *p*-CS region, the character of interactions is the vdW type for 45° < *θ*_p_ < 90° and the typical hydrogen bond type with no covalency (*t*-HB_nc_) for 90° < *θ*_p_ < 125°, where *θ*_p_ = 125° is tentatively given, corresponding to *θ* = 90°. The characteristics of the *r*-CS interactions are similarly defined. As a result, the (*θ*, *θ*_p_) values of (75°, **90°**), (**90°**, 125°), (115°, **150°**), and (150°, **180°**) can be considered to be the borderlines between the nature of interactions for vdW/*t*-HB_nc_, *t*-HB_nc_/*t*-HB_wc_, *t*-HB_wc_/CT-MC, and CT-MC/CT-TBP, respectively, where *t*-HB_wc_, CT-MC, and CT-TBP represent *t*-HB with covalency, molecular complex formation through CT, and trigonal bipyramidal (TBP) adduct formation through CT, respectively. The parameters given in bold are superior to those in plain in the classification and characterization of interactions. The (*θ*, *θ*_p_) values in Table 1 are larger than (72°, 89°) and less than (119°, 165°) for all H-∗-π and X-∗-π interactions. Therefore, the interactions will have the nature of *p*-CS/vdW, *p*-CS/*t*-HB_nc_, *r*-CS/*t*-HB_wc_, or *r*-CS/CT-MC.

The nature of the main interactions is discussed first. The *θ*_p_ values calculated with NIV are employed here. As shown in Table 1, the (*θ*, *θ*_p_) values for F–H-∗-π(C_20_H_10_) (*C*_1_: IA_Cora:cv_) are (74.7°, 111.4°); therefore, the interaction is predicted to have the *t*-HB_nc_ nature that appeared in the *p*-CS region, which is denoted by *p*-CS/*t*-HB_nc_. Both the *θ* and *θ*_p_ values are less than 90° for Cl–H-∗-π(C_20_H_10_) (*C*_1_: IB_Cora:cv_), F–F-∗-π(C_20_H_10_) (*C*_s_: IA_Cora:cv_), and X–X-∗-π(C_20_H_10_) (*C*_s_: IIA_Cora:cc_: X = F and Cl; *C*_s_: IIB_Cora:cc_: X = Br). Therefore, the interactions are predicted to have a *p*-CS/vdW nature. The (*θ*, *θ*_p:NIV_) values are (86.4°, 120.6°) for Cl–Cl-∗-π(C_20_H_10_) (*C*_s_: IA_Cora:cv_), (74.7–77.5°, 95.9–111.4°) for X–H-∗-π(C_20_H_10_) (*C*_1_: IA_Cora:cv_: X = F; IB_Cora:cv_: X = Br and I), and (72.0–81.1°, 92.2–137.7°) for X–H-∗-π(C_20_H_10_) (*C*_1_: IA_Cora:cc_: X = F; IIA_Cora:cc_: X = Cl, Br, and I), I–I-∗-π(C_20_H_10_) (*C*_s_: IIB_Cora:cc_), and F–X-∗-π(C_20_H_10_) (*C*_s_: IA_Cora:cc_: X = Cl, Br, and I). Consequently, the interactions are predicted to have a *p*-CS/*t*-HB_nc_ nature. The Cl-∗-π interaction in Cl–Cl-∗-π(C_20_H_10_) (type IA_Cora:cv_) seems close to the borderline area between *p*-CS and *r*-CS since *θ* = 86.4°, which is close to 90°. On the other hand, the (*θ*, *θ*_p_) values are (95.0–100.3°, 139.3–145.7°) for X–X-∗-π(C_20_H_10_) (*C*_s_: IA_Cora:cv_: X = Br and I) and F–Cl-∗-π(C_20_H_10_) (*C*_s_: IA_Cora:cv_). As a result, the interactions are predicted to have a *r*-CS/*t*-HB_wc_ nature. The (*θ*, *θ*_p_) values are (109.3–119.2°, 157.7–164.1°) for F–X-∗-π(C_20_H_10_) (*C*_s_: IA_Cora:cv_: X = Br and I). Therefore, the interactions are predicted to have a *r*-CS/CT-MC nature. Table 1 summarizes the predicted nature.

As shown in Table 1, the predicted nature of the interactions in question with CIV is the same as the corresponding one with NIV, where the differences in *θ*_p_ between those with NIV and CIV are (very) small (0.1° ≤ Δ*θ*_p_ (= *θ*_p:NIV_ – *θ*_p:CIV_) ≤ 4.7°) except for F–H-∗-π(C_20_H_10_) (*C*_1_: IA_Cora:cv_), Cl–H-∗-π(C_20_H_10_) (*C*_s_: IIA_Cora:cc_), and Br–H-∗-π(C_20_H_10_) (C_1_: IIA_Cora:cc_). The Δ*θ*_p_ values for the three adducts are 12.2°, –16.8°, and –20,5°, respectively. The normal coordinates of the (best-fitted) internal vibrations would not be located on the interactions in question, which would be responsible for the large differences. The large differences in *θ*_p_ are fortunately burred in the larger *θ*_p_ ranges of the interactions in F–H-∗-π(C_20_H_10_) (*C*_1_: IA_Cora:cv_), Cl–H-∗-π(C_20_H_10_) (*C*_s_: IIA_Cora:cc_), and Br–H-∗-π(C_20_H_10_) (C_1_: IIA_Cora:cc_). However, the magnitudes of Δ*θ*_p_ for the three adducts seem much larger than those expected. CIV cannot be applied for the interactions in which BPs start from BCP on C=C of π(C_20_H_10_). There must be other factors for the large magnitudes. Further investigations would be necessary for the detailed discussion on the large magnitudes of Δ*θ*_p_. Therefore, we will not discuss the differences further here.

### 3.5. Factors to Control Structures of B–A-∗-π(C_20_H_10_) (B–A = X–H, X–X, and F–X)

The molecular graphs for B–A-∗-π(C_20_H_10_) (B–A = X–H, X–X, and F–X), shown in Figure 2 and Figure 3, seem very different from those for B–A-∗-π(C_24_H_12_) (B–A = X–H, X–X, and F–X) [21], especially the role of the outside ring in the formation of the adducts. What factor controls the observed differences between the coronene and corannulene adducts? The factors were examined first based on the charge distributions in corannulene and coronene calculated with NPA, of which *Qn* values are shown in Scheme 1. In fact, HX and XY are located near the central 5-membered ring when complexed with corannulene (Figure 2 and Figure 3). Additionally, the formation of bidentate to pentadentate coordination complexes of HX and XY with corannulene on the concave side indicates that these interactions are attractive interactions. The calculated values seem consistent with the observed results, although the relation to the reactivity is complex.

**Figure 6:**
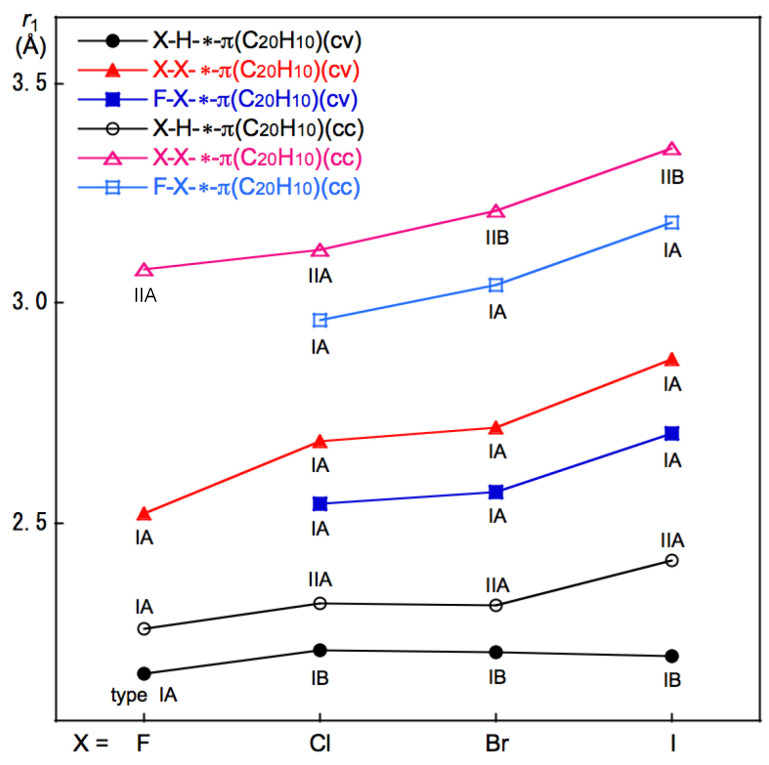
Plots of *r*_1_ of B–A-∗-π(C_20_H_10_) (B–A = X–H, X–X, and F–X) on the cv and cc sides versus halogens (X). See Scheme 2 in text for *r*_1_.

How are the *r*_1_ values of B–A-∗-π(C_20_H_10_) (B–A = X–H, X–X, and F–X)? The *r*_1_ values are plotted versus X = F, Cl, Br, and I, separately by X–H, X–X, and F–X and the cv and cc sides (totally six cases). Figure 6 illustrates the plot, which shows the clear trend in *r*_1_ of B–A-∗-π(C_20_H_10_). The order in *r*_1_ is summarized in Equation (17), where the *r*_1_ values of the same X are compared. Next, the trends in *r*_1_ are individually discussed.

Order in *r*_1_ of B–A-∗-π(C_20_H_10_):

B–A- (cv or cc) = X–H- (cv) < X–H- (cc) < F–X- (cv) < X–X- (cv) < F–X- (cc) < X–X- (cc)(17)

The *r*_1_ values on the cc side (*r*_1_(cc)) are (very) close to *r*_1_(cv) (*r*_1_(cv) ≈ *r*_1_(cc)) for X–H-∗-π(C_20_H_10_) if *r*_1_ of the same X are compared. On the other hand, *r*_1_(cc) is (much) larger than *r*_1_(cv) (*r*_1_(cv) ≪ *r*_1_(cc)) for X–X-∗-π(C_20_H_10_) and F–X-∗-π(C_20_H_10_) (X = F, Cl, Br, and/or I). The structures of X–X-∗-π(C_20_H_10_) (X = Cl, Br, and I) are the IIB_Cora:cc_ type on the cc side, whereas they are the IB_Cora:cc_ type on the cv side. The difference in the structures must be the reason for *r*_1_ (cv) ≪ *r*_1_ (cc) in X–X-∗-π(C_20_H_10_). In the case of F–X-∗-π(C_20_H_10_) (X = Cl, Br, and I), the structures are the IA_Cora_ type on both the cc and cv sides. Therefore, the difference in the steric repulsion between the cc and cv sides is the reason for the calculated results. The steric repulsion on the concave side of the corannulene may not be as large. However, when HX or XY approaches corannulene from the concave side, it is easy to predict that the steric interaction will be large.

The *r*_1_ values of F–X-∗-π(C_20_H_10_) are smaller than those of X–X-∗-π(C_20_H_10_) on the cv side; if *r*_1_ of the same X is compared, so is the cc side. The accepting ability of F–X should be larger than that of X–X, which results in *r*_1_(F–X-∗-π(C_20_H_10_)) < *r*_1_(X–X-∗-π(C_20_H_10_)). The X dependence in *r*_1_ can be discussed by the data in Figure 6. The *r*_1_ values of X–H-∗-π(C_20_H_10_) on the cv side seem almost constant of X–H- = F–H- < Cl–H- ≈ Br–H- ≈ I–H-, whereas the values on the cc side become larger (gradually) in the order of X–H- = F–H- < Cl–H- ≈ Br–H- < I–H-. The positive charge developed on H in X–H seems to mainly control the *r*_1_ values on the cv side, where *Qn*(H) = 0.568, 0.331, 0.218, and 0.098 for F–H, Cl–H, Br–H, and I–H, respectively, if evaluated with the NPA under the M06-2X/BSS-A//MP2/BSS-A. The *r*_1_ values become larger in the order of X = F < Cl < Br < I for both sides of cv and cc in X–X-∗-π(C_20_H_10_) and F–X-∗-π(C_20_H_10_). The atomic sizes of X must be an important factor for the X dependence on *r*_1_, where the size should be proportional to the number of electrons on X in X–X and F–X. The atomic sizes can be approximated by the vdW radii of atoms, which are 2.40, 2.94, 3.50, 3.70, and 3.96 Å for H, F, Cl, Br, and I, respectively.

In the corannulene adducts with HX of the convex side, the H-∗-π(C_20_H_10_) distances of XH-∗-π(C_20_H_10_) are HF < HCl < HBr < HI, where HX acts as the monodentate. However, this order is inversely related to the acidity of XH. The size of the halogen may be one of the reasons. The overall trend for the concave and convex sides is the same. However, it is likely on the concave side that a portion of each interaction force will be consumed by the atom on the opposite side by taking bidentate to pentadentate configuration, relative to the convex side. This is because the interaction force of the atoms on the opposite side would be weakened by the bidentate to pentadentate coordination. This means that the distance between atoms on the concave side is longer than that on the convex side.

The electron–electron repulsive factor between π(C_20_H_10_) and Y–X (Y = X and F) in Y–X-∗-π(C_20_H_10_) is also expected to play an important role in *r*_1_. The repulsion could be larger on the cc side than on the cv side in the bowl-shaped π(C_20_H_10_), resulting in the relative values of *r*_1_(cv) ≪ *r*_1_(cc). The electron density *ρ*(***r***) must be more widely extended on the cv side relative to the case of the cc side, as expected. The higher negative area on the cv side of π(C_20_H_10_) corresponds to the larger distribution of *ρ*(***r***) relative to the case of the cc side. The results shown in Figure 6 can be well understood based on the EPS shown in Figure 1, where *r*_1_ decreases as the CT interaction of the π(C_20_H_10_)→σ*(X–Y: X = X or F) type increases.

### 3.6. Meaning of the QTAIM-DFA Parameters and the Related Values

What is the meaning of the QTAIM-DFA parameters of (R, *θ*) and (*θ*_p_, *κ*_p_) for the adducts collected in Table 1? However, it seems often difficult to compare the values with those derived from other methods, since the QTAIM approach (and QTAIM-DFA) are analyzed using the values at BCP on BP, whereas analyzed values from other methods seem (very) different from the case of QTAIM-DFA. The charge of an atom (A) in a molecule calculated based on the QTAIM approach (*QTAIM*(A)) is intrinsically very different from that with NPA (*Qn*(A)), for instance [38]. Nevertheless, the meaning of the QTAIM-DFA parameters is clarified by comparing other physical parameters.

**Figure 7:**
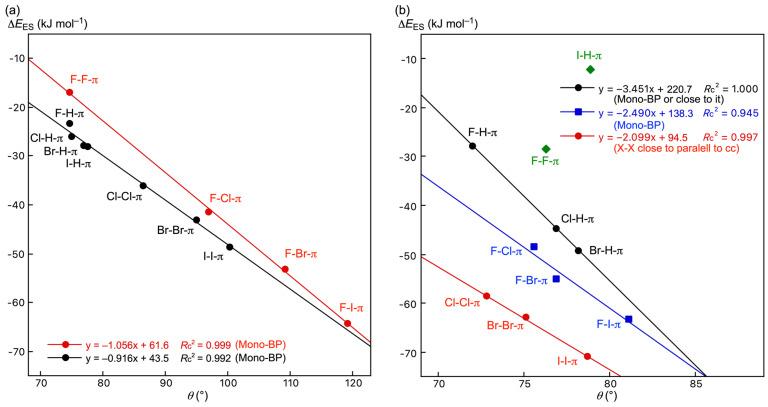
Plots of Δ*E* versus *θ* on the cv side (**a**) and cc side (**b**). The plots for the cv side were analyzed as the two correlations: the first correlation consists of X–H-∗-π(C_20_H_10_) (X = F, Cl, Br, and I) and X–X-∗-π(C_20_H_10_) (X = Cl, Br, and I), denoted by ●, and the second one of F–X-∗-π(C_20_H_10_) (X = F, Cl, Br, and I), denoted by ●. In the case of the cc side, the plots were analyzed as the four groups. The first group consists of X–H-∗-π(C_20_H_10_) (X = F, Cl, and Br) (●) and X–X-∗-π(C_20_H_10_) (X = Cl, Br, and I) (●) forms the second group. The third group contains F–X-∗-π(C_20_H_10_) (X = Cl, Br, and I) (■), while data for I–H-∗-π(C_20_H_10_) and F–F-∗-π(C_20_H_10_) (◆) deviate from the three correlations.

First of all, Δ*E* are plotted versus *θ*, separately by the cv and cc sides (see Table 1). Figure 7 shows the plots. The plots give (very) good correlations, which are shown in the figure. The plots for the cv side are shown by Figure 7a, which are analyzed as the two correlations. The first correlation consists of X–H-∗-π(C_20_H_10_) (X = F, Cl, Br, and I) and X–X-∗-π(C_20_H_10_) (X = Cl, Br, and I) (*y* = –0.916*x* + 43.5; *R*_c_^2^ = 0.992) and the second one of F–X-∗-π(C_20_H_10_) (X = F, Cl, Br, and I) (*y* = –1.056*x* + 61.6; *R*_c_^2^ = 0.999). The correlations are close with each other; therefore, they could be recognized as a correlation, where the components in all adducts are connected by only one BP for each. In the case of the cc side (Figure 7b), the plots were analyzed as the four groups. The first group consists of X–X-∗-π(C_20_H_10_) (X = Cl, Br, and I) (*y* = –2.099*x* + 94.5; *R*_c_^2^ = 0.997), while X–H-∗-π(C_20_H_10_) (X = F, Cl, and Br) forms the second group (*y* = –3.451*x* + 220.7; *R*_c_^2^ = 1.000). The third group contains F–X-∗-π(C_20_H_10_) (X = Cl, Br, and I) (*y* = –2.490*x* + 138.3; *R*_c_^2^ = 0.945), while data for I–H-∗-π(C_20_H_10_) and F–F-∗-π(C_20_H_10_) deviate from the above correlations. The X–X bonds in the first group seem close to parallel to the averaged molecular plane of C_20_H_10_. In the case of F–H-∗-π(C_20_H_10_) in the second group, F–H and π(C_20_H_10_) are connected by only one BP in the adduct. Indeed, the components of X–H-∗-π(C_20_H_10_) (X = Cl and Br) are connected by three BPs, but the structures seem close with each other. The components of F–X-∗-π(C_20_H_10_) (X = Cl, Br, and I) are connected through only one BP; therefore, the structures are very close with each other.

The *θ* values are shown to well correlate to the Δ*E* values, if the data are appropriately analyzed separately by the structures. The results may show that *θ* appears proportional to Δ*E* in the weak CS interaction region of vdW, *t*-HB, and CT-MC. However, the *θ* values of the main interactions are employed for the plots.

It is necessary to search for such a parameter that covers all interactions for those multi-BPs that connect the components. We examined the *C_ii_*^–1^ values for the purpose, since the total values of *C_ii_*^–1^ could be calculated according to Equation (18).

*C_ii_*^–1^_total_ = Σ_k_ *C_ii_*^–1^_k_ (18)

**Figure 8:**
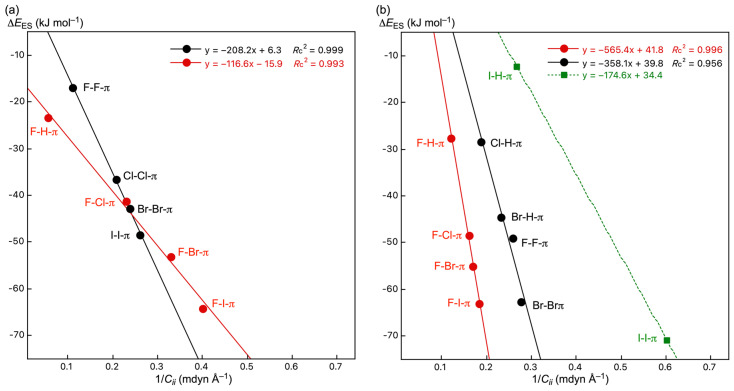
Plots of Δ*E* versus *C_ii_*^–1^ for the adducts on the cv side (**a**) and cc side (**b**). The plots for the cv side were analyzed as two correlations. Data from X–X-∗-π(C_20_H_10_) (X = F, Cl, Br, and I), shown by ●, form the first correlation, while the second correlation consists of those from F–X-∗-π(C_20_H_10_) (X = Cl, Br, and I) and F–H-∗-π(C_20_H_10_) (●). In the case of the cc side, the plots were analyzed as three groups. The first group consists of the data from X–H-∗-π(C_20_H_10_) (X = Cl and Br) and X–X-∗-π(C_20_H_10_) (X = F and Br) (●), and those from F–X-∗-π(C_20_H_10_) (X = Cl, Br, and I) and F–H-∗-π(C_20_H_10_) (●) form the second one, whereas those from I–H-∗-π(C_20_H_10_) and I–I-∗-π(C_20_H_10_) (■) seem to deviate from the correlations.

The Δ*E* values of the adducts are plotted versus *C_ii_*^–1^ or *C_ii_*^–1^_total_ for the adducts. Figure 8 shows the plot, which is analyzed separately by the cv and cc adducts. Figure 8a,b show the plots for the cv and cc adducts, respectively. The plots for the cv side were analyzed as two correlations, similarly to the case of Figure 7a. Data from X–X-∗-π(C_20_H_10_) (X = F, Cl, Br, and I) form the first correlation (*y* = –208.2*x* + 6.3; *R*_c_^2^ = 0.999), while the second correlation *y* = –116.6*x*−15.9; *R*_c_^2^ = 0.993) consists of those from F–X-∗-π(C_20_H_10_) (X = Cl, Br, and I) and F–H-∗-π(C_20_H_10_). In the case of the cc side, the plots were analyzed as three groups. The first group consists of the data from X–H-∗-π(C_20_H_10_) (X = Cl and Br) and X–X-∗-π(C_20_H_10_) (X = F and Br) (*y* = –358.1*x* + 39.8; *R*_c_^2^ = 0.956) and those from F–X-∗-π(C_20_H_10_) (X = Cl, Br, and I) and F–H-∗-π(C_20_H_10_) form the second one (*y* = –565.4*x* + 41.8; *R*_c_^2^ = 0.996), whereas those from I–H-∗-π(C_20_H_10_) and I–I-∗-π(C_20_H_10_) seem to deviate from the correlations. The I atom may interact uniquely with the cc side of π(C_20_H_10_). The strength of the interaction would extend over the range of the correlation line, perhaps due to its softness and the (very) large size.

The *C_ii_*^–1^ or *C_ii_*^–1^_total_ values are demonstrated to correlate well with the Δ*E* values, if the data are appropriately analyzed separately by the structures, again. The results show that the *C_ii_*^–1^ or *C_ii_*^–1^_total_ values are proportional to the Δ*E* values, as expected [19]. The plots in Figure 7a seem very close to those of Figure 8a, as a whole. The results may show that the *θ* values can be recognized as a parameter for Δ*E*, if only one BP connects the components of the cv adducts in the weak CS interaction region of vdW, *t*-HB, and CT-MC. In the case of the cc side, the plots in Figure 7b seem very different from those in Figure 8b, in the numbers of the plots and the members for the groups. The results must be the reflection of the different physical meanings between *θ* and *C_ii_*^–1^ (*C_ii_*^–1^_total_). Of course, the contributions from the weaker BPs are considered in *C_i_*_i_^–1^_total_ but only the strongest one is in *θ*, which must also be a very important factor to explain the differences in the plots between Figure 7b and Figure 8a.

### 3.7. Differences in the Nature of B–A-∗-π (B–A = X–H and F–X) among π of π(C_20_H_10_), π(C_24_H_12_) and π(C_6_H_6_)

The ID structures defined in Scheme 2 are optimized assuming the *C*_5v_ symmetry for the π(C_20_H_10_) adducts, the *C*_6v_ symmetry for the π(C_24_H_12_), and the *C*_2v_ symmetry for the π(C_6_H_6_) adducts. (The optimizations did not converge well under the *C*_6v_ symmetry for the π(C_6_H_6_) adducts.) The structural parameters are collected in Table S6 of the Supplementary Materials. The QTAIM functions and the similarly calculated QTAIM-DFA parameters are collected in Tables S7–S10 of the Supplementary Materials. The values of Δ*E*_ES_ for X–H-∗-π and F–X-∗-π adducts with π(C_20_H_10_), π(C_24_H_12_), and π(C_6_H_6_) are also collected in Table S11 of the Supplementary Materials. Table 2 shows the *θ* and *θ*_p_ values for the X–H-∗-π and F–X-∗-π interactions together with the Δ*θ* and Δ*θ*_p_ values. The Δ*θ* and Δ*θ*_p_ values are given from those of the π(C_6_H_6_) adducts. They are defined as Δ*θ* = (*θ* for the π(C_20_H_10_) or π(C_24_H_12_) adducts) − (*θ* for the π(C_6_H_6_) adducts) and Δ*θ*_p_ = (*θ*_p_ for the π(C_20_H_10_) or π(C_24_H_12_) adducts) − (*θ*_p_ for the π(C_6_H_6_) adducts).

The trends in *θ* and *θ*_p_ are discussed, employing the Δ*θ* and Δ*θ*_p_ values. Equations (19)–(22) summarize the trends in Δ*θ* and Δ*θ*_p_, where the values for the F–H and F–F adducts are omitted, since the trends of the values seem very different from others in some cases.

Order for Δ*θ* in X–H-∗-π:

π(C_20_H_10_: cv) (–0.7° ≤ Δ*θ* ≤ 0.1°) ≤ π(C_24_H_12_) (–0.2° ≤ Δ*θ* ≤ 0.2°) < π(C_6_H_6_) (Δ*θ* = 0.0°) < π(C_20_H_10_: cc) (2.6°≤ Δ*θ* ≤ 4.3°) (19)

Order for Δ*θ* in F–X-∗-π:

π(C_6_H_6_) (Δ*θ* = 0.0°) < π(C_24_H_12_) (0.7° ≤ Δ*θ* ≤ 2.1°) ≤ π(C_20_H_10_: cv) (1.2° ≤ Δ*θ* ≤ 3.5°) < π(C_20_H_10_: cc) (4.7° ≤ Δ*θ* ≤ 5.5°) (20)

Order for Δ*θ*_p_ in X–H-∗-π:

π(C_6_H_6_) (Δ*θ*_p_ = 0.0°) < π(C_24_H_12_) (1.6° ≤ Δ*θ*_p_ ≤ 3.5°) ≤ π(C_20_H_10_: cv) (2.3° ≤ Δ*θ*_p_ ≤ 3.6°) < π(C_20_H_10_: cc) (12.7° ≤ Δ*θ*_p_ ≤ 14.0°) (21)

Order for Δ*θ*_p_ in F–X-∗-π:

π(C_6_H_6_) (Δ*θ*_p_ = 0.0°) < π(C_24_H_12_) (3.6° ≤ Δ*θ*_p_ ≤ 7.6°) < π(C_20_H_10_: cv) (5.4° ≤ Δ*θ*_p_ ≤ 11.6°) < π(C_20_H_10_: cc) (11.6° ≤ Δ*θ*_p_ ≤ 15.4°) (22)

Equation (19) shows the order for Δ*θ* in X–H-∗-π. The order seems reasonable. However, the position of π(C_6_H_6_) should be considered, of which the order would be expected to appear between π(C_20_H_10_: cc) and π(C_24_H_12_). The order for Δ*θ* in F–X-∗-π is given in Equation (20). The order seems curious at first glance. However, the order can be understood if the order is explained separately by the partial order of π(C_6_H_6_) < π(C_24_H_12_) < π(C_20_H_10_: cv) and < π(C_20_H_10_: cc).

Equation (21) shows the order for Δ*θ*_p_ in X–H-∗-π. The order seems close to that for Δ*θ* in F–X-∗-π shown in Equation (20); therefore, the order can be explained as discussed for the trend in Equation (20). The order for Δ*θ*_p_ in F–X-∗-π is given in Equation (22). The order is very close to those in Equations (20) and (21), again. Therefore, the order is well understood by the partial order of π(C_6_H_6_) < π(C_24_H_12_) < π(C_20_H_10_: cc) < π(C_20_H_10_: cv), again.

The magnitudes of Δ*θ*_p_ in Equation (22) seem larger than those in Equation (21), as a whole, and the magnitudes of Δ*θ*_p_ in Equations (21) and (22) seem larger than the magnitudes of Δ*θ* in Equation (20). The dynamic nature of X–H-∗-π and F–X-∗-π in the adducts with π(C_20_H_10_) on both the cv and cc sides would be (very) flexible by the structural changes.

The trends in the behavior of Δ*θ* and Δ*θ*_p_ show that the process for the formation of the adducts is much more complex than expected based on the electronic structures of π(C_6_H_6_), π(C_24_H_12_), π(C_20_H_10_: cv), and π(C_20_H_10_: cc), which were simply imaged. The magnitudes of Δ*θ* and Δ*θ*_p_ for the F–H and F–F adducts with π(C_20_H_10_) and π(C_24_H_12_), from those with π(C_6_H_6_), seem much smaller than those for the other adducts. The results may show that the nature of the interactions in question would not be affected so much for the F–H and F–F adducts relative to the case of other adducts.

## 4. Conclusions

What is the nature of the XH-∗-π and YX-∗-π interactions in a distorted π-system? The nature of such interactions is elucidated, exemplified by the corannulene π-system (π(C_20_H_10_)) with QTAIM-DFA, where the π(C_20_H_10_) orbitals extend wider over the cv side but narrower on the cc side. In the optimized structures of XH-∗-π(C_20_H_10_) and YX-∗-π(C_20_H_10_) (X = F, Cl, Br, and I; Y = X and F) with MP2/BSS-A, only one side of the atom, H of X–H or X of Y–X, joins π(C_20_H_10_) on the cv side. However, both sides of the atoms connect to π(C_20_H_10_) on the cc side in some cases. In the case of the cv adducts, all XH-∗-π(C_20_H_10_) (I_Cora:cv_) interactions are predicted to have the *p*-CS/*t*-HB_nc_ nature except for FH-∗-π(C_20_H_10_), of which the nature is *p*-CS/vdW. For YX-∗-π(C_20_H_10_) (I_Cora:cv_) interactions, the *p*-CS/vdW and *p*-CS/*t*-HB_nc_ nature is predicted for YX = FF and ClCl, respectively, the *p*-CS/*t*-HB_nc_ nature is predicted for YX = BrBr, II, and FCl, and the *p*-CS/CT-MC nature is predicted for YX = FBr and FI. For the cc adducts, the predicted nature seems more complex. Therefore, the descriptions are limited to the main interactions, which are given plainly in Table 2, to avoid complexity. All XH-∗-π(C_20_H_10_) interactions are predicted to have a *p*-CS/*t*-HB_nc_ nature, irrespective of the (IA_Cora:cc_) structure for X = F and the (IIA_Cora:cc_) structure for X = Cl, Br, and I. While the FX-∗-π(C_20_H_10_) interactions are predicted to have the *p*-CS/*t*-HB_nc_ nature for X = Cl, Br, and I, the *p*-CS/vdW nature is for X = F. The optimized structures are (IA_Cora:cc_) for the former and (IIA_Cora:cc_) for the latter. In the case of the XX-∗-π(C_20_H_10_) (II_Cora:cc_) interactions, the *p*-CS/vdW nature is predicted for X = Cl and Br, whereas the *p*-CS/*t*-HB_nc_ nature is predicted for X = I. Indeed, the predicted nature of the interactions is controlled mainly by the cv and cc sides of π(C_20_H_10_) but are determined depending on the structures of the adducts. Therefore, they are more complex on the cc side. The predicted nature seems to increase in the order of X = F < Cl < Br < I for YX-∗-π(C_20_H_10_) (Y = F and X).

The differences and similarities are also clarified for the XH-∗-π and YX-∗-π interactions with the bowl-shaped π(C_20_H_10_) system and the planar π(C_6_H_6_)) and π(C_24_H_12_) systems. Indeed, the structures and the nature of the interactions seems well understood based in QTAIM-DFA, but the results show a much more complex process for the formation of the adducts than that expected based on the simply imaged electronic structures. The results will provide a useful guideline to analyze the nature of the interactions in the distorted π-systems and the adducts.

## Figures and Tables

**Table 1:** QTAIM functions and QTAIM-DFA parameters for X–H-∗-π(C_20_H_10_) and Y–X-∗-π(C_20_H_10_) (X, Y = F, Cl, Br, and I), evaluated with MP2/BSS-A ^1,2^, employing the perturbed structures generated with CIV and/or NIV.

Y–X-∗-π(C_20_H_10_) (Symmetry: Type)	*ρ*_b_(*r*_c_)	*c*∇^2^*ρ*_b_(*r*_c_) ^3^	*H*_b_(*r*_c_)	*R* ^4^	*θ* ^5^	*C_ii_*	*θ*_p_ ^6^	*κ*_p_ ^7^	Predicted Nature
	(*ea*_o_^–3^)	(au)	(au)	(au)	(°)	(Å mdyn^–1^)	(°)	(au^–1^)	
Convex side (with CIV)									
F–H-∗-π(^a^C) (*C*_1_: IA_Cora:cv_)	0.0165	0.0064	0.0018	0.0067	74.7	17.674	123.6	362.5	*p*-CS/*t*-HB_nc_
F–F-∗-π(^a^C) (*C*_s_: IA_Cora:cv_)	0.0167	0.0089	0.0024	0.0092	74.7	9.004	81.9	36.3	*p*-CS/vdw
Cl–Cl-∗-π(^a^C) (*C*_s_: IA_Cora:cv_)	0.0227	0.0089	0.0006	0.0089	86.4	4.795	122.2	198.1	*p*-CS/*t*-HB_nc_
Br–Br-∗-π(^a^C) (*C*_s_: IA_Cora:cv_)	0.0260	0.0085	−0.0007	0.0085	95.0	4.197	141.4	127.3	*r*-CS/*t*-HB_wc_
I–I-∗-π(^a^C) (*C*_s_: IA_Cora:cv_)	0.0251	0.0071	−0.0013	0.0072	100.3	3.828	147.9	141.2	*r*-CS/*t*-HB_wc_
F–Cl-∗-π(^a^C) (*C*_s_: IA_Cora:cv_)	0.0302	0.0104	−0.0013	0.0105	96.9	4.321	143.2	122.1	*r*-CS/*t*-HB_wc_
F–Br-∗-π(^a^C) (*C*_s_: IA_Cora:cv_)	0.0341	0.0097	−0.0034	0.0102	109.3	3.036	159.2	75.5	*r*-CS/CT-MC
F–I-∗-π(^a^C) (*C*_1_: IA_Cora:cv_)	0.0332	0.0079	−0.0044	0.0091	119.2	2.487	165.0	52.6	*r*-CS/CT-MC
Convex side (with NIV)									
F–H-∗-π(^a^C) (*C*_1_: IA_Cora:cv_)	0.0165	0.0064	0.0018	0.0067	74.7	102.1 ^8^	111.4	307.7	*p*-CS/*t*-HB_nc_
Cl–H-∗-π(^ab^M) (*C*_s_: IB_Cora:cv_) ^9^	0.0160	0.0057	0.0016	0.0060	74.9	82.0 ^8^	88.5	117.3	*p*-CS/vdW
Br–H-∗-π(^ab^M) (*C*_1_: IB_Cora:cv_) ^9^	0.0169	0.0058	0.0014	0.0060	76.7	60.7 ^8^	96.2	260.9	*p*-CS/*t*-HB_nc_
I–H-∗-π(^ab^M) (*C*_s_: IB_Cora:cv_)	0.0175	0.0059	0.0013	0.0060	77.5	52.2 ^8^	95.9	240.3	*p*-CS/*t*-HB_nc_
F–F-∗-π(^a^C) (*C*_s_: IA_Cora:cv_)	0.0167	0.0089	0.0024	0.0092	74.7	75.6 ^8^	81.8	35.4	*p*-CS/vdw
Cl–Cl-∗-π(^a^C) (*C*_s_: IA_Cora:cv_)	0.0227	0.0089	0.0006	0.0089	86.4	79.2 ^8^	120.6	136.5	*p*-CS/*t*-HB_nc_
Br–Br-∗-π(^a^C) (*C*_s_: IA_Cora:cv_)	0.0260	0.0085	−0.0007	0.0085	95.0	65.9 ^8^	139.3	125.3	*r*-CS/*t*-HB_wc_
I–I-∗-π(^a^C) (*C*_s_: IA_Cora:cv_)	0.0251	0.0071	−0.0013	0.0072	100.3	60.0 ^8^	145.7	129.1	*r*-CS/*t*-HB_wc_
F–Cl-∗-π(^a^C) (*C*_s_: IA_Cora:cv_)	0.0302	0.0104	−0.0013	0.0105	96.9	93.3 ^8^	141.8	101.6	*r*-CS/*t*-HB_wc_
F–Br-∗-π(^a^C) (*C*_s_: IA_Cora:cv_)	0.0341	0.0097	−0.0034	0.0102	109.3	86.9 ^8^	157.7	59.2	*r*-CS/CT-MC
F–I-∗-π(^a^C) (*C*_1_: IA_Cora:cv_)	0.0332	0.0079	−0.0044	0.0091	119.2	81.6 ^8^	164.1	49.4	*r*-CS/CT-MC
Concave side (with CIV)									
F–H-∗-π(^a^C) (*C*_1_: IA_Cora:cc_)	0.0144	0.0065	0.0021	0.0068	72.0	8.175	95.9	273.2	*p*-CS/*t*-HB_nc_
Cl–H-∗-π(^a^C) (*C*_s_: IIA_Cora:cc_)	0.0162	0.0064	0.0015	0.0065	76.9	31.667	108.6	409.5	*p*-CS/*t*-HB_nc_
Br–H-∗-π(^a^C) (C_1_: IIA_Cora:cc_)	0.0174	0.0065	0.0014	0.0066	78.2	34.149	117.2	736.5	*p*-CS/*t*-HB_nc_
I–H-∗-π(^f^C) (C_1_: IIA_Cora:cc_)	0.0172	0.0062	0.0012	0.0063	78.9	57.414	93.5	200.2	*p*-CS/*t*-HB_nc_
F–F-∗-π(^a^C) (C_s_: IIA_Cora:cc_)	0.0093	0.0048	0.0012	0.0050	76.3	12.665	84.6	6.4	*p*-CS/vdw
Br–Br-∗-π(^a^C) (C_s_: IIB_Cora:cc_)	0.0124	0.0054	0.0014	0.0056	75.4	9.442	86.3	85.5	*p*-CS/vdw
I–I-∗-π(^a^C) (C_s_: IIB_Cora:cc_)	0.0130	0.0049	0.0010	0.0050	78.7	4.975	91.2	117.9	*p*-CS/*t*-HB_nc_
F–Cl-∗-π(^a^C) (C_1_: IA_Cora:cc_)	0.0137	0.0065	0.0017	0.0067	75.6	6.162	95.0	140.9	*p*-CS/*t*-HB_nc_
F–Br-∗-π(^a^C) (C_1_: IA_Cora:cc_)	0.0139	0.0061	0.0014	0.0062	76.9	5.875	99.9	204.6	*p*-CS/*t*-HB_nc_
F–I-∗-π(^a^C) (C_s_: IA_Cora:cc_)	0.0141	0.0054	0.0008	0.0054	81.1	5.412	109.2	324.3	*p*-CS/*t*-HB_nc_
Concave side (with NIV)									
F–H-∗-π(^a^C) (*C*_1_: IA_Cora:cc_)	0.0144	0.0065	0.0021	0.0068	72.0	100.0 ^8^	100.2	282.2	*p*-CS/*t*-HB_nc_
Cl–H-∗-π(^a^C) (*C*_s_: IIA_Cora:cc_)	0.0162	0.0064	0.0015	0.0065	76.9	77.7 ^8^	125.4	955.4	*p*-CS/*t*-HB_nc_
Br–H-∗-π(^a^C) (C_1_: IIA_Cora:cc_)	0.0174	0.0065	0.0014	0.0066	78.2	59.0 ^8^	137.7	1830	*p*-CS/*t*-HB_nc_
I–H-∗-π(^f^C) (C_1_: IIA_Cora:cc_)	0.0172	0.0062	0.0012	0.0063	78.9	53.5 ^8^	92.2	344.1	*p*-CS/*t*-HB_nc_
F–F-∗-π(^a^C) (C_s_: IIA_Cora:cc_)	0.0093	0.0048	0.0012	0.0050	76.3	76.2 ^8^	89.3	305.2	*p*-CS/vdw
Cl–Cl-∗-π(^f^C) (C_1_: IIA_Cora:cc_)	0.0118	0.0054	0.0017	0.0056	72.8	97.0 ^8^	84.5	94.5	*p*-CS/vdw
Br–Br-∗-π(^a^C) (C_s_: IIB_Cora:cc_)	0.0124	0.0054	0.0014	0.0056	75.4	76.0 ^8^	88.5	98.1	*p*-CS/vdw
I–I-∗-π(^a^C) (C_s_: IIB_Cora:cc_)	0.0130	0.0049	0.0010	0.0050	78.7	70.9 ^8^	94.1	136.2	*p*-CS/*t*-HB_nc_
F–Cl-∗-π(^a^C) (C_1_: IA_Cora:cc_)	0.0137	0.0065	0.0017	0.0067	75.6	95.4 ^8^	93.8	131.4	*p*-CS/*t*-HB_nc_
F–Br-∗-π(^a^C) (C_1_: IA_Cora:cc_)	0.0139	0.0061	0.0014	0.0062	76.9	79.2 ^8^	97.1	168.1	*p*-CS/*t*-HB_nc_
F–I-∗-π(^a^C) (C_s_: IA_Cora:cc_)	0.0141	0.0054	0.0008	0.0054	81.1	76.0 ^8^	107.7	228.9	*p*-CS/*t*-HB_nc_

^1^ See text for BSS-A. ^2^ Data are given at BCP, which is shown by A-∗-π, where a one side interaction is shown if two are identical due to symmetry. ^3^ *c*∇^2^*ρ*_b_(***r***_c_) = *H*_b_(***r***_c_) − *V*_b_(***r***_c_)/2, where *c* = *ћ*^2^/8*m*. ^4^ *R* = (*x*^2^ + *y*^2^)^1/2^, where (*x*, *y*) = (*H*_b_(***r***_c_) − *V*_b_(***r***_c_)/2, *H*_b_(***r***_c_)). ^5^ *θ* = 90° − tan^–1^ (*y*/*x*). ^6^ *θ*_p_ = 90° − tan^–1^ (d*y*/d*x*). ^7^ *κ*_p_ = |d^2^*y*/d*x*^2^|/[1 + (d*y*/d*x*)^2^]^3/2^. ^8^ The frequency corresponding to the interval vibration employed to generate the perturbed structures with NIV in cm^–1^. ^9^ Perturbed structures are generated employing *w* = –0.05, –0.025, (0), 0.025, and 0.05 in Equation (4).

**Table 2:** *θ* and *θ*_p_ values for H-∗-π and X-∗-π interactions with π(C_20_H_10_), π(C_24_H_12_), and π(C_6_H_6_), evaluated with MP2/BSS-A, together with values from corresponding adducts with π(C_6_H_6_), respectively ^1^.

Y–X-∗-π(C_20_H_10_) (Symmetry: Type)	*θ* ^2^	*θ*_p_^3^	Δ*θ* ^4^	Δ*θ*_p_^5^	Y–X-∗-π(C_24_H_12_/C_6_H_6_) (Symmetry: Type)	*θ* ^2^	*θ*_p_ ^3^	Δ*θ* ^4^	Δ*θ*_p_ ^5^
	(°)	(°)	(°)	(°)		(°)	(°)	(°)	(°)
Convex side of π(C_20_H_10_)					Y–X-∗-π(C_24_H_12_)				
F–H-∗-π(^a^C) (*C*_5v_: ID_Cora:cv_) ^6,7^	66.1	68.9	−1.3	−0.6	F–H-∗-π(^a^C) (*C*_6v_: ID_Cor_) ^6,7^	67.0	68.8	−0.4	−0.7
Cl–H-∗-π(^a^C) (*C*_5v_: ID_Cora:cv_) ^6,7^	71.5	74.4	−0.7	2.3	Cl–H-∗-π(^a^C) (*C*_6v_: ID_Cor_) ^6,7^	72.0	73.7	−0.2	1.6
Br–H-∗-π(^a^C) (*C*_5v_: ID_Cora:cv_) ^6,7^	72.5	75.8	−0.3	3.1	Br–H-∗-π(^a^C) (*C*_6v_: ID_Cor_) ^6,7^	72.7	75.2	−0.1	2.5
I–H-∗-π(^a^C) (*C*_5v_: ID_Cora:cv_) ^6,7^	73.6	77.7	0.1	3.6	I–H-∗-π(^a^C) (*C*_6v_: ID_Cor_) ^6,7^	73.7	77.6	0.2	3.5
F–F-∗-π(^a^C) (*C*_5v_: ID_Cora:cv_) ^6,7^	70.5	73.5	−1.4	−1.9	F–F-∗-π(^a^C) (*C*_6v_: ID_Cor_) ^6,7^	71.3	74.9	−0.6	−0.5
F–Cl-∗-π(^a^C) (*C*_5v_: ID_Cora:cv_) ^6,7^	69.6	77.6	1.2	5.4	F–Cl-∗-π(^a^C) (*C*_6v_: ID_Cor_) ^6,7^	69.1	75.8	0.7	3.6
F–Br-∗-π(^a^C) (*C*_5v_: ID_Cora:cv_) ^6,7^	71.7	81.3	2.1	7.2	F–Br-∗-π(^a^C) (*C*_6v_: ID_Co_) ^6,7^	70.8	78.8	1.2	4.7
F–I-∗-π(^a^C) (*C*_5v_: ID_Cora:cv_) ^7,8^	76.5	90.8	3.5	11.6	F–I-∗-π(^a^C) (*C*_6v_: ID_Cor_) ^6,7^	75.1	86.8	2.1	7.6
Concave side of π(C_20_H_10_)					Y–X-∗-π(C_6_H_6_)				
F–H-∗-π(^a^C) (*C*_5v_: ID_Cora:cc_) ^6,7^	68.2	76.0	0.8	6.5	F–H-∗-π(^a^C) (*C*_6v_: ID_Bzn_) ^6,7^	67.4	69.5	–	–
Cl–H-∗-π(^a^C) (*C*_5v_: ID_Cora:cc_) ^6,7^	74.8	84.8	2.6	12.7	Cl–H-∗-π(^a^C) (*C*_6v_: ID_Bzn_) ^6,7^	72.2	72.1	–	–
Br–H-∗-π(^a^C) (*C*_5v_: ID_Cora:cc_) ^6,7^	75.9	86.8	3.1	14.1	Br–H-∗-π(^a^C) (*C*_6v_: ID_Bzn_) ^6,7^	72.8	72.7	–	–
I–H-∗-π(^a^C) (*C*_5v_: ID_Cora:cc_) ^6,7^	77.8	88.1	4.3	14.0	I–H-∗-π(^a^C) (*C*_6v_: ID_Bzn_) ^6,7^	73.5	74.1	–	–
F–F-∗-π(^a^C) (*C*_5v_: ID_Cora:cc_) ^6,7^	72.7	75.7	0.8	0.3	F–F-∗-π(^a^C) (*C*_6v_: ID_Bzn_) ^6,7^	71.9	75.4	–	–
F–Cl-∗-π(^a^C) (*C*_5v_: ID_Cora:cc_) ^6,7^	73.1	83.8	4.7	11.6	F–Cl-∗-π(^a^C) (*C*_6v_: ID_Bzn_) ^6,7^	68.4	72.2	–	–
F–Br-∗-π(^a^C) (*C*_5v_: ID_Cora:cc_) ^6,7^	74.5	86.4	4.9	12.3	F–Br-∗-π(^a^C) (*C*_6v_: ID_Bzn_) ^6,7^	69.6	74.1	–	–

^1^ See text for BSS-A. ^2^ *θ* = 90°–tan^–1^ (*y*/*x*), where (*x*, *y*) = (*H*_b_(***r***_c_)–*V*_b_(***r***_c_)/2, *H*_b_(***r***_c_)). ^3^ *θ*_p_ = 90°–tan^–1^ (d*y*/d*x*). ^4^ Δ*θ* = (*θ* for the π(C_20_H_10_) or π(C_24_H_12_) adducts) − (*θ* for the π(C_6_H_6_) adducts). ^5^ Δ*θ*_p_ = (*θ*_p_ for the π(C_20_H_10_) or π(C_24_H_12_) adducts) − (*θ*_p_ for the π(C_6_H_6_) adducts). ^6^ Predicted to be vdW interactions appearing in the *p*-CS region. ^7^ Two imaginary frequencies being predicted for each. ^8^ Predicted to be *t*-HB_nc_ interactions appeared in the *p*-CS region.

## Data Availability

Data are contained within the article or the Supplementary Materials.

## Supplementary Materials

The following are available online at https://www.mdpi.com/article/10.3390/molecules28104219/s1, Scheme S1: Classification of interactions by the signs of ∇^2^*ρ*_b_(***r***_c_) and *H*_b_(***r***_c_), together with *G*_b_(***r***_c_) and *V*_b_(***r***_c_). Scheme S2: QTAIM-DFA: Plot of *H*_b_(***r***_c_) versus *H*_b_(***r***_c_)–*V*_b_(***r***_c_)/2 for Weak to Strong Interactions. Scheme S3: Rough classification and characterization of interactions by *θ* and *θ*_p_, together with *k*_b_(***r***_c_) (= *V*_b_(***r***_c_)/*G*_b_(***r***_c_)). Scheme S4: Convex and concave sides of corannulene and definition of structural types of B–A⋯π(C_20_H_10_), to be clarified, where (A, B) = (H, X), (X, X), or (X, F) (X = F, Cl, Br and I) with structural parameters. Table S1: Proposed definitions for the classification and characterization of interactions by the signs *H*_b_(***r***_c_) and *H*_b_(***r***_c_)–*V*_b_(***r***_c_)/2 and their first derivatives, together with the tentatively proposed definitions by the characteristic points on the plots of *H*_b_(***r***_c_) versus *H*_b_(***r***_c_)–*V*_b_(***r***_c_)/2. Table S2: The structural parameters for X–H-∗-π(C_20_H_10_) and Y–X-∗-π(C_20_H_10_) (X, Y = F, Cl, Br and I), evaluated with MP2/BSS-A. Table S3: Δ*E*_ES_ and Δ*E*_ZP_ in X–H-∗-π(C_20_H_10_) and Y–X-∗-π(C_20_H_10_) (X, Y = F, Cl, Br and I), evaluated with MP2/BSS-A, together with *E*(2), calculated with the NBO analysis under M06-2X/BSS-A//MP2/BSS-A. Table S4: The *r*_BP_ and *R*_SL_ values evaluated with MP2/BSS-A for the optimized and observed structures of B–A-∗-π(C_20_H_10_), together with the Δ*r*_BP_ values. Table S5: The *r*_BP_ and *R*_SL_ values evaluated with MP2/BSS-A for the optimized and observed structures of B–A-∗-π(C_20_H_10_), together with the Δ*r*_BP_ values. Table S6: The structural parameters for the H-∗-π and X-∗-π interactions with π(C_20_H_10_), π(C_24_H_12_), and π(C_6_H_6_), (X = F, Cl, Br and I), evaluated with MP2/BSS-A, together with the values from the corresponding ones of the adducts with π(C_6_H_6_), respectively. Table S7: QTAIM functions and QTAIM-DFA parameters for X–H-∗-π(C_20_H_10_) and Y–X-∗-π(C_20_H_10_) (X, Y = F, Cl, Br, and I) on concave side, evaluated with MP2/BSS-A. Table S8: *C_ii_* and Δ*E* for X–H-∗-π(C_20_H_10_) and Y–X-∗-π(C_20_H_10_) (X, Y = F, Cl, Br, and I), evaluated with MP2/BSS-A,*^a^* employing the perturbed structures generated with CIV. Table S9: QTAIM functions and QTAIM-DFA parameters for X–H-∗-π(C_20_H_10_) and Y–X-∗-π(C_20_H_10_) (X, Y = F, Cl, Br and I) (*C*_5v_), evaluated with MP2/BSS-A. Table S10: QTAIM functions and QTAIM-DFA parameters for X–H-∗-π(C_24_H_12_) and Y–X-∗-π(C_24_H_12_) (X, Y = F, Cl, Br, and I) (*C*_6v_), evaluated with MP2/BSS-A. Table S11: QTAIM functions and QTAIM-DFA parameters for X–H-∗-π(C_6_H_6_) and Y–X-∗-π(C_6_H_6_) (X, Y = F, Cl, Br and I) (*C*_6v_), evaluated with MP2/BSS-A. Table S12: Δ*E*_ES_ for X–H-∗-π and Y–X-∗-π adducts with π(C_20_H_10_), π(C_24_H_12_), and π(C_6_H_6_) (X, Y = F, Cl, Br and I) (type ID), evaluated with MP2/BSS-A, together with those differences ΔΔ*E*_ES_. Figure S1: Polar (*R*, *θ*) coordinate representation of *H_b_*(***r***_c_) versus *H_b_*(***r***_c_)–*V_b_*(***r***_c_)/2, with (*θ*_p_, *κ*_p_) parameters. Figure S2: Plot of *H*_b_(***r***_c_) versus *w* in *r*(^1^Cl-^2^Cl) = *r*_o_(^1^Cl-^2^Cl) + *wa*_o_ for ^1^Cl-^2^Cl-^3^Cl^–^ (a) with the magnified picture of (a) (b) and that of *H*_b_(***r***_c_)–*V*_b_(***r***_c_)/2 versus *w* (c). Figure S3: Plots of Δ*E*_ZP_ versus Δ*E*_ES_ for the optimized structures of B–A-∗-π(C_20_H_10_), evaluated with MP2/BSS-A. Figure S4: Plot of *r*_BP_ (*r*_1_) versus *R*_SL_ (*r*_1_) for the optimized structures of B–A-∗-π(C_20_H_10_) with MP2/BSS-A. Figure S5: Molecular graphs for X–H-∗-π(C_20_H_10_), X–X-∗-π(C_20_H_10_) and F–X-∗-π(C_20_H_10_) at cv side, evaluated with BSS-A: F–H-∗-π(C_20_H_10_) (*C*_5v_: type ID_Cora:cv_), Cl–H-∗-π(C_20_H_10_) (*C*_5v_: type ID_Cora:cv_), Br–H-∗-π(C_20_H_10_) (*C*_5v_: type ID_Cora:cv_), I–H-∗-π(C_20_H_10_) (*C*_5v_: type ID_Cora:cv_), F–F-∗-π(C_20_H_10_) (*C*_5v_: type ID_Cora:cv_), Cl–Cl-∗-π(C_20_H_10_) (*C*_5v_: type ID_Cora:cv_), Br–Br-∗-π(C_20_H_10_) (*C*_5v_: type ID_Cora:cv_), I–I-∗-π(C_20_H_10_) (*C*_5v_: type ID_Cora:cv_), F–Cl-∗-π(C_20_H_10_) (*C*_5v_: type ID_Cora:cv_), F–Br-∗-π(C_20_H_10_) (*C*_5v_: type ID_Cora:cv_) and F–I-∗-π(C_20_H_10_) (*C*_5v_: type ID_Cora:cv_) Figure S6: Molecular graphs for X–H-∗-π(C_20_H_10_), X–X-∗-π(C_20_H_10_) and F–X-∗-π(C_20_H_10_) at cc side, evaluated with BSS-A: F–H-∗-π(C_20_H_10_) (*C*_5v_: type ID_Cora:cc_), Cl–H-∗-π(C_20_H_10_) (*C*_5v_: type ID_Cora:cc_), Br–H-∗-π(C_20_H_10_) (*C*_5v_: type ID_Cora:cc_), I–H-∗-π(C_20_H_10_) (*C*_5v_: type ID_Cora:cc_), F–F-∗-π(C_20_H_10_) (*C*_5v_: type ID_Cora:cc_), Cl–Cl-∗-π(C_20_H_10_) (*C*_5v_: type ID_Cora:cc_), Br–Br-∗-π(C_20_H_10_) (*C*_5v_: type ID_Cora:cc_), I–I-∗-π(C_20_H_10_) (*C*_5v_: type ID_Cora:cc_), F–Cl-∗-π(C_20_H_10_) (*C*_5v_: type ID_Cora:cc_), F–Br-∗-π(C_20_H_10_) (*C*_5v_: type ID_Cora:cc_ and F–I-∗-π(C_20_H_10_) (*C*_5v_: type ID_Cora:cc_). Figure S7: Plot of *q*_P_ for NIV versus *q*_P_ for CIV for the optimized structures of B–A-∗-π(C_20_H_10_) with MP2/BSS-A. References [56,57,58,59,60,61,62,63,64,65,66,67,68,69] are cited in the supplementary materials.
