# Supplementary material for: Dynamic and Static Nature of XH-∗-π and YX-∗-π (X = F, Cl, Br, and I; Y = X and F) in the Distorted π-System of Corannulene Elucidated with QTAIM Dual Functional Analysis
Source: Molecules. 2023 May 21;28(10):4219. doi: 10.3390/molecules28104219 (PMC10222803; doi:10.3390/molecules28104219)
Supplement: Supplementary file 1 [file molecules-28-04219-s001.zip › molecules-2285114-supplementary.pdf]

Supporting Information

Dynamic and Static Nature of XH-*-π and YX-*-π (X = F, Cl, Br and I; Y = X and F) in Distorted π-System of Corannulene Elucidated with QTAIM Dual Functional Analysis

Satoko Hayashi, * Takahiro Kato, Yuji Sugibayashi, and Waro Nakanishi*

Faculty of Systems Engineering, Wakayama University, 930 Sakaedani, Wakayama 640-8510, Japan. Fax: +81 73 457 8272; Tel: +81 73 457 8252; E-mail: hayashi3@sys.wakayama-u.ac.jp and nakanisi@sys.wakayama-u.ac.jp.

Table of Contents

Scheme S1. Classification of interactions by the signs of $\nabla^2\rho_b(\boldsymbol{r}_c)$ and $H_b(\boldsymbol{r}_c)$, together with $G_b(\boldsymbol{r}_c)$ and $V_b(\boldsymbol{r}_c)$. S4

Scheme S2. QTAIM-DFA: Plot of $H_b(\boldsymbol{r}_c)$ versus $H_b(\boldsymbol{r}_c) - V_b(\boldsymbol{r}_c)/2$ for Weak to Strong Interactions. S4

Scheme S3. Rough classification and characterization of interactions by θ and θ_p, together with $k_b(\boldsymbol{r}_c)$ (= $V_b(\boldsymbol{r}_c)/G_b(\boldsymbol{r}_c)$). S6

Scheme S4. Convex and concave sides of corannulene and definition of structural types of B–A---π($C_{20}H_{10}$), to be clarified, where (A, B) = (H, X), (X, X), or (X, F) (X = F, Cl, Br and I) with structural parameters. S10

Table S1. Proposed definitions for the classification and characterization of interactions by the signs $H_b(\boldsymbol{r}_c)$ and $H_b(\boldsymbol{r}_c) - V_b(\boldsymbol{r}_c)/2$ and their first derivatives, together with the tentatively proposed definitions by the characteristic points on the plots of $H_b(\boldsymbol{r}_c)$ versus $H_b(\boldsymbol{r}_c) - V_b(\boldsymbol{r}_c)/2$. S8

Table S2. The structural parameters for X–H-*-π($C_{20}H_{10}$) and Y–X-*-π($C_{20}H_{10}$) (X, Y = F, Cl, Br and I), evaluated with MP2/BSS-A. S11

Table S3. ΔE_{ES} and ΔE_{ZP} in X–H-*-π($C_{20}H_{10}$) and Y–X-*-π($C_{20}H_{10}$) (X, Y = F, Cl, Br and I), evaluated with MP2/BSS-A, together with E(2), calculated with the NBO analysis under M06-2X/BSS-A//MP2/BSS-A. S12

Table S4. The r_{BP} and R_{SL} values evaluated with MP2/BSS-A for the optimized and observed structures of B–A-*-π($C_{20}H_{10}$), together with the Δr_{BP} values. S13

Table S5. The r_{BP} and R_{SL} values evaluated with MP2/BSS-A for the optimized and observed structures of B–A-*-π($C_{20}H_{10}$), together with the Δr_{BP} values. S14

Table S6. The structural parameters for the H-*-π and X-*-π interactions with π($C_{20}H_{10}$), π($C_{24}H_{12}$) and π(C_6H_6), (X = F, Cl, Br and I), evaluated with MP2/BSS-A, together with the values from the corresponding ones of the adducts with π(C_6H_6), respectively. S15

Table S7. QTAIM functions and QTAIM-DFA parameters for X–H-*-π($C_{20}H_{10}$) and Y–X-*-π($C_{20}H_{10}$) (X, Y = F, Cl, Br, and I) on concave side, evaluated with MP2/BSS-A. S16

Table S8. C_{ii} and ΔE for X–H-*-π($C_{20}H_{10}$) and Y–X-*-π($C_{20}H_{10}$) (X, Y = F, Cl, Br, and I), evaluated with MP2/BSS-A,[a] employing the perturbed structures generated with CIV. S17

Table S9. QTAIM functions and QTAIM-DFA parameters for X–H-*-π($C_{20}H_{10}$) and Y–X-*-π($C_{20}H_{10}$) (X, Y = F, Cl, Br and I) ($C_{5\text{v}}$), evaluated with MP2/BSS-A. S18

Table S10. QTAIM functions and QTAIM-DFA parameters for X–H-*-π($C_{24}H_{12}$) and Y–X-*-π($C_{24}H_{12}$) (X, Y = F, Cl, Br, and I) ($C_{6\text{v}}$), evaluated with MP2/BSS-A. S19

Table S11. QTAIM functions and QTAIM-DFA parameters for X–H-*-π(C_6H_6) and Y–X-*-π(C_6H_6) (X, Y = F, Cl, Br and I) ($C_{6\text{v}}$), evaluated with MP2/BSS-A. S19

Table S12. ΔE_{ES} for X–H-*-π and Y–X-*-π adducts with π($C_{20}H_{10}$), π($C_{24}H_{12}$) and π(C_6H_6) (X, Y = F, Cl, Br and I) (type ID), evaluated with MP2/BSS-A, together with those differences $\Delta\Delta E_{\text{ES}}$. S20

Figure S1. Polar (R, θ) coordinate representation of $H_b(\boldsymbol{r}_c)$ versus $H_b(\boldsymbol{r}_c) - V_b(\boldsymbol{r}_c)/2$, with ($\theta_p$, κ_p) parameters. S5

Figure S2. Plot of $H_b(\boldsymbol{r}_c)$ versus w in r(¹Cl-²Cl) = r_o(¹Cl-²Cl) + wa_o for ¹Cl-²Cl-³Cl⁻ (a) with the magnified picture of (a) (b) and that of $H_b(\boldsymbol{r}_c) - V_b(\boldsymbol{r}_c)/2$ versus w (c). S7

Figure S3. Plots of ΔE_{ZP} versus ΔE_{ES} for the optimized structures of B–A-*-π($C_{20}H_{10}$), evaluated with MP2/BSS-A. S21

Figure S4. Plot of r_{BP} (r_1) versus R_{SL} (r_1) for the optimized structures of B–A-*-π($C_{20}H_{10}$) with MP2/BSS-A. S21

Figure S5. Molecular graphs for X–H-*-π($C_{20}H_{10}$), X–X-*-π($C_{20}H_{10}$) and F–X-*-π($C_{20}H_{10}$) at cv side, evaluated with BSS-A: F–H-*-π($C_{20}H_{10}$) ($C_{5\text{v}}$: type $\text{ID}_{\text{Cora:cv}}$), Cl–H-*-π($C_{20}H_{10}$) ($C_{5\text{v}}$: type $\text{ID}_{\text{Cora:cv}}$), Br–H-*-π($C_{20}H_{10}$) ($C_{5\text{v}}$: type $\text{ID}_{\text{Cora:cv}}$), I–H-*-π($C_{20}H_{10}$) ($C_{5\text{v}}$: type $\text{ID}_{\text{Cora:cv}}$), F–F-*-π($C_{20}H_{10}$) ($C_{5\text{v}}$: type $\text{ID}_{\text{Cora:cv}}$), Cl–Cl-*-π($C_{20}H_{10}$) ($C_{5\text{v}}$: type $\text{ID}_{\text{Cora:cv}}$), Br–Br-*-π($C_{20}H_{10}$) ($C_{5\text{v}}$: type $\text{ID}_{\text{Cora:cv}}$), I–I-*-π($C_{20}H_{10}$) ($C_{5\text{v}}$: type $\text{ID}_{\text{Cora:cv}}$), F–Cl-*-π($C_{20}H_{10}$) ($C_{5\text{v}}$: type $\text{ID}_{\text{Cora:cv}}$), F–Br-*-π($C_{20}H_{10}$) ($C_{5\text{v}}$: type $\text{ID}_{\text{Cora:cv}}$) and F–I-*-π($C_{20}H_{10}$) ($C_{5\text{v}}$: type $\text{ID}_{\text{Cora:cv}}$). S22

Figure S6. Molecular graphs for X–H-*-π($C_{20}H_{10}$), X–X-*-π($C_{20}H_{10}$) and F–X-*-π($C_{20}H_{10}$) at cc side, evaluated with BSS-A: F–H-*-π($C_{20}H_{10}$) ($C_{5\text{v}}$: type $\text{ID}_{\text{Cora:cc}}$), Cl–H-*-π($C_{20}H_{10}$) ($C_{5\text{v}}$: type $\text{ID}_{\text{Cora:cc}}$), Br–H-*-π($C_{20}H_{10}$) ($C_{5\text{v}}$: type $\text{ID}_{\text{Cora:cc}}$), I–H-*-π($C_{20}H_{10}$) ($C_{5\text{v}}$: type $\text{ID}_{\text{Cora:cc}}$), F–F-*-π($C_{20}H_{10}$) ($C_{5\text{v}}$: type $\text{ID}_{\text{Cora:cc}}$), Cl–Cl-*-π($C_{20}H_{10}$) ($C_{5\text{v}}$: type $\text{ID}_{\text{Cora:cc}}$), Br–Br-*-π($C_{20}H_{10}$) ($C_{5\text{v}}$: type $\text{ID}_{\text{Cora:cc}}$), I–I-*-π($C_{20}H_{10}$) ($C_{5\text{v}}$: type $\text{ID}_{\text{Cora:cc}}$), F–Cl-*-π($C_{20}H_{10}$) ($C_{5\text{v}}$: type $\text{ID}_{\text{Cora:cc}}$), F–Br-*-π($C_{20}H_{10}$) ($C_{5\text{v}}$: type $\text{ID}_{\text{Cora:cc}}$ and F–I-*-π($C_{20}H_{10}$) ($C_{5\text{v}}$: type $\text{ID}_{\text{Cora:cc}}$). S23

Figure S7. Plot of θ_p for NIV versus θ_p for CIV for the optimized structures of B–A-*-π($C_{20}H_{10}$) with MP2/BSS-A. F–H-*-π(ᵃC) (C_1: $\text{IA}_{\text{Cora:cv}}$), Cl–H-*-π(ᵃC) ($C_s$: $\text{IIA}_{\text{Cora:cc}}$), and Br–H-*-π(ᵃC) ($C_1$: $\text{IIA}_{\text{Cora:cc}}$) are omitted. S24

Computation information and geometries of compounds S25–S71

QTAIM Dual Functional Analysis (QTAIM-DFA)

The bond critical point (BCP; *) is an important concept in QTAIM. The BCP of $(\omega, \sigma) = (3, -1)$ [38,39] is a point along the bond path (BP) at the interatomic surface, where charge density $\rho(\boldsymbol{r})$ reaches a minimum. It is donated by $\rho_b(\boldsymbol{r}_c)$, so are other QTAIM functions, such as the total electron energy densities $H_b(\boldsymbol{r}_c)$, potential energy densities $V_b(\boldsymbol{r}_c)$ and kinetic energy densities $G_b(\boldsymbol{r}_c)$ at the BCPs. A chemical bond or interaction between A and B is denoted by A–B, which corresponds to the BP between A and B in QTAIM. We will use A-*-B for BP, where the asterisk emphasizes the presence of a BCP in A–B.

The sign of the Laplacian $\rho_b(\boldsymbol{r}_c)$ ($\nabla^2\rho_b(\boldsymbol{r}_c)$) indicates that $\rho_b(\boldsymbol{r}_c)$ is depleted or concentrated with respect to its surrounding, since $\nabla^2\rho_b(\boldsymbol{r}_c)$ is the second derivative of $\rho_b(\boldsymbol{r}_c)$. $\rho_b(\boldsymbol{r}_c)$ is locally depleted relative to the average distribution around $\boldsymbol{r}_c$ if $\nabla^2\rho_b(\boldsymbol{r}_c) > 0$, but it is concentrated when $\nabla^2\rho_b(\boldsymbol{r}_c) < 0$. Total electron energy densities at BCPs ($H_b(\boldsymbol{r}_c)$) must be a more appropriate measure for weak interactions on the energy basis [35–37,38,39,56–69]. $H_b(\boldsymbol{r}_c)$ are the sum of kinetic energy densities ($G_b(\boldsymbol{r}_c)$) and potential energy densities ($V_b(\boldsymbol{r}_c)$) at BCPs, as shown in Equation (S1). Electrons at BCPs are stabilized when $H_b(\boldsymbol{r}_c) < 0$, therefore, interactions exhibit the covalent nature in this region, whereas they exhibit no covalency if $H_b(\boldsymbol{r}_c) > 0$, due to the destabilization of electrons at BCPs under the conditions [38,39]. Equation (S2) represents the relation between $\nabla^2\rho_b(\boldsymbol{r}_c)$ and $H_b(\boldsymbol{r}_c)$, together with $G_b(\boldsymbol{r}_c)$ and $V_b(\boldsymbol{r}_c)$, which is closely related to the virial theorem.

$$H_b(\boldsymbol{r}_c) = G_b(\boldsymbol{r}_c) + V_b(\boldsymbol{r}_c) \quad \text{(S1)}$$

$$(\hbar^2/8m)\nabla^2\rho_b(\boldsymbol{r}_c) = H_b(\boldsymbol{r}_c) - V_b(\boldsymbol{r}_c)/2 \quad \text{(S2)}$$

$$= G_b(\boldsymbol{r}_c) + V_b(\boldsymbol{r}_c)/2 \quad \text{(S2')}$$

Interactions are classified by the signs of $\nabla^2\rho_b(\boldsymbol{r}_c)$ and $H_b(\boldsymbol{r}_c)$. Interactions in the region of $\nabla^2\rho_b(\boldsymbol{r}_c) < 0$ are called shared-shell (SS) interactions and they are closed-shell (CS) interactions for $\nabla^2\rho_b(\boldsymbol{r}_c) > 0$. $H_b(\boldsymbol{r}_c)$ must be negative when $\nabla^2\rho_b(\boldsymbol{r}_c) < 0$, since $H_b(\boldsymbol{r}_c)$ are larger than $(\hbar^2/8m)\nabla^2\rho_b(\boldsymbol{r}_c)$ by $V_b(\boldsymbol{r}_c)/2$ with negative $V_b(\boldsymbol{r}_c)$ at all BCPs (Equation (S2)). Consequently, $\nabla^2\rho_b(\boldsymbol{r}_c) < 0$ and $H_b(\boldsymbol{r}_c) < 0$ for the SS interactions. The CS interactions are especially called *pure* CS interactions for $H_b(\boldsymbol{r}_c) > 0$ and $\nabla^2\rho_b(\boldsymbol{r}_c) > 0$, since electrons at BCPs are depleted and destabilized under the conditions.[SA1a] Electrons in the intermediate region between SS and *pure* CS, which belong to CS, are locally depleted but stabilized at BCPs, since $\nabla^2\rho_b(\boldsymbol{r}_c) > 0$ but $H_b(\boldsymbol{r}_c) < 0$ [38]. We call the interactions in this region *regular* CS [35,36], when it is necessary to distinguish from *pure* CS. The role of $\nabla^2\rho_b(\boldsymbol{r}_c)$ in the classification can be replaced by $H_b(\boldsymbol{r}_c) - V_b(\boldsymbol{r}_c)/2$, since $(\hbar^2/8m)\nabla^2\rho_b(\boldsymbol{r}_c) = H_b(\boldsymbol{r}_c) - V_b(\boldsymbol{r}_c)/2$ (Equation (S2)). Scheme S1 summarizes the classification.

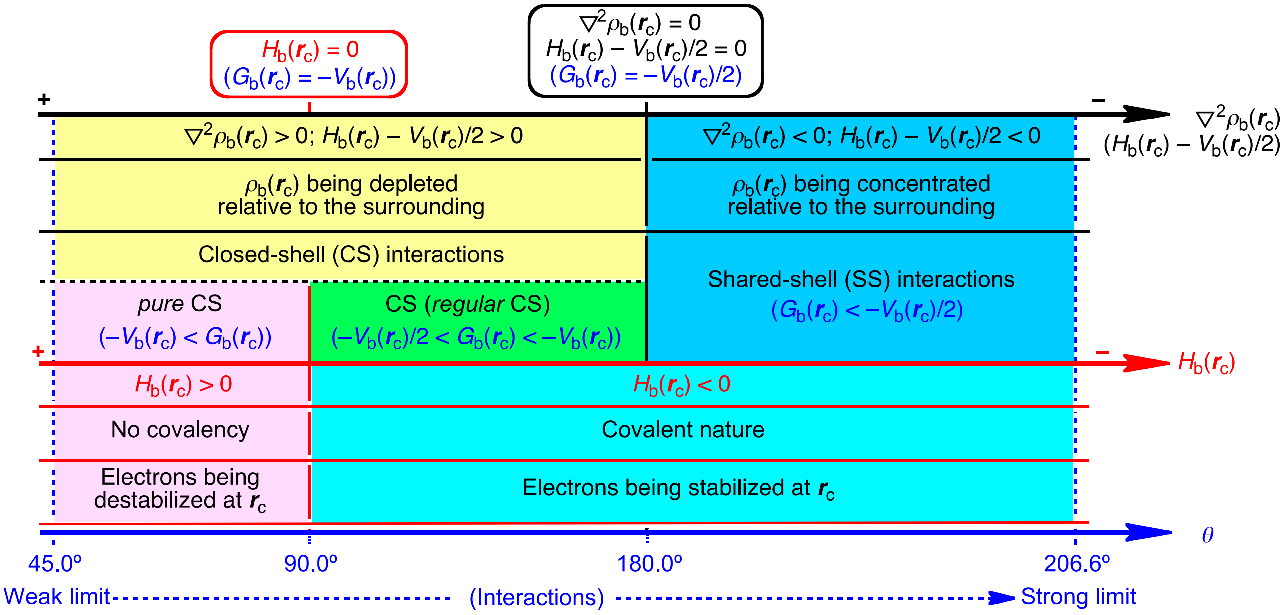

Scheme S1. Classification of interactions by the signs of $\nabla^2\rho_b(r_c)$ and $H_b(r_c)$, together with $G_b(r_c)$ and $V_b(r_c)$.

We proposed QTAIM-DFA by plotting $H_b(r_c)$ versus $H_b(r_c) - V_b(r_c)/2$ (= $(\hbar^2/8m)\nabla^2\rho_b(r_c)$) [65], after the proposal of $H_b(r_c)$ versus $\nabla^2\rho_b(r_c)$ [35,36]. Both axes in the plot of the former are given in energy unit, therefore, distances on the (x, y) (= $(H_b(r_c) - V_b(r_c)/2, H_b(r_c))$) plane can be expressed in the energy unit, which provides an analytical development. QTAIM-DFA incorporates the classification of interactions by the signs of $\nabla^2\rho_b(r_c)$ and $H_b(r_c)$. Scheme S2 summarizes the QTAIM-DFA treatment. Interactions of *pure* CS appear in the first quadrant, those of *regular* CS in the fourth quadrant and SS interactions do in the third quadrant. No interactions appear in the second one.

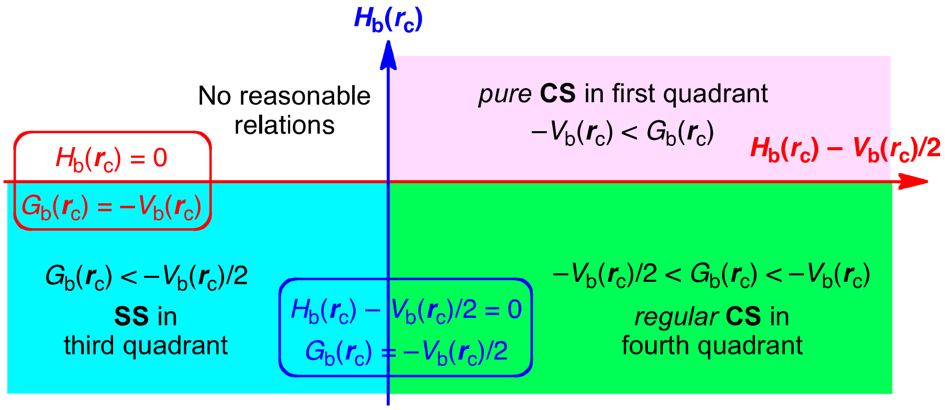

Scheme S2. QTAIM-DFA: Plot of $H_b(r_c)$ versus $H_b(r_c) - V_b(r_c)/2$ for Weak to Strong Interactions.

In our treatment, data for perturbed structures around fully optimized structures are also employed for the plots, together with the fully optimized ones (see Fig. S1) [35–37]. We proposed the concept of the "dynamic nature of interaction" originated from the perturbed structures. The behavior of interactions at the fully optimized structures corresponds to "the static nature of interactions", whereas that containing perturbed structures exhibit the "dynamic nature of interaction" as explained below.

The method to generate the perturbed structures is discussed later. Plots of $H_b(\mathbf{r}_c)$ versus $H_b(\mathbf{r}_c) - V_b(\mathbf{r}_c)/2$ are analyzed employing the polar coordinate (R, θ) representation with (θ_p, κ_p) parameters [35–37]. Fig. S1 explains the treatment. R in (R, θ) is defined by Equation (S3) and given in the energy unit. Indeed, R does not correspond to the usual interaction energy, but it does to the local energy at BCP, expressed by $[(H_b(\mathbf{r}_c))^2 + (H_b(\mathbf{r}_c) - V_b(\mathbf{r}_c)/2)^2]^{1/2}$ in the plot (cf: Equation (S3)), where $R = 0$ for the enough large interaction distance. The plots show a spiral stream, as a whole. θ in (R, θ) defined by Equation (S4), measured from the y-axis, controls the spiral stream of the plot. Each plot for an interaction shows a specific curve, which provides important information of the interaction (see Fig. S1). The curve is expressed by θ_p and κ_p. While θ_p, defined by Equation (S5) and measured from the y-direction, corresponds to the tangent line of a plot, where θ_p is calculated employing data of the perturbed structures with a fully-optimized structure and κ_p is the curvature of the plot (Equation (S6)). While (R, θ) correspond to the static nature, (θ_p, κ_p) represent the dynamic nature of interactions. We call (R, θ) and (θ_p, κ_p) QTAIM-DFA parameters, whereas $\rho_b(\mathbf{r}_c)$, $\nabla^2\rho_b(\mathbf{r}_c)$, $G_b(\mathbf{r}_c)$, $V_b(\mathbf{r}_c)$, $H_b(\mathbf{r}_c)$ and $H_b(\mathbf{r}_c) - V_b(\mathbf{r}_c)/2$ belong to QTAIM functions. $k_b(\mathbf{r}_c)$, defined by Equation (S7), is an QTAIM function but it will be treated as if it were an QTAIM-DFA parameter, if suitable.

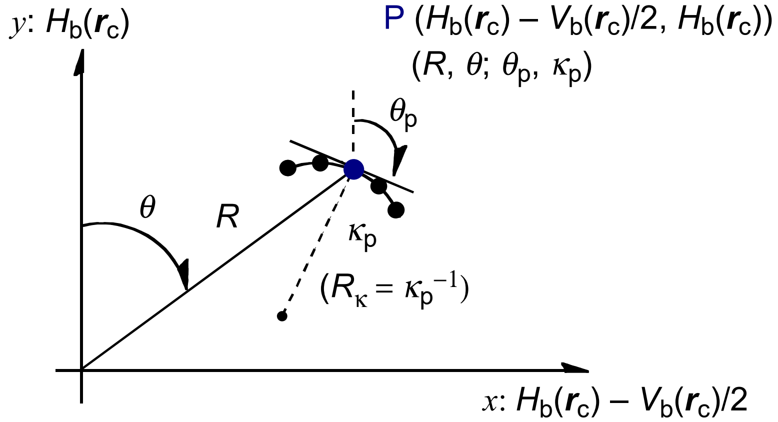

Figure S1. Polar (R, θ) coordinate representation of $H_b(\mathbf{r}_c)$ versus $H_b(\mathbf{r}_c) - V_b(\mathbf{r}_c)/2$, with ($\theta_p$, κ_p) parameters.

$R = (x^2 + y^2)^{1/2}$ (S3)

$\theta = 90^\circ - \tan^{-1}(y/x)$ (S4)

$\theta_p = 90^\circ - \tan^{-1}(dy/dx)$ (S5)

$\kappa_p = |d^2y/dx^2| / [1 + (dy/dx)^2]^{3/2}$ (S6)

$k_b(\mathbf{r}_c) = V_b(\mathbf{r}_c)/G_b(\mathbf{r}_c)$ (S7)

where $(x, y) = (H_b(\mathbf{r}_c) - V_b(\mathbf{r}_c)/2, H_b(\mathbf{r}_c))$

Criteria for Classification of Interactions: Behavior of Typical Interactions Elucidated by QTAIM-DFA

$H_b(\boldsymbol{r}_c)$ are plotted versus $H_b(\boldsymbol{r}_c) - V_b(\boldsymbol{r}_c)/2$ for typical interactions in vdW (van der Waals interactions), HBs (hydrogen bonds), CT-MCs (molecular complexes through charge transfer), X_3^- (trihalide ions), CT-TBPs (trigonal bipyramidal adducts through charge-transfer), Cov-w (weak covalent bonds) and Cov-s (strong covalent bonds) [35–37]. Rough criteria are obtained by applying QTAIM-DFA, after the analysis of the plots for the typical interactions according to Equations (S3)–(S7). Scheme S3 shows the rough criteria, which are accomplished by the θ and θ_p values, together with the values of $k_b(\boldsymbol{r}_c)$. The criteria will be employed to discuss the nature of interactions in question, as a reference.

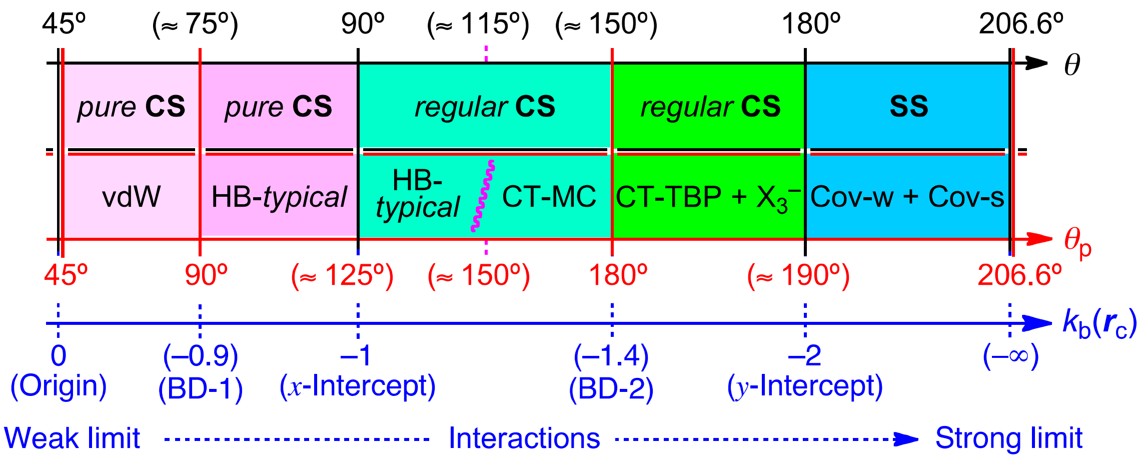

Scheme S3. Rough classification and characterization of interactions by θ and θ_p, together with $k_b(\boldsymbol{r}_c)$ (= $V_b(\boldsymbol{r}_c)/G_b(\boldsymbol{r}_c)$).

Characterization of interactions

The characterization of interactions is explained employing $[^1Cl\text{-}^2Cl\text{-}^3Cl]^-$. The wide range of the perturbed structures were generated by partially optimizing $r(^2Cl\text{-}^3Cl)$ in $[^1Cl\text{-}^2Cl\text{-}^3Cl]^-$, assuming the $C_{\infty v}$ symmetry, with $r(^1Cl\text{-}^2Cl)$ being fixed in the wide range. The partial optimization method is called POM [35,36,65]. The QTAIM functions, such as $V_b(\boldsymbol{r}_c)$, $G_b(\boldsymbol{r}_c)$, $H_b(\boldsymbol{r}_c)$, $H_b(\boldsymbol{r}_c) - V_b(\boldsymbol{r}_c)/2$ are calculated at BCPs for the wide varieties of the perturbed structures of $[^1Cl\text{-}^2Cl\text{-}^3Cl]^-$. $H_b(\boldsymbol{r}_c) - V_b(\boldsymbol{r}_c)/2$ and $H_b(\boldsymbol{r}_c)$ are plotted versus the interaction distances $r(^1Cl\text{-}^2Cl)$ in the perturbed structures of $[^1Cl\text{-}^2Cl\text{-}^3Cl]^-$, in the wide range. Fig. S2 shows the plots. Each plot is analyzed using a regression curve of the ninth function and the first derivative of each regression curve is obtained. As shown in Fig. S2, the maximum value of $H_b(\boldsymbol{r}_c)$ ($dH_b(\boldsymbol{r}_c)/dr = 0$) is defined as the borderline between vdW and t-HB interactions. Similarly, the maximum value of $H_b(\boldsymbol{r}_c) - V_b(\boldsymbol{r}_c)/2$ ($d(H_b(\boldsymbol{r}_c) - V_b(\boldsymbol{r}_c)/2)/dr = 0$) does to the borderline between CT-MC and CT-TBP. However, it seems difficult to find a characteristic

point corresponding to the borderline between t-HB and CT-MC in nature. Therefore, the borderline is tentatively given by θ_p = 150º based on the expectation form the experimental results, where θ_p is defined by [90º – tan^{-1}[d$H_b(\boldsymbol{r}_c)$/d($H_b(\boldsymbol{r}_c) - V_b(\boldsymbol{r}_c)/2$)]] in the plot of $H_b(\boldsymbol{r}_c)$ versus $H_b(\boldsymbol{r}_c) - V_b(\boldsymbol{r}_c)/2$. The proposed classification and characterization of interactions, by means of the QTAIM functions of $H_b(\boldsymbol{r}_c)$, $H_b(\boldsymbol{r}_c) - V_b(\boldsymbol{r}_c)/2$, $G_b(\boldsymbol{r}_c)$ and/or $V_b(\boldsymbol{r}_c)$, are summarized in Table S1. The plot of $H_b(\boldsymbol{r}_c) - V_b(\boldsymbol{r}_c)/2$ versus w in Fig. S2 is essentially the same as that of $\nabla^2\rho_b(\boldsymbol{r}_c)$ versus d(H---F) in X–H---F–Y, presented by Espinosa and co-workers [68,69].

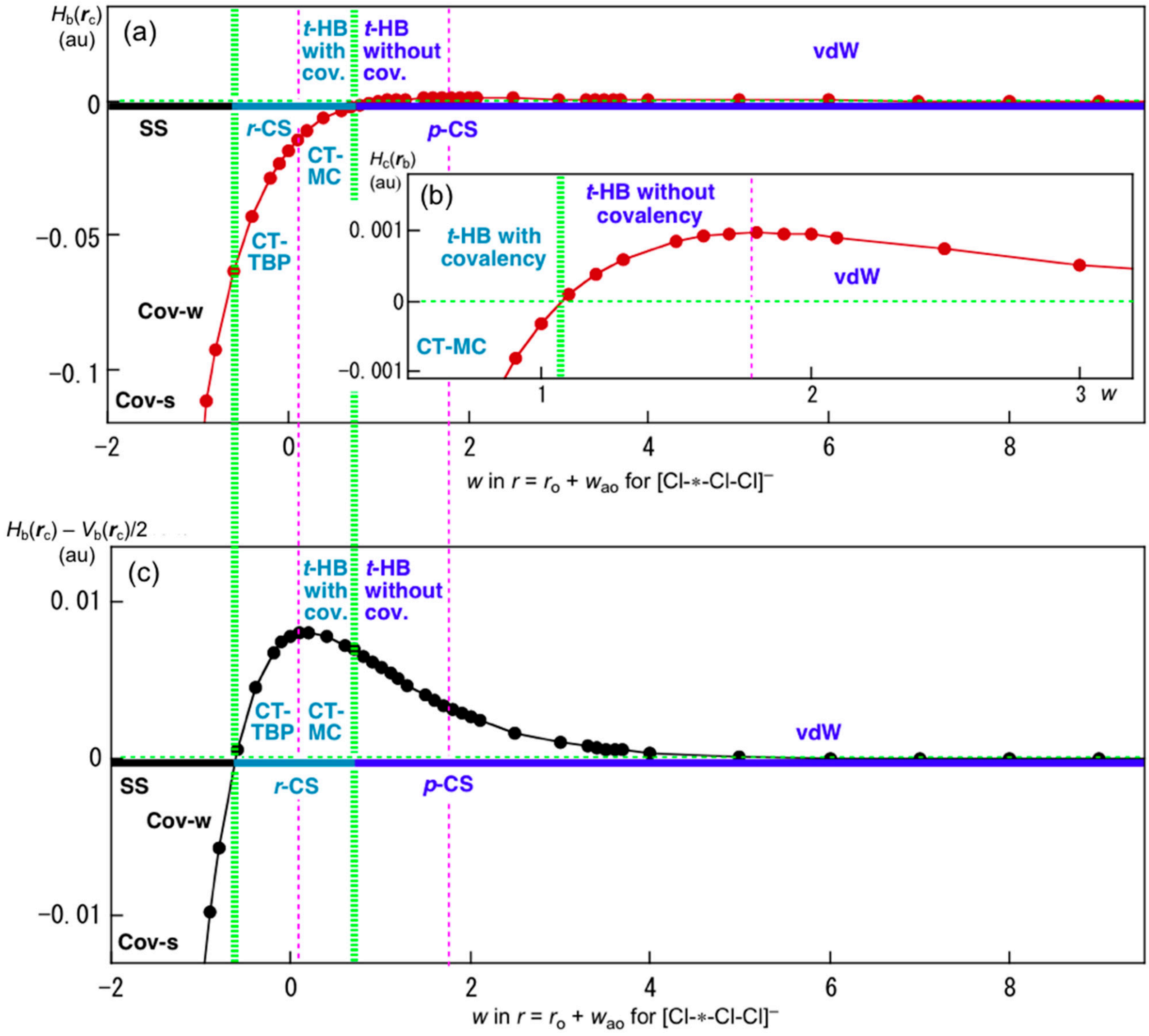

Figure S2. Plot of $H_b(\boldsymbol{r}_c)$ versus w in r(^{1}Cl-^{2}Cl) = r_o(^{1}Cl-^{2}Cl) + wa_o for ^{1}Cl-^{2}Cl-^{3}Cl$^-$ (a) with the magnified picture of (a) (b) and that of $H_b(\boldsymbol{r}_c) - V_b(\boldsymbol{r}_c)/2$ versus w (c). Typical hydrogen bonds without covalency and typical hydrogen bonds with covalency are abbreviated as t-HB without cov. and t-HB with cov., respectively, whereas Cov-w and Cov-s stand for weak covalent bonds and strong covalent bonds, respectively.

Table S1. Proposed definitions for the classification and characterization of interactions by the signs $H_b(\boldsymbol{r}_c)$ and $H_b(\boldsymbol{r}_c) - V_b(\boldsymbol{r}_c)/2$ and their first derivatives, together with the tentatively proposed definitions by the characteristic points on the plots of $H_b(\boldsymbol{r}_c)$ versus $H_b(\boldsymbol{r}_c) - V_b(\boldsymbol{r}_c)/2$. The tentatively proposed definitions are shown by italic. The requirements for the interactions are also shown.

ChP/Interaction	Requirements by $H_b(\boldsymbol{r}_c)$ and $V_b(\boldsymbol{r}_c)$	Requirements by $G_b(\boldsymbol{r}_c)$ and $V_b(\boldsymbol{r}_c)$
Origin	$H_b(\boldsymbol{r}_c) - V_b(\boldsymbol{r}_c)/2 = 0$; $H_b(\boldsymbol{r}_c) = 0$	$G_b(\boldsymbol{r}_c) = 0$; $V_b(\boldsymbol{r}_c) = 0$
vdW	$H_b(\boldsymbol{r}_c) > 0$; $\mathrm{d}H_b(\boldsymbol{r}_c)/\mathrm{d}(-r) > 0$	$G_b(\boldsymbol{r}_c) > -V_b(\boldsymbol{r}_c)$; $\mathrm{d}G_b(\boldsymbol{r}_c)/\mathrm{d}(-r) > -\mathrm{d}V_b(\boldsymbol{r}_c)/\mathrm{d}(-r)$
Borderline (BD-1)	$H_b(\boldsymbol{r}_c) > 0$; $\mathrm{d}H_b(\boldsymbol{r}_c)/\mathrm{d}(-r) = 0$	$G_b(\boldsymbol{r}_c) > -V_b(\boldsymbol{r}_c)$; $\mathrm{d}G_b(\boldsymbol{r}_c)/\mathrm{d}(-r) = -\mathrm{d}V_b(\boldsymbol{r}_c)/\mathrm{d}(-r)$
t-HB$_{\text{with no covalency}}$	$H_b(\boldsymbol{r}_c) > 0$; $\mathrm{d}H_b(\boldsymbol{r}_c)/\mathrm{d}(-r) < 0$	$G_b(\boldsymbol{r}_c) > -V_b(\boldsymbol{r}_c)$; $\mathrm{d}G_b(\boldsymbol{r}_c) < -\mathrm{d}V_b(\boldsymbol{r}_c)$
Borderline (x-intercept)	$H_b(\boldsymbol{r}_c) = 0$ ($\theta_p{}^a = 125°$)	$G_b(\boldsymbol{r}_c) = -V_b(\boldsymbol{r}_c)$ ($\theta_p{}^a = 125°$)
t-HB$_{\text{with covalency}}$	$H_b(\boldsymbol{r}_c) < 0$; ($125° <$) $\theta_p{}^a < 150°$	$G_b(\boldsymbol{r}_c) < -V_b(\boldsymbol{r}_c)$; ($125° <$) $\theta_p{}^b < 150°$
Borderline (Tentative)	$\theta_p{}^a = 150°$	$\theta_p{}^b = 150°$
CT-MC	$\mathrm{d}(H_b(\boldsymbol{r}_c) - V_b(\boldsymbol{r}_c)/2)/\mathrm{d}(-r) > 0$;	$\mathrm{d}G_b(\boldsymbol{r}_c) > \mathrm{d}V_b(\boldsymbol{r}_c)/2$;
	$150° < \theta_p{}^a < 180°$	$150° < \theta_p{}^a < 180°$
Borderline (BD-2)	$\mathrm{d}(H_b(\boldsymbol{r}_c) - V_b(\boldsymbol{r}_c)/2)/\mathrm{d}(-r) = 0$	$2\mathrm{d}G_b(\boldsymbol{r}_c)/\mathrm{d}(-r) = -\mathrm{d}V_b(\boldsymbol{r}_c)/\mathrm{d}(-r)$
	($H_b(\boldsymbol{r}_c) - V_b(\boldsymbol{r}_c)/2 > 0$; $H_b(\boldsymbol{r}_c) < 0$)	($-V_b(\boldsymbol{r}_c)/2 < G_b(\boldsymbol{r}_c) < -V_b(\boldsymbol{r}_c)$)
CT-TBP with X_3^-	$\mathrm{d}(H_b(\boldsymbol{r}_c) - V_b(\boldsymbol{r}_c)/2)/\mathrm{d}(-r) < 0$	$2\mathrm{d}G_b(\boldsymbol{r}_c)/\mathrm{d}(-r) < -\mathrm{d}V_b(\boldsymbol{r}_c)/\mathrm{d}(-r)$
	($H_b(\boldsymbol{r}_c) - V_b(\boldsymbol{r}_c)/2 > 0$; $H_b(\boldsymbol{r}_c) < 0$)	($-V_b(\boldsymbol{r}_c)/2 < G_b(\boldsymbol{r}_c) < -V_b(\boldsymbol{r}_c)$)
Borderline (y-intercept)	$H_b(\boldsymbol{r}_c) - V_b(\boldsymbol{r}_c)/2 = 0$ ($H_b(\boldsymbol{r}_c) < 0$)	$G_b(\boldsymbol{r}_c) = -V_b(\boldsymbol{r}_c)/2$ ($G_b(\boldsymbol{r}_c) < -V_b(\boldsymbol{r}_c)$)
Cov-w	$H_b(\boldsymbol{r}_c) - V_b(\boldsymbol{r}_c)/2 < 0$; $R^c < 0.15$ *au*	$G_b(\boldsymbol{r}_c) < -V_b(\boldsymbol{r}_c)/2$; $R^c < 0.15$ *au*
Borderline (Tentative)	$R^c = 0.15$ *au*	$R^d = 0.15$ *au*
Cov-s	$H_b(\boldsymbol{r}_c) - V_b(\boldsymbol{r}_c)/2 < 0$; $R^c > 0.15$ *au*	$G_b(\boldsymbol{r}_c) < -V_b(\boldsymbol{r}_c)/2$; $R^d > 0.15$ *au*

[a] $\theta_p = 90° - \tan^{-1}[\mathrm{d}H_b(\boldsymbol{r}_c)/\mathrm{d}(H_b(\boldsymbol{r}_c) - V_b(\boldsymbol{r}_c)/2)]$, $\theta_p = 125°$ is tentatively given for $\theta = 90°$, where θ is defined by $90° - \tan^{-1}[H_b(\boldsymbol{r}_c)/(H_b(\boldsymbol{r}_c) - V_b(\boldsymbol{r}_c)/2)]$ with $H_b(\boldsymbol{r}_c) = 0$. [b] $\theta_p = 90° - \tan^{-1}[\mathrm{d}(G_b(\boldsymbol{r}_c) + V_b(\boldsymbol{r}_c))/\mathrm{d}(G_b(\boldsymbol{r}_c) + V_b(\boldsymbol{r}_c)/2)]$, $\theta_p = 125°$ is tentatively given for $\theta = 90°$, where θ is defined by $90° - \tan^{-1}[(G_b(\boldsymbol{r}_c) + V_b(\boldsymbol{r}_c))/(G_b(\boldsymbol{r}_c) + V_b(\boldsymbol{r}_c)/2)]$ with $(G_b(\boldsymbol{r}_c) + V_b(\boldsymbol{r}_c)) = 0$]. [c] $R = [(H_b(\boldsymbol{r}_c) - V_b(\boldsymbol{r}_c)/2)^2 + (H_b(\boldsymbol{r}_c))^2]^{1/2}$. [d] $R = [(G_b(\boldsymbol{r}_c) + V_b(\boldsymbol{r}_c)/2)^2 + (G_b(\boldsymbol{r}_c) + V_b(\boldsymbol{r}_c))^2]^{1/2}$.

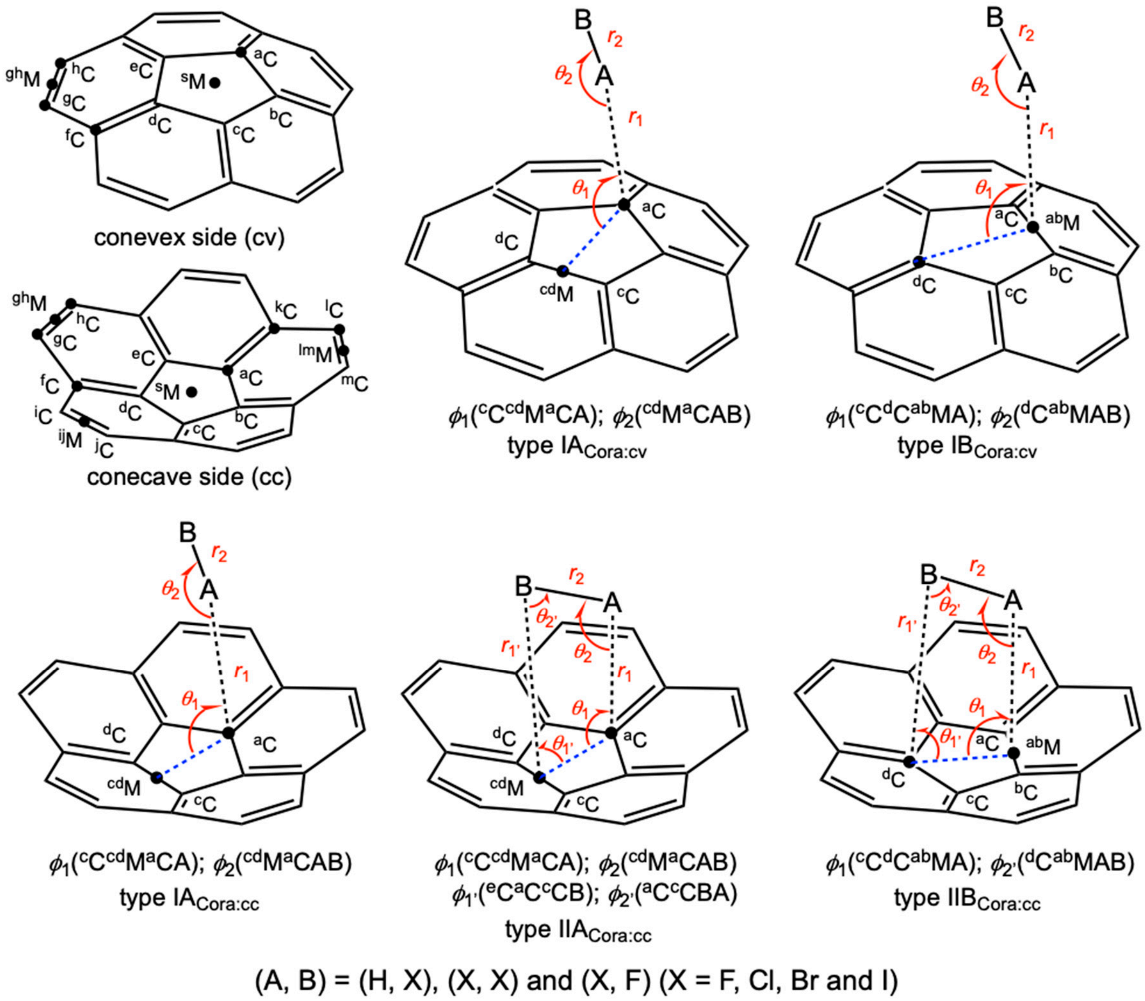

Scheme S4. Convex (cv) and concave (cc) sides of corannulene and definition of structural types of B–A---π($C_{20}H_{10}$), to be clarified, where (A, B) = (H, X), (X, X), or (X, F) (X = F, Cl, Br and I) with structural parameters.

Table S2. The structural parameters for X–H-∗-π($C_{20}H_{10}$) and Y–X-∗-π($C_{20}H_{10}$) (X, Y = F, Cl, Br and I), evaluated with MP2/BSS-A[a,b]

species	r_1 (Å)	r_2 (Å)	θ_1 (°)	θ_2 (°)	ϕ_1 (°)	ϕ_2 (°)
Convex side						
F–H-∗-π(^{a}C) (C_1: $IA_{Cora:cv}$)	2.1577	0.9278	106.1	174.7	-107.92	153.65
Cl–H-∗-π(^{ab}M) (C_s: $IB_{Cora:cv}$)	2.2123	1.2864	120.8	172.6	-90.08	177.87
Br–H-∗-π(^{ab}M) (C_1: $IB_{Cora:cv}$)	2.2059	1.4259	120.3	171.1	-89.86	-179.80
I–H-∗-π(^{ab}M) (C_s: $IB_{Cora:cv}$)	2.2118	1.6300	120.8	172.6	-89.87	180.00
F–F-∗-π(^{a}C) (C_s: $IA_{Cora:cv}$)	2.5216	1.4178	108.4	177.3	-90.00	0.00
Cl–Cl-∗-π(^{a}C) (C_s: $IA_{Cora:cv}$)	2.6865	2.0125	105.6	178.4	-90.00	0.00
Br–Br-∗-π(^{a}C) (C_s: $IA_{Cora:cv}$)	2.7162	2.3079	105.8	178.3	-90.00	0.00
I–I-∗-π(^{a}C) (C_s: $IA_{Cora:cv}$)	2.8729	2.6935	102.5	179.2	-90.00	0.00
F–Cl-∗-π(^{a}C) (C_s: $IA_{Cora:cv}$)	2.5441	1.6757	108.9	177.2	-90.00	0.00
F–Br-∗-π(^{a}C) (C_s: $IA_{Cora:cv}$)	2.5710	1.8145	108.8	177.3	-90.00	0.00
F–I-∗-π(^{a}C) (C_1: $IA_{Cora:cv}$)	2.7019	1.9583	106.5	106.5	-90.00	0.00
Concave side						
F–H-∗-π(^{a}C) (C_1: $IA_{Cora:cc}$)	2.2571	0.9263	75.0	167.8	-90.28	-1.08
Cl–H-∗-π(^{a}C) (C_s: $IIA_{Cora:cc}$)	2.3179	1.2859	82.4	141.3	-90.00	0.00
H–Cl-∗-π(^{c}C) (C_s: $IIA_{Cora:cc}$)[c,d]	3.3969	1.2859	70.9	63.3	-83.25	-8.97
Br–H-∗-π(^{a}C) (C_1: $IIA_{Cora:cc}$)	2.3134	1.4253	83.7	140.5	-90.00	0.00
H–Br-∗-π(^{c}C) (C_1: $IIA_{Cora:cc}$)[c,d]	3.4851	1.4253	72.1	61.8	-83.22	-8.96
I–H-∗-π(^{f}C) (C_1: $IIA_{Cora:cc}$)	2.4148	1.6163	88.1	131.7	-90.00	0.01
I–H-∗-π(^{c}C) (C_1: $IIA_{Cora:cc}$)[c,d]	3.6219	1.6163	73.5	64.6	-83.17	-10.21
F–F-∗-π(^{a}C) (C_s: $IIA_{Cora:cc}$)	2.9638	1.4045	78.7	104.6	-90.00	0.00
F–F-∗-π(^{c}C) (C_s: $IIA_{Cora:cc}$)[c,d]	3.0747	1.4045	82.5	90.4	-78.29	-16.90
Cl–Cl-∗-π(^{f}C) (C_1: $IIA_{Cora:cc}$)	3.2393	1.9909	72.7	99.4	-90.00	0.00
Cl–Cl-∗-π(^{ij}M) (C_1: $IIA_{Cora:cc}$)	3.2877	1.9909	60.2	125.7	-87.85	-12.25
Br–Br-∗-π(^{a}C) (C_s: $IIB_{Cora:cc}$)	3.1310	2.2747	76.8	136.4	-90.18	0.00
Br–Br-∗-π(^{i}C) (C_s: $IIB_{Cora:cc}$)[c,d]	3.8110	2.2747	77.0	79.1	-106.96	10.37
I–I-∗-π(^{a}C) (C_s: $IIB_{Cora:cc}$)	3.2751	2.6589	77.0	133.9	-90.26	0.00
I–I-∗-π(^{d}C) (C_s: $IIB_{Cora:cc}$)[c,d]	4.6346	2.6589	100.5	48.6	-83.78	0.00
I–I-∗-π(^{g}C) (C_s: $IIB_{Cora:cc}$)[c,d]	3.9223	2.6589	83.8	74.4	-108.28	11.02
F–Cl-∗-π(^{a}C) (C_1: $IA_{Cora:cc}$)	2.9621	1.6482	73.4	177.0	-90.00	0.04
F–Br-∗-π(^{a}C) (C_1: $IA_{Cora:cc}$)	3.0397	1.7818	73.9	177.4	-90.00	0.08
F–I-∗-π(^{a}C) (C_s: $IA_{Cora:cc}$)	3.1824	1.9358	73.8	178.9	-90.00	-180.00

[a] See text for BSS-A. [b] See Scheme S1 for the definition of the structural parameters. [c] Data shown in italic correspond to the additional interaction. [d] The descriptions of r_1, r_2, θ_1, θ_2, ϕ_1 and ϕ_2 should be read as $r_{1'}$, $r_{2'}$, $\theta_{1'}$, $\theta_{2'}$, $\phi_{1'}$ and $\phi_{2'}$, respectively.

Table S3. ΔE_{ES} and ΔE_{ZP} in X–H-*-π($C_{20}H_{10}$) and Y–X-*-π($C_{20}H_{10}$) (X, Y = F, Cl, Br and I), evaluated with MP2/BSS-A, together with second-perturbation energies $E(2)$ for the donor-acceptor (NBO(i)→NBO(j)) interactions of π(C=C)→σ*(X–H) and π(C=C)→σ*(Y–X), calculated with the NBO analysis under M06-2X/BSS-A//MP2/BSS-A[a]

species	ΔE_{ES}[b,c] (kJ mol^{-1})	ΔE_{ZP}[b,d] (kJ mol^{-1})	$E(2)$[e] (kcal mol^{-1})	$\varepsilon_j - \varepsilon_i$[f] (au)	$F(i,j)$[g] (au)
Convex side					
F–H-*-π(^{a}C) (C_1: IA$_{Cora:cv}$)	-23.4	-19.1	2.54	0.82	0.041
Cl–H-*-π(^{ab}M) (C_s: IB$_{Cora:cv}$)	-25.9	-23.3	1.25^{h}	0.56	0.024
Br–H-*-π(^{ab}M) (C_1: IB$_{Cora:cv}$)	-27.8	-25.6	1.58^{h}	0.48	0.025
I–H-*-π(^{ab}M) (C_s: IB$_{Cora:cv}$)	-28.0	-25.5	1.84^{h}	0.43	0.025
F–F-*-π(^{a}C) (C_s: IA$_{Cora:cv}$)	-17.0	-15.8	4.47	0.27	0.031
Cl–Cl-*-π(^{a}C) (C_s: IA$_{Cora:cv}$)	-36.6	-35.2	6.78	0.32	0.042
Br–Br-*-π(^{a}C) (C_s: IA$_{Cora:cv}$)	-43.0	-41.7	10.45	0.28	0.048
I–I-*-π(^{a}C) (C_s: IA$_{Cora:cv}$)	-48.6	-47.5	9.00	0.27	0.044
F–Cl-*-π(^{a}C) (C_s: IA$_{Cora:cv}$)	-41.4	-39.2	13.23	0.34	0.060
F–Br-*-π(^{a}C) (C_s: IA$_{Cora:cv}$)	-53.2	-50.9	19.21	0.33	0.071
F–I-*-π(^{a}C) (C_1: IA$_{Cora:cv}$)	-64.3	-61.9	45.02^{i}	0.18	0.081
Concave side					
F–H-*-π(^{a}C) (C_1: IA$_{Cora:cc}$)	-27.8	-24.8	1.16	0.81	0.027
Cl–H-*-π(^{a}C) (C_s: IIA$_{Cora:cc}$)	-44.7	-42.0	2.77	0.55	0.035
Br–H-*-π(^{a}C) (C_1: IIA$_{Cora:cc}$)	-49.2	-46.7	3.73	0.49	0.038
I–H-*-π(^{f}C) (C_1: IIA$_{Cora:cc}$)	-12.3	-10.0	3.52	0.43	0.035
F–F-*-π(^{a}C) (C_s: IIA$_{Cora:cc}$)	-28.5	-26.7	j	j	j
Cl–Cl-*-π(^{f}C) (C_1: IIA$_{Cora:cc}$)	-58.6	-56.9	0.71	0.37	0.015
Br–Br-*-π(^{a}C) (C_s: IIB$_{Cora:cc}$)	-62.8	-61.4	0.93^{h}	0.28	0.014
I–I-*-π(^{a}C) (C_s: IIB$_{Cora:cc}$)	-70.9	-69.9	0.98^{h,k}	0.26	0.014
F–Cl-*-π(^{a}C) (C_1: IA$_{Cora:cc}$)	-48.5	-46.8	2.56	0.35	0.027
F–Br-*-π(^{a}C) (C_1: IA$_{Cora:cc}$)	-55.1	-53.6	3.18	0.32	0.028
F–I-*-π(^{a}C) (C_s: IA$_{Cora:cc}$)	-63.2	-62.1	2.69	0.33	0.026
Convex side					
F–H-*-π(^{s}M) (C_{5v}: ID$_{Cora:cv}$)^{l}	-20.3	-19.1	0.20^{h}	1.25	0.014
Cl–H-*-π(^{s}M) (C_{5v}: ID$_{Cora:cv}$)^{l}	-24.4	-23.7	0.09^{h}	0.55	0.006
Br–H-*-π(^{s}M) (C_{5v}: ID$_{Cora:cv}$)^{l}	-25.8	-25.2	0.12^{h}	0.48	0.007
I–H-*-π(^{s}M) (C_{5v}: ID$_{Cora:cv}$)^{l}	-27.0	-26.4	1.49^{h,i}	0.25	0.017
F–F-*-π(^{s}M) (C_{5v}: ID$_{Cora:cv}$)^{l}	-14.6	-13.8	0.06^{h}	0.27	0.004
Cl–Cl-*-π(^{s}M) (C_{5v}: ID$_{Cora:cv}$)^{l}	-30.5	-29.7	1.42^{h,i}	0.14	0.012
Br–Br-*-π(^{s}M) (C_{5v}: ID$_{Cora:cv}$)^{l}	-33.8	-33.1	2.49^{h,i}	0.09	0.013
I–I-*-π(^{s}M) (C_{5v}: ID$_{Cora:cv}$)^{l}	-40.6	-40.0	3.31^{h,i}	0.07	0.014
F–Cl-*-π(^{s}M) (C_{5v}: ID$_{Cora:cv}$)^{l}	-27.9	-26.8	1.35^{h,i}	0.16	0.013
F–Br-*-π(^{s}M) (C_{5v}: ID$_{Cora:cv}$)^{l}	-33.4	-32.3	2.15^{h,i}	0.13	0.015
F–I-*-π(^{s}M) (C_{5v}: ID$_{Cora:cv}$)^{l}	-62.8	-62.1	0.76^{h}	0.32	0.014
Concave side					
F–H-*-π(^{s}M) (C_{5v}: ID$_{Cora:cc}$)^{l}	-26.8	-26.1	0.23^{h}	1.24	0.015
Cl–H-*-π(^{s}M) (C_{5v}: ID$_{Cora:cc}$)^{l}	-42.1	-41.2	0.31^{h}	0.55	0.012
Br–H-*-π(^{s}M) (C_{5v}: ID$_{Cora:cc}$)^{l}	-46.0	-45.4	0.47^{h}	0.48	0.013
I–H-*-π(^{s}M) (C_{5v}: ID$_{Cora:cc}$)^{l}	-8.4	-7.6	0.60^{h}	0.43	0.014
F–F-*-π(^{s}M) (C_{5v}: ID$_{Cora:cc}$)^{l}	-25.3	-24.5	0.92^{h,i}	0.09	0.008
Cl–Cl-*-π(^{s}M) (C_{5v}: ID$_{Cora:cc}$)^{l}	-54.9	-53.8	0.45^{h}	0.32	0.011
Br–Br-*-π(^{s}M) (C_{5v}: ID$_{Cora:cc}$)^{l}	-59.1	-58.2	0.63^{h}	0.28	0.012
I–I-*-π(^{s}M) (C_{5v}: ID$_{Cora:cc}$)^{l}	-66.3	-65.7	0.78^{h}	0.26	0.013
F–Cl-*-π(^{s}M) (C_{5v}: ID$_{Cora:cc}$)^{l}	-47.8	-46.6	1.35^{h,i}	0.16	0.013
F–Br-*-π(^{s}M) (C_{5v}: ID$_{Cora:cc}$)^{l}	-54.4	-53.3	0.68^{h}	0.31	0.013
F–I-*-π(^{s}M) (C_{5v}: ID$_{Cora:cc}$)^{l}	-43.1	-41.8	2.56^{h,i}	0.14	0.017

[a] See text for BSS-A. [b] $\Delta E = E$(X–H---π($C_{24}H_{12}$)/Y–X---π($C_{24}H_{12}$)) – (E(X–H/Y–X) + E($C_{24}H_{12}$)). [c] ΔE_{ES} stands for ΔE on the energy surface. [d] ΔE_{Ent} (ΔE_{ZP}) stands for ΔE with the correction of the heat of enthalpy. [e] Second-perturbation energy. [f] The diagonal elements (orbital energies). [g] The off-diagonal NBO Fock matrix element. [h] Only one side of the CT interaction is shown due to symmetry. [i] NBO (i) is n_p(C) which constitutes π(C=C). [j] Not detected. [k] Non negligible $E(2)$ value for 0.66 kcal mol^{-1} relative to 0.98 kcal mol^{-1} is also detected. [l] Two imaginary frequencies being predicted for each.

Table S4. The r_{BP} and R_{SL} values evaluated with MP2/BSS-A for the optimized and observed structures of B–A-∗-π($C_{20}H_{10}$), together with the Δr_{BP} values^{a}

Compound	r_{BP}^{b} (Å)	R_{SL}^{c} (Å)	Δr_{BP}^{d} (Å)
Convex side			
F–H-∗-π(^{a}C) (C_1: $IA_{Cora:cv}$)	2.1958	2.1577	0.038
Cl–H-∗-π(^{ab}M) (C_s: $IB_{Cora:cv}$)	2.2157	2.2118	0.004
Br–H-∗-π(^{ab}M) (C_1: $IB_{Cora:cv}$)	2.8865	2.2059	0.681
I–H-∗-π(^{ab}M) (C_s: $IB_{Cora:cv}$)	2.2101	2.1984	0.012
F–F-∗-π(^{a}C) (C_s: $IA_{Cora:cv}$)	2.5238	2.5216	0.002
Cl–Cl-∗-π(^{a}C) (C_s: $IA_{Cora:cv}$)	2.6869	2.6865	0.000
Br–Br-∗-π(^{a}C) (C_s: $IA_{Cora:cv}$)	2.7165	2.7162	0.000
I–I-∗-π(^{a}C) (C_s: $IA_{Cora:cv}$)	2.8730	2.8729	0.000
F–Cl-∗-π(^{a}C) (C_s: $IA_{Cora:cv}$)	2.5458	2.5441	0.002
F–Br-∗-π(^{a}C) (C_s: $IA_{Cora:cv}$)	2.5720	2.5710	0.001
F–I-∗-π(^{a}C) (C_1: $IA_{Cora:cv}$)	2.7022	2.7019	0.000
Concave side			
F–H-∗-π(^{a}C) (C_1: $IA_{Cora:cc}$)	2.2862	2.2571	0.029
Cl–H-∗-π(^{a}C) (C_s: $IIA_{Cora:cc}$)	2.4148	2.3179	0.097
H–Cl -∗-π(^{d}C) (C_s: $IIA_{Cora:cc}$)^{d}	*2.4148*	*2.3179*	*0.097*
Br–H-∗-π(^{a}C) (C_1: $IIA_{Cora:cc}$)	2.4934	2.3134	0.180
H–Br-∗-π(^{d}C) (C_1: $IIA_{Cora:cc}$)^{d}	*3.4962*	*3.4851*	*0.011*
I–H-∗-π(^{f}C) (C_1: $IIA_{Cora:cc}$)	2.4387	2.4148	0.024
H–I-∗-π(^{d}C) (C_1: $IIA_{Cora:cc}$)^{d}	*2.4387*	*2.4148*	*0.024*
F–F-∗-π(^{a}C) (C_s: $IIA_{Cora:cc}$)	3.0965	3.0747	0.022
F–F-∗-π(^{d}C) (C_s: $IIA_{Cora:cc}$)^{d}	*3.0009*	*2.9638*	*0.037*
Cl–Cl-∗-π(^{f}C) (C_1: $IIA_{Cora:cc}$)	3.1816	3.1218	0.060
Cl–Cl-∗-π(^{ij}M) (C_1: $IIA_{Cora:cc}$)^{d}	*3.9133*	*3.2127*	*0.701*
Br–Br-∗-π(^{a}C) (C_s: $IIB_{Cora:cc}$)	3.2291	3.2100	0.019
Br–Br-∗-π(^{f}C) (C_s: $IIB_{Cora:cc}$)^{d}	*3.8225*	*3.8078*	*0.015*
Br–Br-∗-π(^{g}C) (C_s: $IIB_{Cora:cc}$)^{d}	*3.8225*	*3.8078*	*0.015*
I–I-∗-π(^{a}C) (C_s: $IIB_{Cora:cc}$)	3.3618	3.3507	0.011
I–I-∗-π(^{f}C) (C_s: $IIB_{Cora:cc}$)^{d}	*3.9632*	*3.9223*	*0.041*
I–I-∗-π(^{g}C) (C_s: $IIB_{Cora:cc}$)^{d}	*3.9632*	*3.9223*	*0.041*
F–Cl-∗-π(^{a}C) (C_1: $IA_{Cora:cc}$)	2.9661	2.9621	0.004
F–Br-∗-π(^{a}C) (C_1: $IA_{Cora:cc}$)	3.0416	3.0397	0.002
F–I-∗-π(^{a}C) (C_s: $IA_{Cora:cc}$)	3.1833	3.1824	0.001

^{a} See text for BSS-A. ^{b} The lengths of BPs. ^{c} Straight-line distances. ^{d} $\Delta r_{BP} = r_{BP} - R_{SL}$. ^{d} Data shown in italic correspond to the additional interaction.

Table S5. The r_{BP} and R_{SL} values evaluated with MP2/BSS-A for the optimized and observed structures of B–A-∗-π($C_{20}H_{10}$), together with the Δr_{BP} values[a]

Compound	r_{BP}[b] (Å)	R_{SL}[c] (Å)	Δr_{BP}[d] (Å)
Convex side			
F–H-∗-π(^{a}C) (C_{5v}: $ID_{Cora:cv}$)	2.6264	2.5708	0.056
Cl–H-∗-π(^{a}C) (C_{5v}: $ID_{Cora:cv}$)	2.5863	2.5469	0.039
Br–H-∗-π(^{a}C) (C_{5v}: $ID_{Cora:cv}$)	2.5794	2.5436	0.036
I–H-∗-π(^{a}C) (C_{5v}: $ID_{Cora:cv}$)	2.5664	2.5353	0.031
F–F-∗-π(^{a}C) (C_{5v}: $ID_{Cora:cv}$)	3.0443	3.0163	0.028
Cl–Cl-∗-π(^{a}C) (C_{5v}: $ID_{Cora:cv}$)	3.2257	3.2004	0.025
Br–Br-∗-π(^{a}C) (C_{5v}: $ID_{Cora:cv}$)	3.2977	3.2801	0.018
I–I-∗-π(^{a}C) (C_{5v}: $ID_{Cora:cv}$)	3.3932	3.3775	0.016
F–Cl-∗-π(^{a}C) (C_{5v}: $ID_{Cora:cv}$)	3.2552	3.2162	0.039
F–Br-∗-π(^{a}C) (C_{5v}: $ID_{Cora:cv}$)	3.2846	3.2595	0.025
F–I-∗-π(^{a}C) (C_{5v}: $ID_{Cora:cv}$)	3.3401	3.3178	0.022
Concave side			
F–H-∗-π(^{a}C) (C_{5v}: $ID_{Cora:cc}$)	2.5357	2.4590	0.077
Cl–H-∗-π(^{a}C) (C_{5v}: $ID_{Cora:cc}$)	2.4370	2.3858	0.051
Br–H-∗-π(^{a}C) (C_{5v}: $ID_{Cora:cc}$)	2.4274	2.3802	0.047
I–H-∗-π(^{a}C) (C_{5v}: $ID_{Cora:cc}$)	2.4180	2.3786	0.039
F–F-∗-π(^{a}C) (C_{5v}: $ID_{Cora:cc}$)	2.9206	2.8966	0.024
Cl–Cl-∗-π(^{a}C) (C_{5v}: $ID_{Cora:cc}$)	3.1061	3.0880	0.018
Br–Br-∗-π(^{a}C) (C_{5v}: I $ID_{Cora:cc}$)	3.1956	3.1861	0.010
I–I-∗-π(^{a}C) (C_{5v}: $ID_{Cora:cc}$)	3.3271	3.3205	0.007
F–Cl-∗-π(^{a}C) (C_{5v}: $ID_{Cora:cc}$)	3.1291	3.0974	0.032
F–Br-∗-π(^{a}C) (C_{5v}: $ID_{Cora:cc}$)	3.1884	3.1713	0.017
F–I-∗-π(^{a}C) (C_{5v}: $ID_{Cora:cc}$)	3.2987	3.2867	0.012

[a] See text for BSS-A. [b] The lengths of BPs. [c] Straight-line distances. [d] $\Delta r_{BP} = r_{BP} - R_{SL}$.

Table S6. The structural parameters for the H-*-π and X-*-π interactions with π($C_{20}H_{10}$), π($C_{24}H_{12}$) and π(C_6H_6), (X = F, Cl, Br and I), evaluated with MP2/BSS-A,[a] together with the values from the corresponding ones of the adducts with π(C_6H_6), respectively

species	r_1 (Å)	r_2 (Å)	θ_1 (°)	θ_2 (°)	ϕ_1 (°)	ϕ_2 (°)
Convex side						
F–H-*-π(^{s}M) (C_{5v}: $ID_{Cora:cv}$)[b]	2.2724	0.9241	90.00	180.00	-90.00	83.12
Cl–H-*-π(^{s}M) (C_{5v}: $ID_{Cora:cv}$)[b]	2.2455	1.2818	90.00	180.00	-90.00	162.89
Br–H-*-π(^{s}M) (C_{5v}: $ID_{Cora:cv}$)[b]	2.2418	1.4199	90.00	180.00	-90.00	-93.61
I–H-*-π(^{s}M) (C_{5v}: $ID_{Cora:cv}$)[b]	2.2324	1.6142	90.00	180.00	-90.00	135.83
F–F-*-π(^{s}M) (C_{5v}: $ID_{Cora:cv}$)[b]	2.7666	1.4031	90.00	180.00	-90.00	88.63
Cl–Cl-*-π(^{s}M) (C_{5v}: $ID_{Cora:cv}$)[b]	2.9662	1.9921	90.00	180.00	-90.00	-102.14
Br–Br-*-π(^{s}M) (C_{5v}: $ID_{Cora:cv}$)[b]	3.0521	2.2791	90.00	180.00	-90.00	-42.80
I–I-*-π(^{s}M) (C_{5v}: $ID_{Cora:cv}$)[b]	3.1566	2.6678	90.00	180.00	-90.00	-65.30
F–Cl-*-π(^{s}M) (C_{5v}: $ID_{Cora:cv}$)[b]	2.9832	1.6442	90.00	180.00	-90.00	83.81
F–Br-*-π(^{s}M) (C_{5v}: $ID_{Cora:cv}$)[b]	3.0299	1.7783	90.00	180.00	-90.00	141.67
F–I-*-π(^{s}M) (C_{5v}: $ID_{Cora:cv}$)[b]	3.0925	1.9340	90.00	180.00	-90.00	119.25
Concave side						
F–H-*-π(^{s}M) (C_{5v}: $ID_{Cora:cc}$)[b]	2.1444	0.9249	90.00	180.00	90.00	-121.02
Cl–H-*-π(^{s}M) (C_{5v}: $ID_{Cora:cc}$)[b]	2.0599	1.2835	90.00	180.00	90.00	-134.23
Br–H-*-π(^{s}M) (C_{5v}: $ID_{Cora:cc}$)[b]	2.0534	1.4216	90.00	180.00	90.00	145.32
I–H-*-π(^{s}M) (C_{5v}: $ID_{Cora:cc}$)[b]	2.0514	1.6121	90.00	180.00	90.00	61.10
F–F-*-π(^{s}M) (C_{5v}: $ID_{Cora:cc}$)[b]	2.6351	1.4038	90.00	180.00	90.00	-81.04
Cl–Cl-*-π(^{s}M) (C_{5v}: $ID_{Cora:cc}$)[b]	2.8438	1.9914	90.00	180.00	90.00	114.89
Br–Br-*-π(^{s}M) (C_{5v}: $ID_{Cora:cc}$)[b]	2.9501	2.2781	90.00	180.00	90.00	-36.00
I–I-*-π(^{s}M) (C_{5v}: $ID_{Cora:cc}$)[b]	3.0951	2.6650	90.00	180.00	90.00	-48.94
F–Cl-*-π(^{s}M) (C_{5v}: $ID_{Cora:cc}$)[b]	2.8541	1.6461	90.00	180.00	90.00	-77.74
F–Br-*-π(^{s}M) (C_{5v}: $ID_{Cora:cc}$)[b]	2.9342	1.7798	90.00	180.00	90.00	-135.79
F–I-*-π(^{s}M) (C_{5v}: $ID_{Cora:cc}$)[b]	3.0588	1.9348	90.00	180.00	90.00	77.56
Y–X-*-π($C_{24}H_{12}$)						
F–H-*-π(M_o) (C_{6v}: ID_{Cor})[b]	2.2261	0.9239	90.00	180.00	-90.00	-23.31
Cl–H-*-π(M_o) (C_{6v}: ID_{Cor})[b]	2.1648	1.2815	90.00	180.00	-90.00	141.49
Br–H-*-π(M_o) (C_{6v}: ID_{Cor})[b]	2.1555	1.4197	90.00	180.00	-90.00	-180.00
I–H-*-π(M_o) (C_{6v}: ID_{Cor})[b]	2.1348	1.6139	90.00	180.00	-90.00	87.85
F–F-*-π(M_o) (C_{6v}: ID_{Cor})[b]	2.7234	1.4027	90.00	180.00	-90.00	150.12
Cl–Cl-*-p(M_o) (C_{6v}: ID_{Cor})[b]	2.9193	1.9913	90.00	180.00	-90.00	93.40
Br–Br-*-π(M_o) (C_{6v}: ID_{Cor})[b]	3.0091	2.2781	90.00	180.00	-90.00	180.00
I–I-*-π(M_o) (C_{6v}: ID_{Cor})[b]	3.1252	2.6662	90.00	180.00	-90.00	0.00
F–Cl-*-π(M_o) (C_{6v}: ID_{Cor})[b]	2.9516	1.6436	90.00	180.00	-90.00	-94.92
F–Br-*-π(M_o) (C_{6v}: ID_{Cor})[b]	3.0072	1.7776	90.00	180.00	-90.00	105.76
F–I-*-π(M_o) (C_{6v}: ID_{Cor})[b]	3.0898	1.9332	90.00	180.00	-90.00	180.00
Y–X-*-π(C_6H_6)						
F–H-*-π(M_o) (C_{6v}: ID_{Bzn})[b]	2.2223	0.9250	90.00	180.00	90.00	-69.61
Cl–H-*-π(M_o) (C_{6v}: ID_{Bzn})[b]	2.2422	1.2826	90.00	180.00	90.00	-52.40
Br–H-*-π(M_o) (C_{6v}: ID_{Bzn})[b]	2.2605	1.4206	90.00	180.00	90.00	-171.08
I–H-*-π(M_o) (C_{6v}: ID_{Bzn})[b]	2.2749	1.6151	90.00	180.00	90.00	119.98
F–F-*-π(M_o) (C_{6v}: ID_{Bzn})[b]	2.9041	1.4013	90.00	180.00	90.00	-68.01
Cl–Cl-*-π(M_o) (C_{6v}: ID_{Bzn})[b]	3.1058	1.9901	90.00	180.00	90.00	55.03
Br–Br-*-π(M_o) (C_{6v}: ID_{Bzn})[b]	3.2256	2.2754	90.00	180.00	90.00	104.17
I–I-*-π(M_o) (C_{6v}: ID_{Bzn})[b]	3.3740	2.6623	90.00	180.00	90.00	-129.82
F–Cl-*-π(M_o) (C_{6v}: ID_{Bzn})[b]	3.1210	1.6420	90.00	180.00	90.00	49.37
F–Br-*-π(M_o) (C_{6v}: ID_{Bzn})[b]	3.2037	1.7748	90.00	180.00	90.00	53.37
F–I-*-π(M_o) (C_{6v}: ID_{Bzn})[b]	3.3184	1.9301	90.00	180.00	90.00	-130.35

[a] See text for BSS-A. [b] Two imaginary frequencies being predicted for each.

Table S7. QTAIM functions and QTAIM-DFA parameters for X–H-*-$\pi(C_{20}H_{10})$ and Y–X-*-$\pi(C_{20}H_{10})$ (X, Y = F, Cl, Br, and I) on concave side, evaluated with MP2/BSS-A[1,2] with NIV

**Y–X-*-$\pi(C_{20}H_{10})$** (symmetry: type)	$\rho_b(r_c)$ (ea_o^{-3})	$c\nabla^2\rho_b(r_c)$[3] (au)	$H_b(r_c)$ (au)	R[4] (au)	θ[5] (°)	**freq** (cm^{-1})	θ_p[6] (°)	κ_p[7] (au^{-1})	**Predicted nature**
Concave side									
F–H-*-$\pi(^aC)$ (C_1: $IA_{Cora:cc}$)	0.0144	0.0065	0.0021	0.0068	72.0	100.0	100.2	282.2	*p*-CS/*t*-HB_{nc}
Cl–H-*-$\pi(^aC)$ (C_s: $IIA_{Cora:cc}$)	0.0162	0.0064	0.0015	0.0065	76.9	77.7	125.4	955.4	*p*-CS/*t*-HB_{nc}
H–Cl--$\pi(^cC)$ (C_s: $IIA_{Cora:cc}$)*[9]	*0.0083*	*0.0038*	*0.0013*	*0.0040*	*71.1*	*77.7*	*89.9*	*4888*	*p-CS/vdw*
Br–H-*-$\pi(^aC)$ (C_1: $IIA_{Cora:cc}$)	0.0174	0.0065	0.0014	0.0066	78.2	59.0	137.7	1830	*p*-CS/*t*-HB_{nc}
H–Br--$\pi(^cC)$ (C_1: $IIA_{Cora:cc}$)*[9]	*0.0082*	*0.0036*	*0.0012*	*0.0038*	*71.6*	*59.0*	*101.4*	*10580*	*p-CS/t-HB_{nc}*
I–H-*-$\pi(^fC)$ (C_1: $IIA_{Cora:cc}$)	0.0172	0.0062	0.0012	0.0063	78.9	53.5	92.2	344.1	*p*-CS/*t*-HB_{nc}
I–H--$\pi(^cC)$ (C_1: $IIA_{Cora:cc}$)*[9]	*0.0086*	*0.0031*	*0.0008*	*0.0032*	*75.1*	*53.5*	*85.0*	*8.0*	*p-CS/vdw*
F–F-*-$\pi(^aC)$ (C_s: $IIA_{Cora:cc}$)	0.0093	0.0048	0.0012	0.0050	76.3	76.2	89.3	305.2	*p*-CS/vdw
F–F--$\pi(^cC)$ (C_s: $IIA_{Cora:cc}$)*[9]	*0.0077*	*0.0041*	*0.0011*	*0.0043*	*74.9*	*76.2*	*83.3*	*7.4*	*p-CS/vdw*
Cl–Cl-*-$\pi(^fC)$ (C_1: $IIA_{Cora:cc}$)	0.0118	0.0054	0.0017	0.0056	72.8	97.0	84.5	94.5	*p*-CS/vdw
Cl–Cl--$\pi(^{ij}M)$ (C_1: $IIA_{Cora:cc}$)*[9]	*0.0099*	*0.0042*	*0.0015*	*0.0045*	*71.0*	*95.4*	*76.5*	*69.3*	*p-CS/vdw*
Br–Br-*-$\pi(^aC)$ (C_s: $IIB_{Cora:cc}$)	0.0124	0.0054	0.0014	0.0056	75.4	76.0	88.5	98.1	*p*-CS/vdw
Br–Br--$\pi(^iC)$ (C_s: $IIB_{Cora:cc}$)*[9]	*0.0046*	*0.0018*	*0.0007*	*0.0019*	*68.8*	*44.8*	*71.5*	*37.6*	*p-CS/vdw*
I–I-*-$\pi(^aC)$ (C_s: $IIB_{Cora:cc}$)	0.0130	0.0049	0.0010	0.0050	78.7	70.9	94.1	136.2	*p*-CS/*t*-HB_{nc}
I–I--$\pi(^dC)$ (C_s: $IIB_{Cora:cc}$)*[9]	*0.0111*	*0.0045*	*0.0011*	*0.0046*	*76.5*	*70.9*	*87.7*	*102.1*	*p-CS/vdw*
I–I--$\pi(^gC)$ (C_s: $IIB_{Cora:cc}$)*[9]	*0.0050*	*0.0017*	*0.0006*	*0.0018*	*71.0*	*44.7*	*75.6*	*58.4*	*p-CS/vdw*
F–Cl-*-$\pi(^aC)$ (C_1: $IA_{Cora:cc}$)	0.0137	0.0065	0.0017	0.0067	75.6	95.4	93.8	131.4	*p*-CS/*t*-HB_{nc}
F–Br-*-$\pi(^aC)$ (C_1: $IA_{Cora:cc}$)	0.0139	0.0061	0.0014	0.0062	76.9	79.2	97.1	168.1	*p*-CS/*t*-HB_{nc}
F–I-*-$\pi(^aC)$ (C_s: $IA_{Cora:cc}$)	0.0141	0.0054	0.0008	0.0054	81.1	76.0	107.7	228.9	*p*-CS/*t*-HB_{nc}

[1] See text for BSS-A. [2] Data are given at BCP, which is shown by A-*-π, where one side interaction is shown if two are identical due to symmetry. [3] $c\nabla^2\rho_b(r_c) = H_b(r_c) - V_b(r_c)/2$, where $c = \hbar^2/8m$. [4] $R = (x^2 + y^2)^{1/2}$, where $(x, y) = (H_b(r_c) - V_b(r_c)/2, H_b(r_c))$. [5] $\theta = 90° - \tan^{-1}(y/x)$. [6] $\theta_p = 90° - \tan^{-1}(dy/dx)$. [7] $\kappa_p = |d^2y/dx^2|/[1 + (dy/dx)^2]^{3/2}$. [8] Perturbed structures are generated employing w = –0.05, –0.025, (0), 0.025, and 0.05 in Equation (4) of the text. [9] Data shown in italics correspond to the additional interaction.

The nature of the additional interactions on the concave side, given in Table S7 in italics, are similarly calculated. The additional interactions are weaker than the corresponding original interactions based on the QTAIM-DFA parameters listed in Table S7. The predicted nature for the additional interactions are weaker than that for the original one for H–Cl-*-$\pi(^cC)$ versus Cl–H-*-$\pi(^aC)$ (C_s: $IIA_{Cora:cc}$) and H–I-*-$\pi(^cC)$ versus I–H-*-$\pi(^fC)$ (C_s: $IIA_{Cora:cc}$), while the same nature is predicted for H–Br-*-$\pi(^cC)$ versus Br–H-*-$\pi(^aC)$ (C_s: $IIA_{Cora:cc}$), F–F'-*-$\pi(^cC)$ versus F'–F-*-$\pi(^aC)$ (C_s: $IIA_{Cora:cc}$), Br–Br'-*-$\pi(^iC)$ versus Br'–Br-*-$\pi(^aC)$ (C_s: $IIA_{Cora:cc}$) and I–I'-*-$\pi(^dC)$ versus I–I'-*-$\pi(^gC)$ (The additional interactions seem to appear depending on the relative sizes of X–H, X–X, and cc of $\pi(C_{20}H_{10})$ in the adducts, together with the interaction ability of X.

Table S8. C_{ii} and ΔE for X–H-∗-π($C_{20}H_{10}$) and Y–X-∗-π($C_{20}H_{10}$) (X, Y = F, Cl, Br, and I), evaluated with MP2/BSS-A,[a] employing the perturbed structures generated with CIV

Compound	C_{ii} (Å mdyn^{-1})	$\Sigma_k C_{ii}{}^{-1}{}_k$ [b] (mdyn Å^{-1})	ΔE_{ES} (kJ mol^{-1})
Convex side (with CIV)			
F–H-∗-π(^{a}C) (C_1: $IA_{Cora:cv}$)	17.674	0.05658	-23.4
F–F-∗-π(^{a}C) (C_s: $IA_{Cora:cv}$)	9.004	0.111062	-17.0
Cl–Cl-∗-π(^{a}C) (C_s: $IA_{Cora:cv}$)	4.795	0.208551	-36.6
Br–Br-∗-π(^{a}C) (C_s: $IA_{Cora:cv}$)	4.197	0.238265	-43.0
I–I-∗-π(^{a}C) (C_s: $IA_{Cora:cv}$)	3.828	0.261233	-48.6
F–Cl-∗-π(^{a}C) (C_s: $IA_{Cora:cv}$)	4.321	0.231428	-41.4
F–Br-∗-π(^{a}C) (C_s: $IA_{Cora:cv}$)	3.036	0.329381	-53.2
F–I-∗-π(^{a}C) (C_1: $IA_{Cora:cv}$)	2.487	0.402091	-64.3
Concave side (with CIV)			
F–H-∗-π(^{a}C) (C_1: $IA_{Cora:cc}$)	8.175	0.122324	-27.8
Cl–H-∗-π(^{a}C) (C_s: $IIA_{Cora:cc}$)	31.667, 9.879, 9.879	0.234028	-44.7
Br–H-∗-π(^{a}C) (C_1: $IIA_{Cora:cc}$)	34.149, 8.662, 8.662	0.260177	-49.2
I–H-∗-π(^{f}C) (C_1: $IIA_{Cora:cc}$)	57.414, 8.001, 8.001	0.267386	-12.3
F–F-∗-π(^{a}C) (C_s: $IIA_{Cora:cc}$)	12.665, 18.106, 18.106	0.189418	-28.5
Br–Br-∗-π(^{a}C) (C_s: $IIB_{Cora:cc}$)	80.397, 80.397, 24.007, 9.442, 9.442	0.278351	-62.8
I–I-∗-π(^{a}C) (C_s: $IIB_{Cora:cc}$)	40.039, 40.039, 6.620, 4.975, 4.975	0.603019	-70.9
F–Cl-∗-π(^{a}C) (C_1: $IA_{Cora:cc}$)	6.162	0.162285	-48.5
F–Br-∗-π(^{a}C) (C_1: $IA_{Cora:cc}$)	5.875	0.170213	-55.1
F–I-∗-π(^{a}C) (C_s: $IA_{Cora:cc}$)	5.412	0.184775	-63.2

[a] See text for BSS-A. [b] Calculated according to Equation (18) in the text.

Table S9. QTAIM functions and QTAIM-DFA parameters for X–H-∗-π($C_{20}H_{10}$) and Y–X-∗-π($C_{20}H_{10}$) (X, Y = F, Cl, Br and I) (C_{5v}: $ID_{Cora:cv}$ and $ID_{Cora:cc}$), evaluated with MP2/BSS-A[a,b]

Y–X-∗-π($C_{20}H_{10}$) (symm: type)	$\rho_b(\boldsymbol{r}_c)$ (ea_o^{-3})	$c\nabla^2\rho_b(\boldsymbol{r}_c)^c$ (au)	$H_b(\boldsymbol{r}_c)$ (au)	R^d (au)	θ^e (°)	freq (cm^{-1})	$\theta_p{}^f$ (°)	$\kappa_p{}^g$ (au^{-1})	predicted nature
Convex side (C_{5v}: $ID_{Cora:cv}$)									
F–H-∗-π(^{a}C)^h	0.0079	0.0039	0.0017	0.0042	66.1	91.3	68.9	64.2	*p*-CS/vdW
Cl–H-∗-π(^{a}C)^h	0.0098	0.0044	0.0015	0.0046	71.5	78.9	74.4	57.0	*p*-CS/vdW
Br–H-∗-π(^{a}C)^h	0.0104	0.0045	0.0014	0.0047	72.5	58.6	75.8	57.5	*p*-CS/vdW
I–H-∗-π(^{a}C)^h	0.0111	0.0047	0.0014	0.0049	73.6	51.1	77.7	57.4	*p*-CS/vdW
F–F-∗-π(^{a}C)^h	0.0067	0.0038	0.0014	0.0041	70.5	70.6	73.5	9.8	*p*-CS/vdW
Cl–Cl-∗-π(^{a}C)^h	0.0090	0.0044	0.0017	0.0047	69.3	70.2	78.0	74.7	*p*-CS/vdW
Br–Br-∗-π(^{a}C)^h	0.0094	0.0042	0.0014	0.0044	71.0	54.0	80.6	88.6	*p*-CS/vdW
I–I-∗-π(^{a}C)^h	0.0107	0.0042	0.0011	0.0043	74.9	50.7	87.6	120.1	*p*-CS/vdW
F–Cl-∗-π(^{a}C)^h	0.0084	0.0041	0.0015	0.0044	69.6	74.1	77.6	75.5	*p*-CS/vdW
F–Br-∗-π(^{a}C)^h	0.0094	0.0041	0.0014	0.0044	71.7	62.6	81.3	95.0	*p*-CS/vdW
F–I-∗-π(^{a}C)^f	0.0113	0.0043	0.0010	0.0045	76.5	74.6	90.8	133.9	*p*-CS/*t*-HB_{nc}
Concave side (C_{5v}: $ID_{Cora:cc}$)									
F–H-∗-π(^{a}C)^h	0.0104	0.0054	0.0021	0.0058	68.2	100.4	76.0	91.4	*p*-CS/vdW
Cl–H-∗-π(^{a}C)^h	0.0143	0.0066	0.0018	0.0068	74.8	98.5	84.8	100.6	*p*-CS/vdW
Br–H-∗-π(^{a}C)^h	0.0151	0.0067	0.0017	0.0069	75.9	74.5	86.8	104.9	*p*-CS/vdW
I–H-∗-π(^{a}C)^h	0.0160	0.0069	0.0015	0.0071	77.8	66.1	88.1	100.0	*p*-CS/vdW
F–F-∗-π(^{a}C)^h	0.0090	0.0053	0.0017	0.0056	72.7	87.2	75.7	10.2	*p*-CS/vdW
Cl–Cl-∗-π(^{a}C)^h	0.0117	0.0059	0.0018	0.0062	72.9	88.7	85.5	61.8	*p*-CS/vdW
Br–Br-∗-π(^{a}C)^h	0.0117	0.0053	0.0015	0.0056	74.0	67.9	86.7	78.7	*p*-CS/vdW
I–I-∗-π(^{a}C)^h	0.0124	0.0049	0.0011	0.0050	77.6	63.1	93.4	134.8	*p*-CS/*t*-HB_{nc}
F–Cl-∗-π(^{a}C)^h	0.0112	0.0055	0.0017	0.0058	73.1	93.5	83.8	82.1	*p*-CS/vdW
F–Br-∗-π(^{a}C)^h	0.0115	0.0052	0.0014	0.0054	74.5	78.2	86.4	79.6	*p*-CS/vdW
F–I-∗-π(^{a}C)^h	0.0124	0.0049	0.0010	0.0050	78.5	60.8	94.6	147.1	*p*-CS/*t*-HB_{nc}

[a] See text for BSS-A. [b] Data are given at BCP, which is shown by A-∗-π, where one side interaction is shown if two are identical due to the symmetry. [c] $c\nabla^2\rho_b(\boldsymbol{r}_c) = H_b(\boldsymbol{r}_c) - V_b(\boldsymbol{r}_c)/2$, where $c = \hbar^2/8m$. [d] $R = (x^2 + y^2)^{1/2}$, where $(x, y) = (H_b(\boldsymbol{r}_c) - V_b(\boldsymbol{r}_c)/2, H_b(\boldsymbol{r}_c))$. [e] $\theta = 90° - \tan^{-1}(y/x)$, where $(x, y) = (H_b(\boldsymbol{r}_c) - V_b(\boldsymbol{r}_c)/2, H_b(\boldsymbol{r}_c))$. [f] $\theta_p = 90° - \tan^{-1}(dy/dx)$. [g] $\kappa_p = |d^2y/dx^2|/[1 + (dy/dx)^2]^{3/2}$. [h] Two imaginary frequencies being predicted for each.

Table S10. QTAIM functions and QTAIM-DFA parameters for X–H-*-π($C_{24}H_{12}$) and Y–X-*-π($C_{24}H_{12}$) (X, Y = F, Cl, Br and I) (C_{6v}: ID_{Cor} and ID_{Cor}), evaluated with MP2/BSS-A[a,b]

Y–X-*-π($C_{20}H_{10}$) (symm: type)	$\rho_b(\mathbf{r}_c)$ (ea_o^{-3})	$c\nabla^2\rho_b(\mathbf{r}_c)^c$ (au)	$H_b(\mathbf{r}_c)$ (au)	R^d (au)	θ^e (°)	freq (cm^{-1})	θ_p^f (°)	κ_p^g (au^{-1})	predicted nature
F–H-*-π (C_{6v}: ID_{Cor})[h]	0.0071	0.0033	0.0014	0.0036	67.0	84.2	68.8	60.5	*p*-CS/vdW
Cl–H-*-π (C_{6v}: ID_{Cor})[h]	0.0094	0.0039	0.0013	0.0041	72.0	76.0	73.7	51.8	*p*-CS/vdW
Br–H-*-π (C_{6v}: ID_{Cor})[h]	0.0100	0.0041	0.0013	0.0043	72.7	56.8	75.2	55.8	*p*-CS/vdW
I–H-*-π (C_{6v}: ID_{Cor})[h]	0.0108	0.0044	0.0013	0.0046	73.7	49.7	77.6	61.2	*p*-CS/vdW
F–F-*-π (C_{6v}: ID_{Cor})[h]	0.0063	0.0034	0.0012	0.0036	71.3	67.6	74.9	11.6	*p*-CS/vdW
Cl–Cl-*-π (C_{6v}: ID_{Cor})[h]	0.0086	0.0041	0.0016	0.0044	69.0	67.9	76.5	73.2	*p*-CS/vdW
Br–Br-*-π (C_{6v}: ID_{Cor})[h]	0.0089	0.0039	0.0014	0.0041	70.4	52.4	78.8	86.4	*p*-CS/vdW
I–I-*-π (C_{6v}: ID_{Cor})[h]	0.0101	0.0038	0.0011	0.0039	74.2	48.5	85.4	122.3	*p*-CS/vdW
F–Cl-*-π (C_{6v}: ID_{Cor})[h]	0.0079	0.0037	0.0014	0.0039	69.1	71.3	75.8	72.3	*p*-CS/vdW
F–Br-*-π (C_{6v}: ID_{Cor})[h]	0.0086	0.0037	0.0013	0.0039	70.8	60.1	78.8	87.3	*p*-CS/vdW
F–I-*-π (C_{6v}: ID_{Cor})[h]	0.0101	0.0038	0.0010	0.0039	75.1	57.7	86.8	130.2	*p*-CS/vdW

[a] See text for BSS-A. [b] Data are given at BCP, which is shown by A-*-π, where one side interaction is shown if two are identical due to the symmetry. [c] $c\nabla^2\rho_b(\mathbf{r}_c) = H_b(\mathbf{r}_c) - V_b(\mathbf{r}_c)/2$, where $c = \hbar^2/8m$. [d] $R = (x^2 + y^2)^{1/2}$, where $(x, y) = (H_b(\mathbf{r}_c) - V_b(\mathbf{r}_c)/2, H_b(\mathbf{r}_c))$. [e] $\theta = 90° - \tan^{-1}(y/x)$, where $(x, y) = (H_b(\mathbf{r}_c) - V_b(\mathbf{r}_c)/2, H_b(\mathbf{r}_c))$. [f] $\theta_p = 90° - \tan^{-1}(dy/dx)$. [g] $\kappa_p = |d^2y/dx^2|/[1 + (dy/dx)^2]^{3/2}$. [h] Two imaginary frequencies being predicted for each.

Table S11. QTAIM functions and QTAIM-DFA parameters for X–H-*-π(C_6H_6) and Y–X-*-π(C_6H_6) (X, Y = F, Cl, Br and I) (C_{6v}: ID_{Bzn} and ID_{Bzn}), evaluated with MP2/BSS-A[a,b]

Y–X-*-π($C_{20}H_{10}$) (symm: type)	$\rho_b(\mathbf{r}_c)$ (ea_o^{-3})	$c\nabla^2\rho_b(\mathbf{r}_c)^c$ (au)	$H_b(\mathbf{r}_c)$ (au)	R^d (au)	θ^e (°)	freq (cm^{-1})	θ_p^f (°)	κ_p^g (au^{-1})	predicted nature
F–H-*-π (C_{6v}: ID_{Bzn})[h]	0.0076	0.0036	0.0015	0.0039	67.4	110.2	69.5	61.4	*p*-CS/vdW
Cl–H-*-π (C_{6v}: ID_{Bzn})[h]	0.0087	0.0036	0.0012	0.0038	72.2	93.9	72.1	38.0	*p*-CS/vdW
Br–H-*-π (C_{6v}: ID_{Bzn})[h]	0.0088	0.0036	0.0011	0.0038	72.8	73.9	72.7	35.2	*p*-CS/vdW
I–H-*-π (C_{6v}: ID_{Bzn})[h]	0.0090	0.0036	0.0011	0.0038	73.5	66.1	74.1	33.0	*p*-CS/vdW
F–F-*-π (C_{6v}: ID_{Bzn})[h]	0.0049	0.0026	0.0008	0.0027	71.9	67.7	75.4	8.4	*p*-CS/vdW
Cl–Cl-*-π (C_{6v}: ID_{Bzn})[h]	0.0065	0.0030	0.0012	0.0033	68.0	79.4	72.1	54.8	*p*-CS/vdW
Br–Br-*-π (C_{6v}: ID_{Bzn})[h]	0.0065	0.0028	0.0011	0.0030	68.9	67.2	73.5	63.8	*p*-CS/vdW
I–I-*-π (C_{6v}: ID_{Bzn})[h]	0.0070	0.0026	0.0008	0.0027	72.2	64.5	77.8	76.0	*p*-CS/vdW
F–Cl-*-π (C_{6v}: ID_{Bzn})[h]	0.0062	0.0028	0.0011	0.0031	68.4	84.1	72.2	52.4	*p*-CS/vdW
F–Br-*-π (C_{6v}: ID_{Bzn})[h]	0.0064	0.0027	0.0010	0.0029	69.6	75.2	74.1	63.4	*p*-CS/vdW
F–I-*-π (C_{6v}: ID_{Bzn})[h]	0.0072	0.0027	0.0008	0.0028	73.0	74.3	79.2	82.0	*p*-CS/vdW

[a] See text for BSS-A. [b] Data are given at BCP, which is shown by A-*-π, where one side interaction is shown if two are identical due to the symmetry. [c] $c\nabla^2\rho_b(\mathbf{r}_c) = H_b(\mathbf{r}_c) - V_b(\mathbf{r}_c)/2$, where $c = \hbar^2/8m$. [d] $R = (x^2 + y^2)^{1/2}$, where $(x, y) = (H_b(\mathbf{r}_c) - V_b(\mathbf{r}_c)/2, H_b(\mathbf{r}_c))$. [e] $\theta = 90° - \tan^{-1}(y/x)$, where $(x, y) = (H_b(\mathbf{r}_c) - V_b(\mathbf{r}_c)/2, H_b(\mathbf{r}_c))$. [f] $\theta_p = 90° - \tan^{-1}(dy/dx)$. [g] $\kappa_p = |d^2y/dx^2|/[1 + (dy/dx)^2]^{3/2}$. [h] Two imaginary frequencies being predicted for each.

Table S12. ΔE_{ES} for X–H-∗-π and Y–X-∗-π adducts with π($C_{20}H_{10}$), π($C_{24}H_{12}$) and π(C_6H_6) (X, Y = F, Cl, Br and I) (type ID), evaluated with MP2/BSS-A, together with those differences $\Delta\Delta E_{ES}$[a]

species	$\Delta E_{ES:Cora}$[b] (kJ mol^{-1})	$\Delta\Delta E_{ES:Cora}$[c] (kJ mol^{-1})	$\Delta E_{ES:Cor}$[b] (kJ mol^{-1})	$\Delta\Delta E_{ES:Cor}$[c] (kJ mol^{-1})	$\Delta E_{ES:Bzn}$[b] (kJ mol^{-1})
Convex side					
F–H-∗-π(^{a}C)[d]	-20.3	3.5	-19.7	4.1	-23.7
Cl–H-∗-π(^{a}C)[d]	-24.4	0.3	-27.1	-2.4	-24.7
Br–H-∗-π(^{a}C)[d]	-25.8	-1.2	-29.5	-4.9	-24.6
I–H-∗-π(^{a}C)[d]	-27.0	-4.0	-31.9	-9.0	-23.0
F–F-∗-π(^{a}C)[d]	-14.6	-4.1	-15.6	-5.1	-10.5
Cl–Cl-∗-π(^{a}C)[d]	-30.5	-8.7	-34.6	-12.8	-21.8
Br–Br-∗-π(^{a}C)[d]	-33.8	-9.8	-39.1	-15.2	-23.9
I–I-∗-π(^{a}C)[d]	-40.6	-15.0	-46.8	-21.2	-25.6
F–Cl-∗-π(^{a}C)[d]	-27.9	-6.0	-30.6	-8.7	-21.9
F–Br-∗-π(^{a}C)[d]	-33.4	-8.0	-36.7	-11.3	-25.4
F–I-∗-π(^{a}C)[d]	-62.8	-33.9	-46.1	-17.2	-29.0
Concave side					
F–H-∗-π(^{a}C)[d]	-26.8	-3.1			
Cl–H-∗-π(^{a}C)[d]	-42.1	-17.3			
Br–H-∗-π(^{a}C)[d]	-46.0	-21.4			
I–H-∗-π(^{a}C)[d]	-8.4	14.6			
F–F-∗-π(^{a}C)[d]	-25.3	-14.8			
Cl–Cl-∗-π(^{a}C)[d]	-54.9	-33.1			
Br–Br-∗-π(^{a}C)[d]	-59.1	-35.2			
I–I-∗-π(^{a}C)[d]	-66.3	-40.7			
F–Cl-∗-π(^{a}C)[d]	-47.8	-25.9			
F–Br-∗-π(^{a}C)[d]	-54.4	-28.9			
F–I-∗-π(^{a}C)[d]	-43.1	-14.1			

[a] See text for BSS-A. [b] ΔE_{ES} = E_{ES}(X–H---π($C_{20}H_{10}$/$C_{24}H_{12}$/C_6H_6)/Y–X---π($C_{20}H_{10}$/$C_{24}H_{12}$/C_6H_6)) – (E_{ES}(X–H/Y–X) + E($C_{20}H_{10}$/$C_{24}H_{12}$/C_6H_6)). [c] $\Delta\Delta E_{ES}$ = ΔE_{ES}(X–H---π($C_{20}H_{10}$/$C_{24}H_{12}$)/Y–X---π($C_{20}H_{10}$/$C_{24}H_{12}$)) – ΔE_{ES}(X–H---π(C_6H_6)/Y–X---π(C_6H_6)). [d] Two imaginary frequencies being predicted for each.

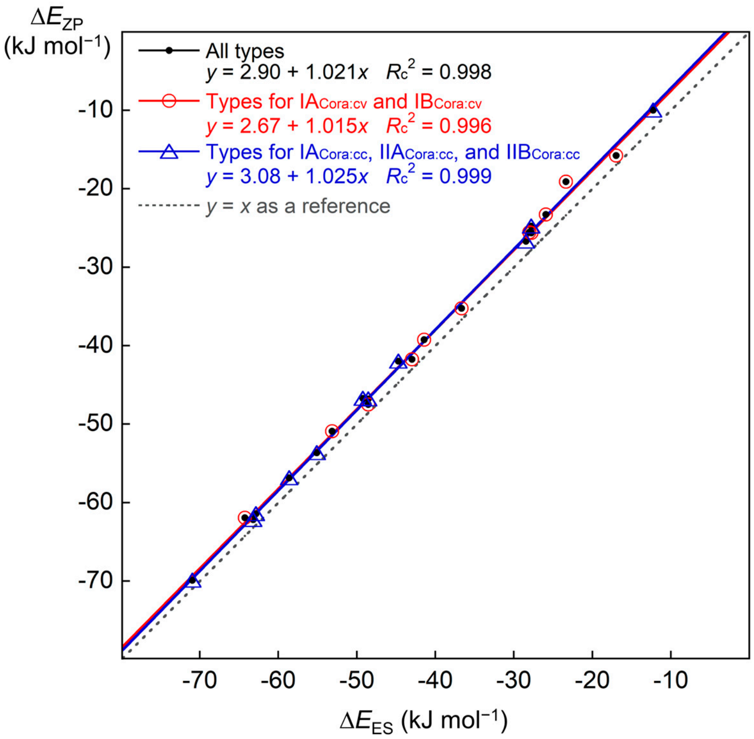

Figure S3. Plots of ΔE_{ZP} versus ΔE_{ES} for the optimized structures of B–A-∗-π($C_{20}H_{10}$), evaluated with MP2/BSS-A.

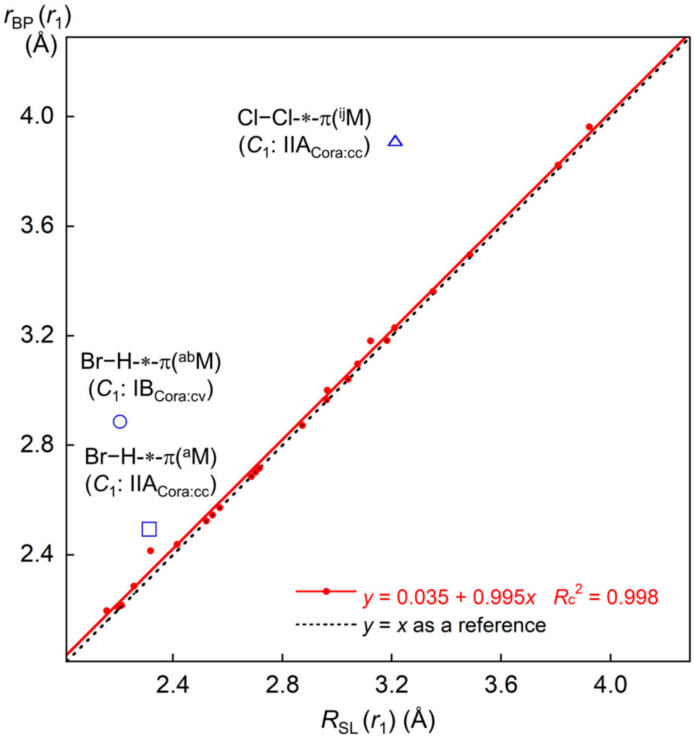

Figure S4. Plot of r_{BP} (r_1) versus R_{SL} (r_1) for the optimized structures of B–A-∗-π($C_{20}H_{10}$) with MP2/BSS-A. See Scheme 1 in the text. Br–H-∗-π(^{ab}M) (C_1: $\mathrm{IB}_{\mathrm{Cora:cv}}$), Br–H-∗-π(^{a}C) ($C_1$: $\mathrm{IIA}_{\mathrm{Cora:cc}}$) and Cl–Cl-∗-π(^{ij}M) (C_1: $\mathrm{IIA}_{\mathrm{Cora:cc}}$) are omitted.

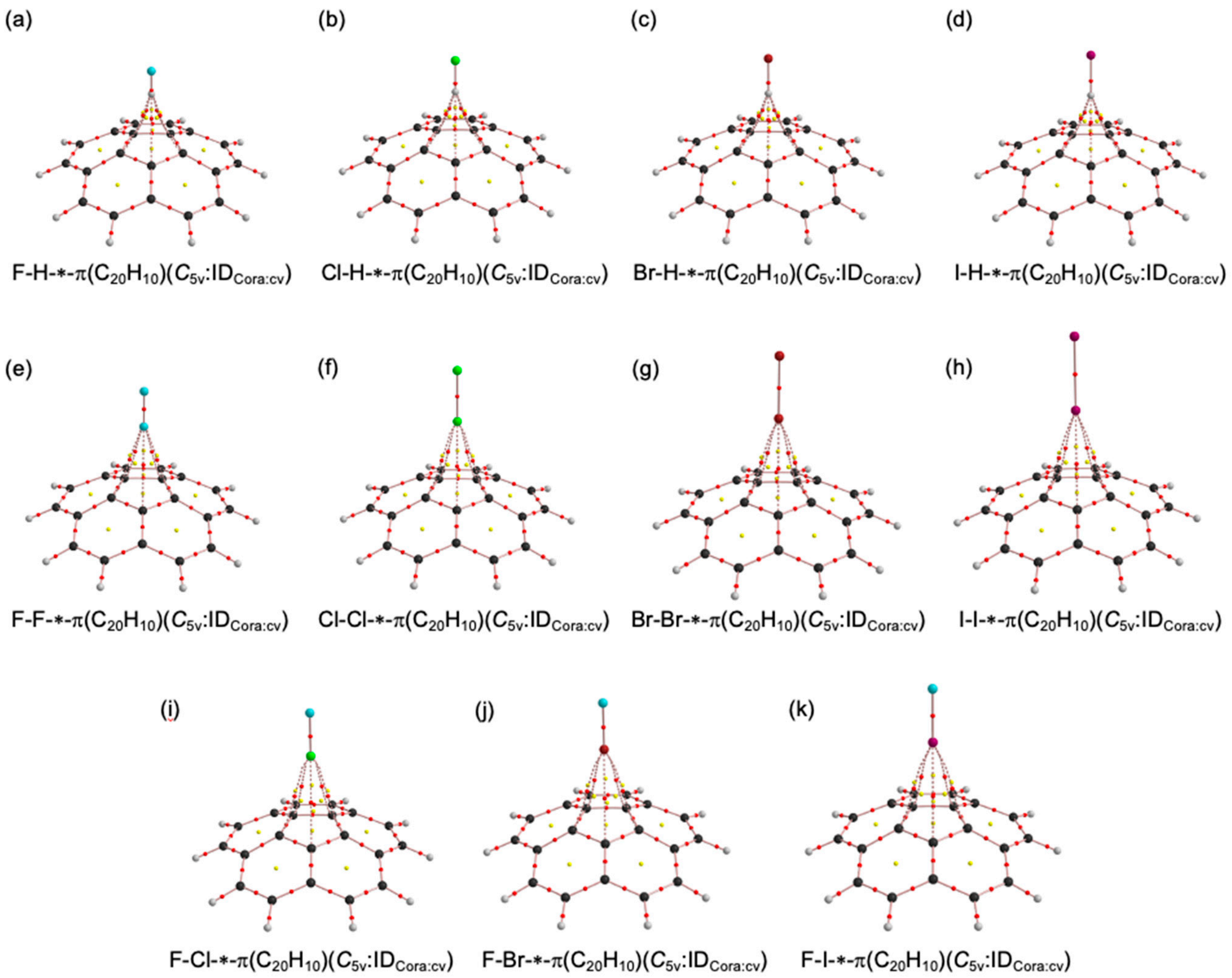

Figure S5. Molecular graphs for X–H-∗-π($C_{20}H_{10}$), X–X-∗-π($C_{20}H_{10}$) and F–X-∗-π($C_{20}H_{10}$) at convex (cv) side, evaluated with BSS-A: F–H-∗-π($C_{20}H_{10}$) (C_{5v}: type $ID_{Cora:cv}$) (a), Cl–H-∗-π($C_{20}H_{10}$) (C_{5v}: type $ID_{Cora:cv}$) (b), Br–H-∗-π($C_{20}H_{10}$) (C_{5v}: type $ID_{Cora:cv}$) (c), I–H-∗-π($C_{20}H_{10}$) (C_{5v}: type $ID_{Cora:cv}$) (d), F–F-∗-π($C_{20}H_{10}$) (C_{5v}: type $ID_{Cora:cv}$) (e), Cl–Cl-∗-π($C_{20}H_{10}$) (C_{5v}: type $ID_{Cora:cv}$) (f), Br–Br-∗-π($C_{20}H_{10}$) (C_{5v}: type $ID_{Cora:cv}$) (g), I–I-∗-π($C_{20}H_{10}$) (C_{5v}: type $ID_{Cora:cv}$) (h), F–Cl-∗-π($C_{20}H_{10}$) (C_{5v}: type $ID_{Cora:cv}$) (i), F–Br-∗-π($C_{20}H_{10}$) (C_{5v}: type $ID_{Cora:cv}$) (j) and F–I-∗-π($C_{20}H_{10}$) (C_{5v}: type $ID_{Cora:cv}$) (k). BPs are drawn as pink lines, BCPs as red dots, RCPs (ring critical points) as yellow dots and CCPs (cage critical points) as green dots. Carbon atoms are indicated in black and hydrogen atoms are in grey, with fluorine, chlorine, bromine and iodine atoms in dark yellow, green, dark brown and dark purple, respectively.

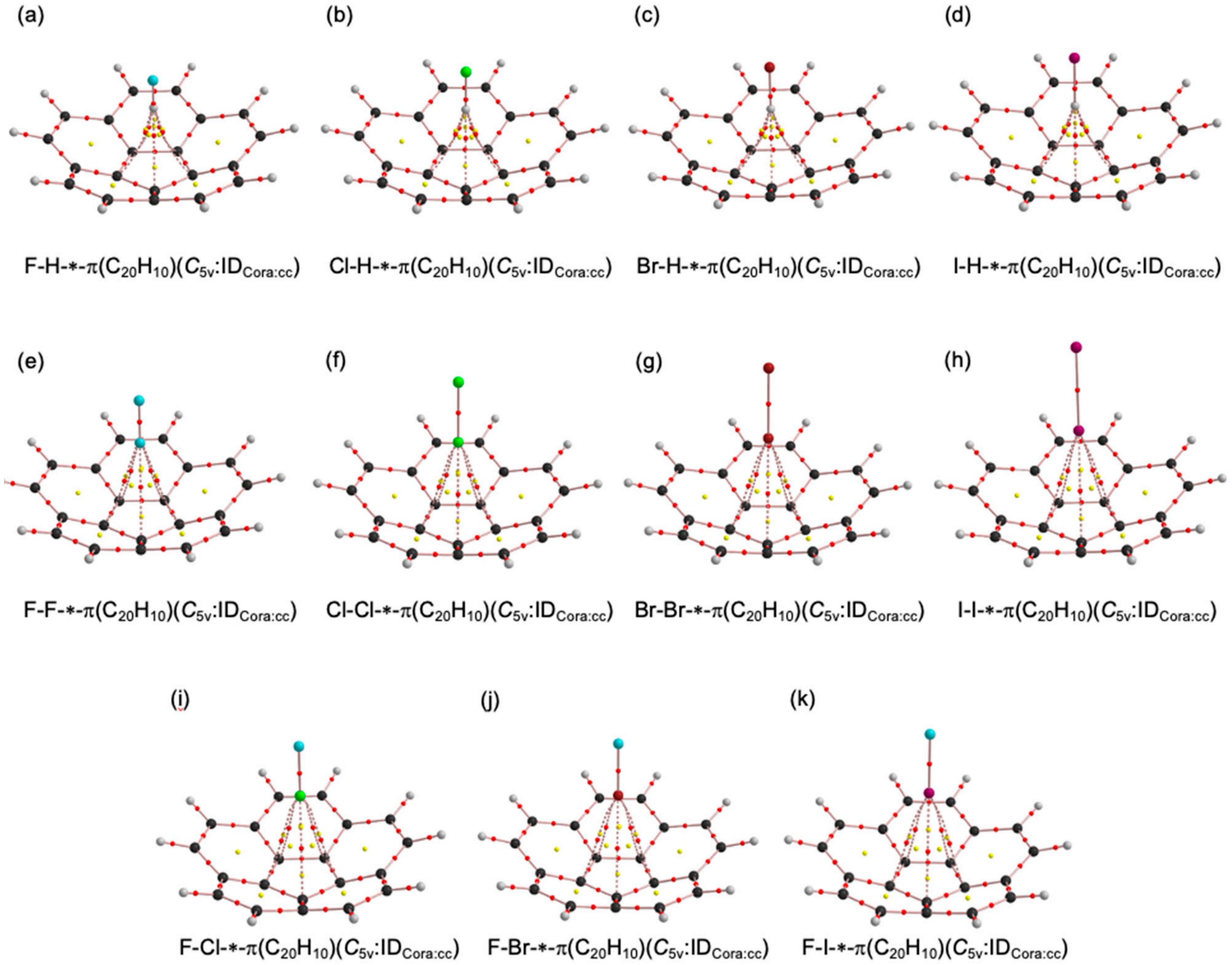

Figure S6. Molecular graphs for X–H-∗-π($C_{20}H_{10}$), X–X-∗-π($C_{20}H_{10}$) and F–X-∗-π($C_{20}H_{10}$) at concave (cc) side, evaluated with BSS-A: F–H-∗-π($C_{20}H_{10}$) (C_{5v}: type $ID_{Cora:cc}$) (a), Cl–H-∗-π($C_{20}H_{10}$) (C_{5v}: type $ID_{Cora:cc}$) (b), Br–H-∗-π($C_{20}H_{10}$) (C_{5v}: type $ID_{Cora:cc}$) (c), I–H-∗-π($C_{20}H_{10}$) (C_{5v}: type $ID_{Cora:cc}$) (d), F–F-∗-π($C_{20}H_{10}$) (C_{5v}: type $ID_{Cora:cc}$) (e), Cl–Cl-∗-π($C_{20}H_{10}$) (C_{5v}: type $ID_{Cora:cc}$) (f), Br–Br-∗-π($C_{20}H_{10}$) (C_{5v}: type $ID_{Cora:cc}$) (g), I–I-∗-π($C_{20}H_{10}$) (C_{5v}: type $ID_{Cora:cc}$) (h), F–Cl-∗-π($C_{20}H_{10}$) (C_{5v}: type $ID_{Cora:cc}$) (i), F–Br-∗-π($C_{20}H_{10}$) (C_{5v}: type $ID_{Cora:cc}$ (j) and F–I-∗-π($C_{20}H_{10}$) (C_{5v}: type $ID_{Cora:cc}$) (k). BPs are drawn as pink lines, BCPs as red dots, RCPs (ring critical points) as yellow dots and CCPs (cage critical points) as green dots. Carbon atoms are indicated in black and hydrogen atoms are in grey, with fluorine, chlorine, bromine and iodine atoms in dark yellow, green, dark brown and dark purple, respectively.

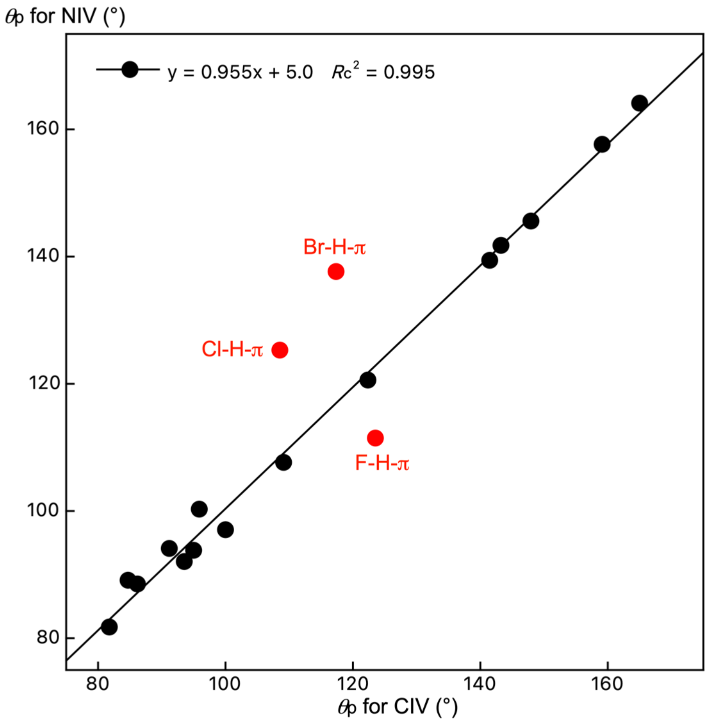

Figure S7. Plot of θ_P for NIV versus θ_P for CIV for the optimized structures of B–A-∗-π($C_{20}H_{10}$) with MP2/BSS-A. F–H-∗-π(^{a}C) (C_1: $IA_{Cora:cv}$), Cl–H-∗-π(^{a}C) (C_s: $IIA_{Cora:cc}$), and Br–H-∗-π(^{a}C) (C_1: $IIA_{Cora:cc}$) are omitted.

Optimized structures given by Cartesian coordinates

Optimized structures given by Cartesian coordinates for examined molecules, together with the total energies calculated with the MP2/BSS-A method of the Gaussian 09 program package. The BSS-A is 6-311G(2d,p) basis sets applied to C and H, together with the basis set of 6-311+G(3df) for F, Cl, and Br and the (7433211/743111/7411/2 + 1s1p) type for I, implemented from the Sapporo Basis Set Factory. The optimized structures were confirmed by the frequency analysis. All structures have all positive frequencies, except for $C_{20}H_{10}$ (type $ID_{Cora:cv}$ and type $ID_{Cora:cc}$), $C_{24}H_{12}$ (type ID_{Cor}) and C_6H_6 (type ID_{Bzn}), which have two imaginary ones. $C_{12}H_{24}$ was optimized by the C_i symmetry reduced from D_{6h}, since imaginary frequencies were detected for full planer structures calculated with MP2 level of theory.

MP2/BSS-A
Adduct $C_{20}H_{10}$
Symmetry C_{5v}
Energy MP2 = −766.192999 au
Standard Orientation

6	0	0.000000	1.201919	0.644592
6	0	-1.143093	0.371413	0.644592
6	0	1.143093	0.371413	0.644592
6	0	-0.706470	-0.972373	0.644592
6	0	0.706470	-0.972373	0.644592
6	0	0.000000	2.479265	0.098113
6	0	-2.357921	0.766135	0.098113
6	0	2.357921	0.766135	0.098113
6	0	-1.457275	-2.005768	0.098113
6	0	1.457275	-2.005768	0.098113
6	0	1.301667	2.977158	-0.264348
6	0	-3.233683	-0.317966	-0.264348
6	0	2.429209	2.157951	-0.264348
6	0	-2.803000	-1.643471	-0.264348
6	0	3.233683	-0.317966	-0.264348
6	0	-0.696859	-3.173672	-0.264348
6	0	2.803000	-1.643471	-0.264348
6	0	0.696859	-3.173672	-0.264348
6	0	-1.301667	2.977158	-0.264348
6	0	-2.429209	2.157951	-0.264348
1	0	1.403035	3.992174	-0.642028
1	0	3.363222	2.568015	-0.642028
1	0	4.230345	-0.100715	-0.642028
1	0	3.481620	-2.405054	-0.642028
1	0	1.211462	-4.054420	-0.642028
1	0	-1.211462	-4.054420	-0.642028
1	0	-3.481620	-2.405054	-0.642028
1	0	-4.230345	-0.100715	-0.642028
1	0	-3.363222	2.568015	-0.642028
1	0	-1.403035	3.992174	-0.642028

MP2/BSS-A
Adduct $C_{20}H_{10}$−HF (type: $IA_{Cora:cv}$)
Symmetry C_1

Energy MP2 = −866.528641 au
Standard Orientation

6	0	0.663116	0.923137	0.301280
6	0	1.001877	-0.450045	0.246636
6	0	-0.181799	-1.199422	0.437506
6	0	-1.249328	-0.291362	0.604877
6	0	-0.727854	1.018681	0.523471
6	0	1.391589	1.902084	-0.363980
6	0	2.637530	1.446785	-0.921996
6	0	2.972850	0.094037	-0.975051
1	0	3.303672	2.159846	-1.402135
1	0	3.885853	-0.189856	-1.493023
6	0	2.091496	-0.927998	-0.475670
6	0	2.010505	-2.321789	-0.825226
6	0	0.841649	-3.059279	-0.641869
1	0	2.845423	-2.797665	-1.334182
1	0	0.814477	-4.079435	-1.018083
6	0	-0.352105	-2.473428	-0.089318
6	0	-1.724897	-2.880655	-0.240179
6	0	-2.779815	-1.984801	-0.074108
1	0	-1.952695	-3.886144	-0.586390
1	0	-3.786815	-2.329388	-0.298136
6	0	-2.559061	-0.601237	0.258054
6	0	-3.403130	0.540677	0.023084
6	0	-2.887829	1.833789	-0.057596
1	0	-4.458354	0.393196	-0.195306
1	0	-3.562846	2.639986	-0.335624
6	0	-1.481423	2.102307	0.089316
6	0	-0.707462	3.215026	-0.397564
6	0	0.666221	3.120453	-0.613637
1	0	-1.212918	4.129894	-0.698396
1	0	1.175262	3.965023	-1.072130
1	0	2.094771	-0.084555	2.070828
9	0	2.613488	0.004150	2.834933

MP2/BSS-A
Adduct $C_{20}H_{10}$−HCl (type: $IB_{Cora:cv}$)
Symmetry C_s
Energy MP2 = −1226.500001 au
Standard Orientation

6	0	-0.009076	-0.659803	0.706002
6	0	-0.009076	-0.659803	-0.706002
6	0	-0.431340	0.618593	-1.142458
6	0	-0.687025	1.405652	0.000000
6	0	-0.431340	0.618593	1.142458
6	0	0.827598	-1.479196	1.455954
6	0	1.523330	-2.481459	0.693015
6	0	1.523330	-2.481459	-0.693015
1	0	2.144435	-3.212590	1.205221
1	0	2.144435	-3.212590	-1.205221
6	0	0.827598	-1.479196	-1.455954
6	0	1.052918	-1.018546	-2.807996

6	0	0.642716	0.243650	-3.234855
1	0	1.641923	-1.627048	-3.490264
1	0	0.931539	0.567134	-4.232339
6	0	-0.034761	1.163187	-2.357479
6	0	-0.120428	2.598647	-2.426730
6	0	-0.373130	3.376303	-1.297490
1	0	0.111884	3.106806	-3.359858
1	0	-0.327206	4.458359	-1.397786
6	0	-0.563514	2.789940	0.000000
6	0	-0.373130	3.376303	1.297490
6	0	-0.120428	2.598647	2.426730
1	0	-0.327206	4.458359	1.397786
1	0	0.111884	3.106806	3.359858
6	0	-0.034761	1.163187	2.357479
6	0	0.642716	0.243650	3.234855
6	0	1.052918	-1.018546	2.807996
1	0	0.931539	0.567134	4.232339
1	0	1.641923	-1.627048	3.490264
1	0	-1.462254	-2.327239	0.000000
17	0	-2.174917	-3.398127	0.000000

MP2/BSS-A
Adduct $C_{20}H_{10}$−HBr (type: $IB_{Cora:cv}$)
Symmetry C_1
Energy MP2 = −3339.514026 au
Standard Orientation

6	0	-0.214366	0.707459	0.286619
6	0	-0.214519	-0.707216	0.286702
6	0	0.957966	-1.142975	-0.369062
6	0	1.683749	-0.000146	-0.770529
6	0	0.958211	1.142885	-0.369193
6	0	-0.860051	1.459272	1.262036
6	0	-1.714760	0.698139	2.134070
6	0	-1.714907	-0.697356	2.134159
1	0	-2.315256	1.211497	2.881495
1	0	-2.315509	-1.210496	2.881648
6	0	-0.860358	-1.458776	1.262218
6	0	-0.371234	-2.805676	1.398809
6	0	0.789859	-3.234693	0.757109
1	0	-0.858323	-3.487022	2.092525
1	0	1.160453	-4.232760	0.979760
6	0	1.565880	-2.358956	-0.082701
6	0	2.958947	-2.430305	-0.439074
6	0	3.676211	-1.302277	-0.835348
1	0	3.500667	-3.364133	-0.307002
1	0	4.747178	-1.404303	-0.995483
6	0	3.066321	-0.000303	-0.911906
6	0	3.676490	1.301549	-0.835500
6	0	2.959467	2.429779	-0.439360
1	0	4.747479	1.403327	-0.995645
1	0	3.501389	3.363504	-0.307396
6	0	1.566385	2.358772	-0.082980

6	0	0.790549	3.234777	0.756723
6	0	-0.370638	2.806085	1.398469
1	0	1.161356	4.232791	0.979254
1	0	-0.857584	3.487619	2.092100
1	0	-2.113121	-0.000468	-0.836361
35	0	-3.438113	0.000006	-1.363213

MP2/BSS-A
Adduct $C_{20}H_{10}$−HI (type: $IB_{Cora:cv}$)
Symmetry C_s
Energy MP2 = −7684.863174 au
Standard Orientation

6	0	-0.424248	-0.223111	0.706002
6	0	-0.424248	-0.223111	-0.706002
6	0	0.357686	-1.319096	-1.142458
6	0	0.834998	-1.995121	0.000000
6	0	0.357686	-1.319096	1.142458
6	0	-1.466060	0.311730	1.455954
6	0	-2.427413	1.062979	0.693015
6	0	-2.427413	1.062979	-0.693015
1	0	-3.237188	1.577361	1.205221
1	0	-3.237188	1.577361	-1.205221
6	0	-1.466060	0.311730	-1.455954
6	0	-1.544844	-0.194985	-2.807996
6	0	-0.779229	-1.279069	-3.234855
1	0	-2.287641	0.211776	-3.490264
1	0	-0.959291	-1.673580	-4.232339
6	0	0.140187	-1.956710	-2.357479
6	0	0.647141	-3.302400	-2.426730
6	0	1.118820	-3.970327	-1.297490
1	0	0.575750	-3.856564	-3.359858
1	0	1.395421	-5.017440	-1.397786
6	0	1.127003	-3.353885	0.000000
6	0	1.118820	-3.970327	1.297490
6	0	0.647141	-3.302400	2.426730
1	0	1.395421	-5.017440	1.397786
1	0	0.575750	-3.856564	3.359858
6	0	0.140187	-1.956710	2.357479
6	0	-0.779229	-1.279069	3.234855
6	0	-1.544844	-0.194985	2.807996
1	0	-0.959291	-1.673580	4.232339
1	0	-2.287641	0.211776	3.490264
1	0	0.469904	1.799897	0.000000
53	0	0.930557	3.363451	0.000000

MP2/BSS-A
Adduct $C_{20}H_{10}$−F_2 (type: $IA_{Cora:cv}$)
Symmetry C_s
Energy MP2 = −965.466833 au
Standard Orientation

6	0	-0.146028	-0.157223	1.143062
6	0	0.117781	-0.944457	0.000000

6	0	-0.146028	-0.157223	-1.143062
6	0	-0.572791	1.115772	-0.706492
6	0	-0.572791	1.115772	0.706492
6	0	0.493209	-0.361352	2.359088
6	0	1.273607	-1.569645	2.429662
6	0	1.532741	-2.348108	1.302304
1	0	1.757929	-1.842700	3.364605
1	0	2.207432	-3.194956	1.405756
6	0	1.037178	-1.987767	0.000000
6	0	1.532741	-2.348108	-1.302304
6	0	1.273607	-1.569645	-2.429662
1	0	2.207432	-3.194956	-1.405756
1	0	1.757929	-1.842700	-3.364605
6	0	0.493209	-0.361352	-2.359088
6	0	0.492710	0.781211	-3.235371
6	0	0.074104	2.039034	-2.804132
1	0	0.917110	0.694274	-4.233086
1	0	0.190072	2.880483	-3.483575
6	0	-0.382953	2.268955	-1.458008
6	0	-0.409068	3.491083	-0.697068
6	0	-0.409068	3.491083	0.697068
1	0	-0.329631	4.446354	-1.211166
1	0	-0.329631	4.446354	1.211166
6	0	-0.382953	2.268955	1.458008
6	0	0.074104	2.039034	2.804132
6	0	0.492710	0.781211	3.235371
1	0	0.190072	2.880483	3.483575
1	0	0.917110	0.694274	4.233086
9	0	-1.896631	-2.461204	0.000000
9	0	-3.068030	-3.259939	0.000000

MP2/BSS-A
Adduct $C_{20}H_{10}–Cl_2$ (type: $IA_{Cora:cv}$)
Symmetry C_s
Energy MP2 = −1685.565071 au
Standard Orientation

6	0	-0.134609	-0.209295	1.143290
6	0	-0.648955	0.444342	0.000000
6	0	-0.134609	-0.209295	-1.143290
6	0	0.699492	-1.259329	-0.706443
6	0	0.699492	-1.259329	0.706443
6	0	-0.803084	-0.231141	2.360257
6	0	-1.945082	0.643023	2.430712
6	0	-2.455189	1.286245	1.302546
1	0	-2.491080	0.739463	3.366591
1	0	-3.375258	1.856661	1.407780
6	0	-1.871214	1.111917	0.000000
6	0	-2.455189	1.286245	-1.302546
6	0	-1.945082	0.643023	-2.430712
1	0	-3.375258	1.856661	-1.407780
1	0	-2.491080	0.739463	-3.366591
6	0	-0.803084	-0.231141	-2.360257

6	0	-0.412622	-1.304551	-3.237226
6	0	0.408137	-2.345440	-2.805319
1	0	-0.838827	-1.365605	-4.236005
1	0	0.586518	-3.175149	-3.485626
6	0	0.915084	-2.407866	-1.458671
6	0	1.354326	-3.547712	-0.697413
6	0	1.354326	-3.547712	0.697413
1	0	1.603495	-4.473598	-1.210970
1	0	1.603495	-4.473598	1.210970
6	0	0.915084	-2.407866	1.458671
6	0	0.408137	-2.345440	2.805319
6	0	-0.412622	-1.304551	3.237226
1	0	0.586518	-3.175149	3.485626
1	0	-0.838827	-1.365605	4.236005
17	0	0.932985	2.615716	0.000000
17	0	2.163125	4.208502	0.000000

MP2/BSS-A
Adduct $C_{20}H_{10}$−Br_2 (type: $IA_{Cora:cv}$)
Symmetry C_s
Energy MP2 = −5911.628544 au
Standard Orientation

6	0	-0.230590	-0.956918	1.143734
6	0	-0.883474	-0.438937	0.000000
6	0	-0.230590	-0.956918	-1.143734
6	0	0.826445	-1.780293	-0.706456
6	0	0.826445	-1.780293	0.706456
6	0	-0.875600	-1.135776	2.360339
6	0	-2.191367	-0.555819	2.430353
6	0	-2.839705	-0.051239	1.302035
1	0	-2.745015	-0.591254	3.366064
1	0	-3.868592	0.285175	1.407508
6	0	-2.232080	-0.082552	0.000000
6	0	-2.839705	-0.051239	-1.302035
6	0	-2.191367	-0.555819	-2.430353
1	0	-3.868592	0.285175	-1.407508
1	0	-2.745015	-0.591254	-3.366064
6	0	-0.875600	-1.135776	-2.360339
6	0	-0.242766	-2.086378	-3.237724
6	0	0.800509	-2.904016	-2.805814
1	0	-0.642284	-2.245598	-4.236650
1	0	1.170160	-3.667699	-3.486352
6	0	1.307455	-2.845574	-1.458940
6	0	2.002124	-3.849597	-0.697625
6	0	2.002124	-3.849597	0.697625
1	0	2.461523	-4.691344	-1.210913
1	0	2.461523	-4.691344	1.210913
6	0	1.307455	-2.845574	1.458940
6	0	0.800509	-2.904016	2.805814
6	0	-0.242766	-2.086378	3.237724
1	0	1.170160	-3.667699	3.486352
1	0	-0.642284	-2.245598	4.236650

35	0	0.148080	2.073757	0.000000
35	0	1.088025	4.181606	0.000000

MP2/BSS-A
Adduct $C_{20}H_{10}$−I_2 (type: $IA_{Cora:cv}$)
Symmetry C_s
Energy MP2 = −14602.365869 au
Standard Orientation

6	0	-0.243202	-1.592295	1.143645
6	0	-0.966119	-1.177517	0.000000
6	0	-0.243202	-1.592295	-1.143645
6	0	0.923272	-2.250892	-0.706187
6	0	0.923272	-2.250892	0.706187
6	0	-0.854415	-1.862731	2.360773
6	0	-2.240249	-1.480197	2.431290
6	0	-2.956268	-1.077181	1.302501
1	0	-2.782387	-1.594488	3.367426
1	0	-4.023179	-0.894577	1.408895
6	0	-2.351886	-1.021730	0.000000
6	0	-2.956268	-1.077181	-1.302501
6	0	-2.240249	-1.480197	-2.431290
1	0	-4.023179	-0.894577	-1.408895
1	0	-2.782387	-1.594488	-3.367426
6	0	-0.854415	-1.862731	-2.360773
6	0	-0.087212	-2.708010	-3.238714
6	0	1.065276	-3.363065	-2.806670
1	0	-0.457816	-2.923124	-4.238297
1	0	1.544247	-4.062619	-3.487990
6	0	1.557403	-3.232143	-1.459195
6	0	2.392653	-4.122458	-0.697734
6	0	2.392653	-4.122458	0.697734
1	0	2.971778	-4.886868	-1.210874
1	0	2.971778	-4.886868	1.210874
6	0	1.557403	-3.232143	1.459195
6	0	1.065276	-3.363065	2.806670
6	0	-0.087212	-2.708010	3.238714
1	0	1.544247	-4.062619	3.487990
1	0	-0.457816	-2.923124	4.238297
53	0	-0.123492	1.569033	0.000000
53	0	0.703033	4.132599	0.000000

MP2/BSS-A
Adduct $C_{20}H_{10}$−ClF (type: $IA_{Cora:cv}$)
Symmetry C_s
Energy MP2 = −1325.546120 au
Standard Orientation

6	0	-0.029431	-0.019536	1.144068
6	0	0.307113	-0.783334	0.000000
6	0	-0.029431	-0.019536	-1.144068
6	0	-0.586060	1.198436	-0.706589
6	0	-0.586060	1.198436	0.706589
6	0	0.627215	-0.157097	2.359392

6	0	1.528847	-1.277217	2.429034
6	0	1.871137	-2.023975	1.301132
1	0	2.038007	-1.499473	3.364249
1	0	2.630195	-2.795717	1.405718
6	0	1.344234	-1.717489	0.000000
6	0	1.871137	-2.023975	-1.301132
6	0	1.528847	-1.277217	-2.429034
1	0	2.630195	-2.795717	-1.405718
1	0	2.038007	-1.499473	-3.364249
6	0	0.627215	-0.157097	-2.359392
6	0	0.505035	0.978289	-3.237078
6	0	-0.044156	2.184459	-2.805498
1	0	0.933971	0.935146	-4.235499
1	0	-0.019821	3.032734	-3.485765
6	0	-0.521512	2.365939	-1.458849
6	0	-0.674507	3.576837	-0.697712
6	0	-0.674507	3.576837	0.697712
1	0	-0.692799	4.535632	-1.210928
1	0	-0.692799	4.535632	1.210928
6	0	-0.521512	2.365939	1.458849
6	0	-0.044156	2.184459	2.805498
6	0	0.505035	0.978289	3.237078
1	0	-0.019821	3.032734	3.485765
1	0	0.933971	0.935146	4.235499
17	0	-1.550150	-2.521956	0.000000
9	0	-2.828162	-3.605788	0.000000

MP2/BSS-A
Adduct $C_{20}H_{10}$−BrF (type: $IA_{Cora:cv}$)
Symmetry C_s
Energy MP2 = −3438.587958 au
Standard Orientation

6	0	0.108280	0.266593	1.144661
6	0	0.541424	-0.449651	0.000000
6	0	0.108280	0.266593	-1.144661
6	0	-0.606936	1.397060	-0.706588
6	0	-0.606936	1.397060	0.706588
6	0	0.779577	0.220422	2.358861
6	0	1.825896	-0.765068	2.427911
6	0	2.266844	-1.458991	1.300057
1	0	2.362358	-0.914033	3.362420
1	0	3.125158	-2.118520	1.404334
6	0	1.702284	-1.229510	0.000000
6	0	2.266844	-1.458991	-1.300057
6	0	1.825896	-0.765068	-2.427911
1	0	3.125158	-2.118520	-1.404334
1	0	2.362358	-0.914033	-3.362420
6	0	0.779577	0.220422	-2.358861
6	0	0.503986	1.328387	-3.237154
6	0	-0.204834	2.448007	-2.805861
1	0	0.934977	1.343454	-4.235419
1	0	-0.296953	3.291458	-3.486254

6	0	-0.702171	2.562940	-1.458991
6	0	-1.016769	3.741265	-0.698018
6	0	-1.016769	3.741265	0.698018
1	0	-1.162924	4.689162	-1.210940
1	0	-1.162924	4.689162	1.210940
6	0	-0.702171	2.562940	1.458991
6	0	-0.204834	2.448007	2.805861
6	0	0.503986	1.328387	3.237154
1	0	-0.296953	3.291458	3.486254
1	0	0.934977	1.343454	4.235419
35	0	-1.088436	-2.438036	0.000000
9	0	-2.304303	-3.784910	0.000000

MP2/BSS-A
Adduct $C_{20}H_{10}$−IF (type: $IA_{Cora:cv}$)
Symmetry C_1
Energy MP2 = −7783.976623 au
Standard Orientation

6	0	-0.538204	-1.144696	0.205502
6	0	0.119699	0.000083	0.723483
6	0	-0.538293	1.144743	0.205355
6	0	-1.572776	0.706166	-0.642020
6	0	-1.572725	-0.706308	-0.641924
6	0	-0.575023	-2.358688	0.878203
6	0	0.275033	-2.427731	2.036329
6	0	0.910126	-1.299580	2.559162
1	0	0.356949	-3.361985	2.587450
1	0	1.459482	-1.404188	3.491794
6	0	0.751814	0.000184	1.971298
6	0	0.910007	1.300032	2.559011
6	0	0.274813	2.428065	2.036042
1	0	1.459351	1.404801	3.491630
1	0	0.356645	3.362389	2.587052
6	0	-0.575220	2.358813	0.877911
6	0	-1.639979	3.237731	0.466846
6	0	-2.663651	2.806530	-0.374526
1	0	-1.707208	4.236335	0.891936
1	0	-3.488554	3.487513	-0.570963
6	0	-2.717486	1.458963	-0.881155
6	0	-3.848050	0.698015	-1.337346
6	0	-3.847995	-0.698427	-1.337259
1	0	-4.771037	1.210738	-1.598335
1	0	-4.770944	-1.211255	-1.598183
6	0	-2.717374	-1.459227	-0.880963
6	0	-2.663431	-2.806723	-0.374163
6	0	-1.639717	-3.237740	0.467255
1	0	-3.488278	-3.487801	-0.570520
1	0	-1.706860	-4.236302	0.892460
53	0	2.344206	-0.000013	-0.810095
9	0	3.918683	-0.000084	-1.974614

Adduct $C_{20}H_{10}$−HF (type: $ID_{Cora:cv}$)
Symmetry C_{5v}
Energy MP2 = −866.527462 au
Standard Orientation

6	0	0.000000	1.202172	0.372081
6	0	-1.143333	0.371492	0.372081
6	0	1.143333	0.371492	0.372081
6	0	-0.706619	-0.972578	0.372081
6	0	0.706619	-0.972578	0.372081
6	0	0.000000	2.481915	-0.169484
6	0	-2.360441	0.766954	-0.169484
6	0	2.360441	0.766954	-0.169484
6	0	-1.458833	-2.007911	-0.169484
6	0	1.458833	-2.007911	-0.169484
6	0	-1.302188	2.978520	-0.529743
6	0	-2.430343	2.158868	-0.529743
1	0	-1.404881	3.993974	-0.905495
1	0	-3.364363	2.570327	-0.905495
6	0	1.302188	2.978520	-0.529743
6	0	2.430343	2.158868	-0.529743
1	0	1.404881	3.993974	-0.905495
1	0	3.364363	2.570327	-0.905495
6	0	-3.235139	-0.318041	-0.529743
6	0	-2.804222	-1.644266	-0.529743
1	0	-4.232627	-0.101915	-0.905495
1	0	-3.484172	-2.405425	-0.905495
6	0	3.235139	-0.318041	-0.529743
6	0	2.804222	-1.644266	-0.529743
1	0	4.232627	-0.101915	-0.905495
1	0	3.484172	-2.405425	-0.905495
6	0	-0.697238	-3.175080	-0.529743
1	0	-1.211026	-4.056961	-0.905495
6	0	0.697238	-3.175080	-0.529743
1	0	1.211026	-4.056961	-0.905495
1	0	0.000000	0.000000	2.644435
9	0	0.000000	0.000000	3.568577

Adduct $C_{20}H_{10}$−HCl (type: $ID_{Cora:cv}$)
Symmetry C_{5v}
Energy MP2 = −1226.499431 au
Standard Orientation

6	0	0.000000	1.201903	0.140200
6	0	-1.143078	0.371408	0.140200
6	0	1.143078	0.371408	0.140200
6	0	-0.706461	-0.972360	0.140200
6	0	0.706461	-0.972360	0.140200
6	0	0.000000	2.482052	-0.400338
6	0	-2.360572	0.766996	-0.400338
6	0	2.360572	0.766996	-0.400338
6	0	-1.458914	-2.008022	-0.400338
6	0	1.458914	-2.008022	-0.400338
6	0	-1.302445	2.978949	-0.759716

6	0	-2.430671	2.159245	-0.759716
1	0	-1.405458	3.994757	-1.134528
1	0	-3.364929	2.571118	-1.134528
6	0	1.302445	2.978949	-0.759716
6	0	2.430671	2.159245	-0.759716
1	0	1.405458	3.994757	-1.134528
1	0	3.364929	2.571118	-1.134528
6	0	-3.235626	-0.318153	-0.759716
6	0	-2.804682	-1.644462	-0.759716
1	0	-4.233550	-0.102222	-1.134528
1	0	-3.485099	-2.405719	-1.134528
6	0	3.235626	-0.318153	-0.759716
6	0	2.804682	-1.644462	-0.759716
1	0	4.233550	-0.102222	-1.134528
1	0	3.485099	-2.405719	-1.134528
6	0	-0.697282	-3.175578	-0.759716
1	0	-1.211020	-4.057934	-1.134528
6	0	0.697282	-3.175578	-0.759716
1	0	1.211020	-4.057934	-1.134528
1	0	0.000000	0.000000	2.385703
17	0	0.000000	0.000000	3.667450

Adduct $C_{20}H_{10}$−HBr (type: $ID_{Cora:cv}$)
Symmetry C_{5v}
Energy MP2 = −3339.513281 au
Standard Orientation

6	0	0.000000	1.201793	-0.286313
6	0	-1.142973	0.371374	-0.286313
6	0	1.142973	0.371374	-0.286313
6	0	-0.706396	-0.972271	-0.286313
6	0	0.706396	-0.972271	-0.286313
6	0	0.000000	2.482122	-0.826361
6	0	-2.360638	0.767018	-0.826361
6	0	2.360638	0.767018	-0.826361
6	0	-1.458955	-2.008079	-0.826361
6	0	1.458955	-2.008079	-0.826361
6	0	-1.302567	2.979164	-1.185274
6	0	-2.430838	2.159427	-1.185274
1	0	-1.405742	3.995133	-1.559638
1	0	-3.365199	2.571504	-1.559638
6	0	1.302567	2.979164	-1.185274
6	0	2.430838	2.159427	-1.185274
1	0	1.405742	3.995133	-1.559638
1	0	3.365199	2.571504	-1.559638
6	0	-3.235869	-0.318203	-1.185274
6	0	-2.804908	-1.644565	-1.185274
1	0	-4.233995	-0.102376	-1.559638
1	0	-3.485549	-2.405856	-1.559638
6	0	3.235869	-0.318203	-1.185274
6	0	2.804908	-1.644565	-1.185274
1	0	4.233995	-0.102376	-1.559638
1	0	3.485549	-2.405856	-1.559638

6	0	-0.697310	-3.175824	-1.185274
1	0	-1.211011	-4.058405	-1.559638
6	0	0.697310	-3.175824	-1.185274
1	0	1.211011	-4.058405	-1.559638
1	0	0.000000	0.000000	1.955468
35	0	0.000000	0.000000	3.375358

Adduct $C_{20}H_{10}$−HI (type: $ID_{Cora:cv}$)
Symmetry C_{5v}
Energy MP2 = −7684.862502 au
Standard Orientation

6	0	0.000000	1.201653	-0.669818
6	0	-1.142840	0.371331	-0.669818
6	0	1.142840	0.371331	-0.669818
6	0	-0.706314	-0.972158	-0.669818
6	0	0.706314	-0.972158	-0.669818
6	0	0.000000	2.481763	-1.210113
6	0	-2.360297	0.766907	-1.210113
6	0	2.360297	0.766907	-1.210113
6	0	-1.458744	-2.007788	-1.210113
6	0	1.458744	-2.007788	-1.210113
6	0	-1.302618	2.979238	-1.568476
6	0	-2.430892	2.159498	-1.568476
1	0	-1.405816	3.995369	-1.942449
1	0	-3.365401	2.571647	-1.942449
6	0	1.302618	2.979238	-1.568476
6	0	2.430892	2.159498	-1.568476
1	0	1.405816	3.995369	-1.942449
1	0	3.365401	2.571647	-1.942449
6	0	-3.235955	-0.318228	-1.568476
6	0	-2.804992	-1.644594	-1.568476
1	0	-4.234243	-0.102374	-1.942449
1	0	-3.485748	-2.406003	-1.942449
6	0	3.235955	-0.318228	-1.568476
6	0	2.804992	-1.644594	-1.568476
1	0	4.234243	-0.102374	-1.942449
1	0	3.485748	-2.406003	-1.942449
6	0	-0.697312	-3.175914	-1.568476
1	0	-1.211090	-4.058639	-1.942449
6	0	0.697312	-3.175914	-1.568476
1	0	1.211090	-4.058639	-1.942449
1	0	0.000000	0.000000	1.562614
53	0	0.000000	0.000000	3.176761

Adduct $C_{20}H_{10}$−F_2 (type: $ID_{Cora:cv}$)
Symmetry C_{5v}
Energy MP2 = −965.465926 au
Standard Orientation

6	0	0.000000	1.201754	0.142015
6	0	-1.142936	0.371362	0.142015
6	0	1.142936	0.371362	0.142015

6	0	-0.706373	-0.972239	0.142015
6	0	0.706373	-0.972239	0.142015
6	0	0.000000	2.480176	-0.401782
6	0	-2.358788	0.766417	-0.401782
6	0	2.358788	0.766417	-0.401782
6	0	-1.457811	-2.006505	-0.401782
6	0	1.457811	-2.006505	-0.401782
6	0	-1.301990	2.977825	-0.763193
6	0	-2.429743	2.158465	-0.763193
1	0	-1.403978	3.993169	-1.139809
1	0	-3.363876	2.569219	-1.139809
6	0	1.301990	2.977825	-0.763193
6	0	2.429743	2.158465	-0.763193
1	0	1.403978	3.993169	-1.139809
1	0	3.363876	2.569219	-1.139809
6	0	-3.234417	-0.318068	-0.763193
6	0	-2.803654	-1.643821	-0.763193
1	0	-4.231582	-0.101305	-1.139809
1	0	-3.482968	-2.405304	-1.139809
6	0	3.234417	-0.318068	-0.763193
6	0	2.803654	-1.643821	-0.763193
1	0	4.231582	-0.101305	-1.139809
1	0	3.482968	-2.405304	-1.139809
6	0	-0.696990	-3.174402	-0.763193
1	0	-1.211284	-4.055779	-1.139809
6	0	0.696990	-3.174402	-0.763193
1	0	1.211284	-4.055779	-1.139809
9	0	0.000000	0.000000	2.908590
9	0	0.000000	0.000000	4.311707

Adduct $C_{20}H_{10}$−Cl_2 (type: $ID_{Cora:cv}$)
Symmetry C_{5v}
Energy MP2 = −1685.562745 au
Standard Orientation

6	0	0.000000	1.201653	-0.316103
6	0	-1.142840	0.371331	-0.316103
6	0	1.142840	0.371331	-0.316103
6	0	-0.706314	-0.972158	-0.316103
6	0	0.706314	-0.972158	-0.316103
6	0	0.000000	2.481548	-0.856292
6	0	-2.360092	0.766841	-0.856292
6	0	2.360092	0.766841	-0.856292
6	0	-1.458617	-2.007615	-0.856292
6	0	1.458617	-2.007615	-0.856292
6	0	-1.302685	2.979132	-1.215089
6	0	-2.430771	2.159530	-1.215089
1	0	-1.405756	3.995292	-1.589148
1	0	-3.365346	2.571567	-1.589148
6	0	1.302685	2.979132	-1.215089
6	0	2.430771	2.159530	-1.215089
1	0	1.405756	3.995292	-1.589148
1	0	3.365346	2.571567	-1.589148

6	0	-3.235875	-0.318325	-1.215089
6	0	-2.804984	-1.644469	-1.215089
1	0	-4.234151	-0.102340	-1.589148
1	0	-3.485654	-2.405976	-1.589148
6	0	3.235875	-0.318325	-1.215089
6	0	2.804984	-1.644469	-1.215089
1	0	4.234151	-0.102340	-1.589148
1	0	3.485654	-2.405976	-1.589148
6	0	-0.697195	-3.175867	-1.215089
1	0	-1.211093	-4.058542	-1.589148
6	0	0.697195	-3.175867	-1.215089
1	0	1.211093	-4.058542	-1.589148
17	0	0.000000	0.000000	2.650083
17	0	0.000000	0.000000	4.642191

Adduct $C_{20}H_{10}$−Br_2 (type: $ID_{Cora:cv}$)
Symmetry C_{5v}
Energy MP2 = −5911.625030 au
Standard Orientation

6	0	0.000000	1.201651	-1.054572
6	0	-1.142838	0.371331	-1.054572
6	0	1.142838	0.371331	-1.054572
6	0	-0.706313	-0.972156	-1.054572
6	0	0.706313	-0.972156	-1.054572
6	0	0.000000	2.482470	-1.592398
6	0	-2.360969	0.767125	-1.592398
6	0	2.360969	0.767125	-1.592398
6	0	-1.459159	-2.008360	-1.592398
6	0	1.459159	-2.008360	-1.592398
6	0	-1.303203	2.980065	-1.949443
6	0	-2.431498	2.160310	-1.949443
1	0	-1.407028	3.996756	-2.321834
1	0	-3.366345	2.573229	-2.321834
6	0	1.303203	2.980065	-1.949443
6	0	2.431498	2.160310	-1.949443
1	0	1.407028	3.996756	-2.321834
1	0	3.366345	2.573229	-2.321834
6	0	-3.236922	-0.318529	-1.949443
6	0	-2.805952	-1.644920	-1.949443
1	0	-4.235936	-0.103098	-2.321834
1	0	-3.487544	-2.406413	-2.321834
6	0	3.236922	-0.318529	-1.949443
6	0	2.805952	-1.644920	-1.949443
1	0	4.235936	-0.103098	-2.321834
1	0	3.487544	-2.406413	-2.321834
6	0	-0.697325	-3.176927	-1.949443
1	0	-1.210925	-4.060474	-2.321834
6	0	0.697325	-3.176927	-1.949443
1	0	1.210925	-4.060474	-2.321834
35	0	0.000000	0.000000	1.997520
35	0	0.000000	0.000000	4.276595

Adduct $C_{20}H_{10}$−I_2 (type: $ID_{Cora:cv}$)
Symmetry C_{5v}
Energy MP2 = −14602.362851 au
Standard Orientation

6	0	0.000000	1.201482	-1.668994
6	0	-1.142677	0.371278	-1.668994
6	0	1.142677	0.371278	-1.668994
6	0	-0.706213	-0.972019	-1.668994
6	0	0.706213	-0.972019	-1.668994
6	0	0.000000	2.482890	-2.204835
6	0	-2.361369	0.767255	-2.204835
6	0	2.361369	0.767255	-2.204835
6	0	-1.459406	-2.008700	-2.204835
6	0	1.459406	-2.008700	-2.204835
6	0	-1.303661	2.980956	-2.559405
6	0	-2.432204	2.161021	-2.559405
1	0	-1.408183	3.998257	-2.929924
1	0	-3.367416	2.574791	-2.929924
6	0	1.303661	2.980956	-2.559405
6	0	2.432204	2.161021	-2.559405
1	0	1.408183	3.998257	-2.929924
1	0	3.367416	2.574791	-2.929924
6	0	-3.237911	-0.318689	-2.559405
6	0	-2.806846	-1.645371	-2.559405
1	0	-4.237721	-0.103732	-2.929924
1	0	-3.489360	-2.406949	-2.929924
6	0	3.237911	-0.318689	-2.559405
6	0	2.806846	-1.645371	-2.559405
1	0	4.237721	-0.103732	-2.929924
1	0	3.489360	-2.406949	-2.929924
6	0	-0.697478	-3.177917	-2.559405
1	0	-1.210873	-4.062367	-2.929924
6	0	0.697478	-3.177917	-2.559405
1	0	1.210873	-4.062367	-2.929924
53	0	0.000000	0.000000	1.487615
53	0	0.000000	0.000000	4.155374

Adduct $C_{20}H_{10}$−ClF (type: $ID_{Cora:cv}$)
Symmetry C_{5v}
Energy MP2 = −1325.540965 au
Standard Orientation

6	0	0.000000	1.201784	-0.061925
6	0	-1.142964	0.371372	-0.061925
6	0	1.142964	0.371372	-0.061925
6	0	-0.706391	-0.972264	-0.061925
6	0	0.706391	-0.972264	-0.061925
6	0	0.000000	2.482245	-0.600754
6	0	-2.360755	0.767056	-0.600754
6	0	2.360755	0.767056	-0.600754
6	0	-1.459027	-2.008178	-0.600754
6	0	1.459027	-2.008178	-0.600754

6	0	-1.302909	2.979536	-0.959064
6	0	-2.431086	2.159867	-0.959064
1	0	-1.406323	3.995789	-1.332672
1	0	-3.365643	2.572259	-1.332672
6	0	1.302909	2.979536	-0.959064
6	0	2.431086	2.159867	-0.959064
1	0	1.406323	3.995789	-1.332672
1	0	3.365643	2.572259	-1.332672
6	0	-3.236328	-0.318413	-0.959064
6	0	-2.805403	-1.644664	-0.959064
1	0	-4.234799	-0.102726	-1.332672
1	0	-3.486405	-2.406045	-1.332672
6	0	3.236328	-0.318413	-0.959064
6	0	2.805403	-1.644664	-0.959064
1	0	4.234799	-0.102726	-1.332672
1	0	3.486405	-2.406045	-1.332672
6	0	-0.697252	-3.176326	-0.959064
1	0	-1.210927	-4.059277	-1.332672
6	0	0.697252	-3.176326	-0.959064
1	0	1.210927	-4.059277	-1.332672
17	0	0.000000	0.000000	2.921286
9	0	0.000000	0.000000	4.565450

Adduct $C_{20}H_{10}$−BrF (type: $ID_{Cora:cv}$)
Symmetry C_{5v}
Energy MP2 = −3438.580448 au
Standard Orientation

6	0	0.000000	1.201861	-0.385041
6	0	-1.143038	0.371395	-0.385041
6	0	1.143038	0.371395	-0.385041
6	0	-0.706436	-0.972326	-0.385041
6	0	0.706436	-0.972326	-0.385041
6	0	0.000000	2.483138	-0.921635
6	0	-2.361605	0.767332	-0.921635
6	0	2.361605	0.767332	-0.921635
6	0	-1.459552	-2.008901	-0.921635
6	0	1.459552	-2.008901	-0.921635
6	0	-1.303421	2.980473	-1.277965
6	0	-2.431819	2.160644	-1.277965
1	0	-1.407598	3.997266	-1.649822
1	0	-3.366654	2.573928	-1.649822
6	0	1.303421	2.980473	-1.277965
6	0	2.431819	2.160644	-1.277965
1	0	1.407598	3.997266	-1.649822
1	0	3.366654	2.573928	-1.649822
6	0	-3.237378	-0.318610	-1.277965
6	0	-2.806368	-1.645122	-1.277965
1	0	-4.236598	-0.103482	-1.649822
1	0	-3.488305	-2.406491	-1.649822
6	0	3.237378	-0.318610	-1.277965
6	0	2.806368	-1.645122	-1.277965
1	0	4.236598	-0.103482	-1.649822

1	0	3.488305	-2.406491	-1.649822
6	0	-0.697388	-3.177385	-1.277965
1	0	-1.210763	-4.061222	-1.649822
6	0	0.697388	-3.177385	-1.277965
1	0	1.210763	-4.061222	-1.649822
35	0	0.000000	0.000000	2.644815
9	0	0.000000	0.000000	4.423099

Adduct $C_{20}H_{10}$−IF (type: $ID_{Cora:cv}$)
Symmetry C_{5v}
Energy MP2 = −7783.976074 au
Standard Orientation

6	0	-0.706877	0.972932	-1.510342
6	0	0.706877	0.972932	-1.510342
6	0	-1.143750	-0.371627	-1.510342
6	0	1.143750	-0.371627	-1.510342
6	0	0.000000	-1.202610	-1.510342
6	0	-1.460075	2.009621	-0.970695
6	0	1.460075	2.009621	-0.970695
6	0	-2.362451	-0.767607	-0.970695
6	0	2.362451	-0.767607	-0.970695
6	0	0.000000	-2.484028	-0.970695
6	0	-0.697631	3.176193	-0.611120
6	0	0.697631	3.176193	-0.611120
1	0	-1.210606	4.056992	-0.231715
1	0	1.210606	4.056992	-0.231715
6	0	-2.805159	1.644984	-0.611120
6	0	-3.236319	0.318011	-0.611120
1	0	-3.484331	2.405034	-0.231715
1	0	-4.232527	0.102325	-0.231715
6	0	2.805159	1.644984	-0.611120
6	0	3.236319	0.318011	-0.611120
1	0	3.484331	2.405034	-0.231715
1	0	4.232527	0.102325	-0.231715
6	0	-2.431315	-2.159537	-0.611120
6	0	-1.302524	-2.979651	-0.611120
1	0	-3.364041	-2.570599	-0.231715
1	0	-1.405239	-3.993752	-0.231715
6	0	2.431315	-2.159537	-0.611120
1	0	3.364041	-2.570599	-0.231715
6	0	1.302524	-2.979651	-0.611120
1	0	1.405239	-3.993752	-0.231715
53	0	0.000000	0.000000	1.548418
9	0	0.000000	0.000000	3.483260

Adduct $C_{20}H_{10}$−HF (type: $IA_{Cora:cc}$)
Symmetry C_1
Energy MP2 = −866.530331 au
Standard Orientation

6	0	1.177382	-0.000742	-0.822699
6	0	0.346302	1.144700	-0.820370

6	0	-0.997605	0.707225	-0.811100
6	0	-0.998486	-0.705992	-0.811096
6	0	0.344872	-1.145145	-0.820365
6	0	2.461543	-0.001542	-0.283677
6	0	2.961283	1.299806	0.077942
6	0	2.140925	2.426869	0.083769
1	0	3.976594	1.399097	0.454604
1	0	2.550842	3.358128	0.467811
6	0	0.747498	2.357630	-0.270543
6	0	-0.330627	3.232118	0.108315
6	0	-1.654886	2.799635	0.122310
1	0	-0.107773	4.224842	0.492289
1	0	-2.411481	3.472825	0.518855
6	0	-2.021055	1.456612	-0.242337
6	0	-3.182504	0.698601	0.139745
6	0	-3.183374	-0.694636	0.139749
1	0	-4.053163	1.214383	0.538134
1	0	-4.054676	-1.209328	0.538141
6	0	-2.022871	-1.454099	-0.242328
6	0	-1.658380	-2.797575	0.122326
6	0	-0.334661	-3.231710	0.108334
1	0	-2.415815	-3.469818	0.518874
1	0	-0.113047	-4.224709	0.492314
6	0	0.744554	-2.358571	-0.270529
6	0	2.137894	-2.429548	0.083782
6	0	2.959659	-1.303510	0.077949
1	0	2.546648	-3.361316	0.467829
1	0	3.974845	-1.404067	0.454610
1	0	0.605676	-0.000292	1.360798
9	0	0.186287	-0.000057	2.186743

Adduct $C_{20}H_{10}$−HCl (type: $IIA_{Cora:cc}$)
Symmetry C_s
Energy MP2 = −1226.507161 au
Standard Orientation

6	0	0.368869	-0.971800	1.145042
6	0	1.200661	-0.968781	0.000000
6	0	0.368869	-0.971800	-1.145042
6	0	-0.974756	-0.971963	-0.706729
6	0	-0.974756	-0.971963	0.706729
6	0	0.761333	-0.404979	2.353827
6	0	2.146778	-0.024177	2.424370
6	0	2.967519	-0.021133	1.298366
1	0	2.546983	0.373441	3.354309
1	0	3.975467	0.376246	1.393588
6	0	2.473355	-0.400295	0.000000
6	0	2.967519	-0.021133	-1.298366
6	0	2.146778	-0.024177	-2.424370
1	0	3.975467	0.376246	-1.393588
1	0	2.546983	0.373441	-3.354309
6	0	0.761333	-0.404979	-2.353827
6	0	-0.317972	-0.028361	-3.226415

6	0	-1.641450	-0.027298	-2.793909
1	0	-0.096214	0.365503	-4.215622
1	0	-2.402549	0.368319	-3.462742
6	0	-2.001210	-0.402634	-1.453124
6	0	-3.163089	-0.022898	-0.696362
6	0	-3.163089	-0.022898	0.696362
1	0	-4.034060	0.373903	-1.213167
1	0	-4.034060	0.373903	1.213167
6	0	-2.001210	-0.402634	1.453124
6	0	-1.641450	-0.027298	2.793909
6	0	-0.317972	-0.028361	3.226415
1	0	-2.402549	0.368319	3.462742
1	0	-0.096214	0.365503	4.215622
1	0	0.891023	1.328295	0.000000
17	0	-0.039215	2.216133	0.000000

Adduct $C_{20}H_{10}$−HBr (type: $IIA_{Cora:cc}$)
Symmetry C_1
Energy MP2 = −3339.522197 au
Standard Orientation

6	0	-0.399026	1.144750	-1.226538
6	0	-1.230510	-0.000076	-1.212615
6	0	-0.398877	-1.144794	-1.226541
6	0	0.944404	-0.706623	-1.245935
6	0	0.944312	0.706754	-1.245932
6	0	-0.783573	2.353708	-0.654770
6	0	-2.164295	2.424457	-0.256883
6	0	-2.985459	1.298613	-0.243974
1	0	-2.559691	3.354552	0.145207
1	0	-3.988656	1.394456	0.165169
6	0	-2.496231	-0.000160	-0.628553
6	0	-2.985289	-1.298996	-0.243977
6	0	-2.163979	-2.424735	-0.256891
1	0	-3.988474	-1.394971	0.165166
1	0	-2.559254	-3.354882	0.145197
6	0	-0.783266	-2.353806	-0.654779
6	0	0.301744	-3.226505	-0.295017
6	0	1.625511	-2.794437	-0.314198
1	0	0.086098	-4.216038	0.101461
1	0	2.392303	-3.464173	0.068743
6	0	1.980014	-1.453435	-0.693954
6	0	3.148056	-0.696298	-0.334057
6	0	3.147965	0.696713	-0.334054
1	0	4.026057	-1.212817	0.047412
1	0	4.025898	1.213345	0.047416
6	0	1.979824	1.453700	-0.693949
6	0	1.625146	2.794653	-0.314188
6	0	0.301323	3.226549	-0.295005
1	0	2.391851	3.464489	0.068755
1	0	0.085548	4.216051	0.101477
1	0	-0.940920	-0.000074	1.082603
35	0	0.096642	-0.000003	2.059778

Adduct C$_{20}$H$_{10}$−HI (type: IIA$_{Cora:cc}$)
Symmetry C_1
Energy MP2 = −7684.856905 au
Standard Orientation

6	0	-0.475235	1.143883	-1.451983
6	0	-1.306017	0.000111	-1.411035
6	0	-0.475434	-1.143808	-1.451976
6	0	0.866333	-0.706692	-1.522770
6	0	0.866455	0.706533	-1.522774
6	0	-0.837251	2.352517	-0.865301
6	0	-2.203840	2.424531	-0.420935
6	0	-3.025641	1.299408	-0.382987
1	0	-2.585235	3.354723	-0.005747
1	0	-4.015205	1.396383	0.058063
6	0	-2.550633	0.000220	-0.784623
6	0	-3.025867	-1.298883	-0.382981
6	0	-2.204263	-2.424151	-0.420927
1	0	-4.015447	-1.395685	0.058071
1	0	-2.585821	-3.354275	-0.005735
6	0	-0.837661	-2.352377	-0.865293
6	0	0.260836	-3.225716	-0.549505
6	0	1.583697	-2.795481	-0.623010
1	0	0.061006	-4.215439	-0.145319
1	0	2.364496	-3.467125	-0.273191
6	0	1.923890	-1.454205	-1.015499
6	0	3.107556	-0.696957	-0.709238
6	0	3.107677	0.696411	-0.709241
1	0	4.002366	-1.213226	-0.368682
1	0	4.002578	1.212526	-0.368688
6	0	1.924143	1.453865	-1.015506
6	0	1.584184	2.795201	-0.623024
6	0	0.261398	3.225666	-0.549519
1	0	2.365100	3.466712	-0.273209
1	0	0.061741	4.215425	-0.145337
1	0	-1.100911	-0.000072	0.995081
53	0	0.192064	-0.000007	1.964971

Adduct C$_{20}$H$_{10}$−F$_2$ (type: IIA$_{Cora:cc}$)
Symmetry C_s
Energy MP2 = −965.471209 au
Standard Orientation

6	0	-0.949396	-0.956746	0.706815
6	0	-0.949396	-0.956746	-0.706815
6	0	-0.938510	0.387866	-1.143211
6	0	-0.936311	1.219477	0.000000
6	0	-0.938510	0.387866	1.143211
6	0	-0.396925	-1.990285	1.455047
6	0	-0.026973	-3.155435	0.696829
6	0	-0.026973	-3.155435	-0.696829
1	0	0.356998	-4.032769	1.212949

1	0	0.356998	-4.032769	-1.212949
6	0	-0.396925	-1.990285	-1.455047
6	0	-0.018191	-1.632249	-2.796007
6	0	-0.005149	-0.307407	-3.225251
1	0	0.364345	-2.396849	-3.468590
1	0	0.387477	-0.089396	-4.215999
6	0	-0.370708	0.776033	-2.351781
6	0	0.014173	2.160232	-2.423212
6	0	0.017264	2.979841	-1.296910
1	0	0.409326	2.560712	-3.354222
1	0	0.413182	3.988546	-1.393166
6	0	-0.365045	2.487235	0.000000
6	0	0.017264	2.979841	1.296910
6	0	0.014173	2.160232	2.423212
1	0	0.413182	3.988546	1.393166
1	0	0.409326	2.560712	3.354222
6	0	-0.370708	0.776033	2.351781
6	0	-0.005149	-0.307407	3.225251
6	0	-0.018191	-1.632249	2.796007
1	0	0.387477	-0.089396	4.215999
1	0	0.364345	-2.396849	3.468590
9	0	2.037817	-0.781484	0.000000
9	0	1.966456	0.621156	0.000000

Adduct $C_{20}H_{10}-Cl_2$ (type: $IIA_{Cora:cc}$)
Symmetry C_1
Energy MP2 = −1685.573434 au
Standard Orientation

6	0	0.425914	-1.141603	-1.183148
6	0	1.258755	0.000257	-1.176968
6	0	0.425504	1.141818	-1.183110
6	0	-0.917866	0.706305	-1.220367
6	0	-0.917611	-0.706571	-1.220384
6	0	0.804050	-2.348538	-0.604786
6	0	2.185215	-2.421094	-0.210295
6	0	3.010397	-1.297094	-0.211535
1	0	2.580958	-3.350009	0.194521
1	0	4.017692	-1.396343	0.187779
6	0	2.524350	0.000476	-0.601834
6	0	3.009931	1.298206	-0.211491
6	0	2.184342	2.421907	-0.210208
1	0	4.017189	1.397804	0.187829
1	0	2.579751	3.350951	0.194640
6	0	0.803203	2.348866	-0.604704
6	0	-0.289597	3.218529	-0.255684
6	0	-1.615964	2.793317	-0.307874
1	0	-0.080922	4.206173	0.149570
1	0	-2.387808	3.467035	0.057810
6	0	-1.968994	1.456207	-0.705213
6	0	-3.148797	0.697520	-0.386082
6	0	-3.148543	-0.698607	-0.386095
1	0	-4.043219	1.212209	-0.041024

1	0	-4.042778	-1.213627	-0.041041
6	0	-1.968468	-1.456862	-0.705241
6	0	-1.614955	-2.793855	-0.307937
6	0	-0.288438	-3.218598	-0.255777
1	0	-2.386556	-3.467857	0.057740
1	0	-0.079405	-4.206179	0.149448
17	0	0.856762	-0.000124	2.037301
17	0	-1.132613	-0.000092	2.115353

Adduct $C_{20}H_{10}$−Br_2 (type: $IIB_{Cora:cc}$)
Symmetry C_s
Energy MP2 = −5911.636107 au
Standard Orientation

6	0	0.851325	1.934139	0.707915
6	0	0.851325	1.934139	-0.707915
6	0	1.549934	0.785006	-1.144820
6	0	1.976284	0.072798	0.000000
6	0	1.549934	0.785006	1.144820
6	0	-0.174190	2.511112	1.453947
6	0	-1.110509	3.297921	0.696821
6	0	-1.110509	3.297921	-0.696821
1	0	-1.906529	3.829154	1.214133
1	0	-1.906529	3.829154	-1.214133
6	0	-0.174190	2.511112	-1.453947
6	0	-0.313328	2.006968	-2.793318
6	0	0.374084	0.875000	-3.222351
1	0	-1.049357	2.450681	-3.460096
1	0	0.148569	0.475765	-4.208799
6	0	1.258296	0.151357	-2.349854
6	0	1.641750	-1.232209	-2.420695
6	0	2.061725	-1.933527	-1.293410
1	0	1.503385	-1.780619	-3.349905
1	0	2.236279	-3.003397	-1.384294
6	0	2.134848	-1.310657	0.000000
6	0	2.061725	-1.933527	1.293410
6	0	1.641750	-1.232209	2.420695
1	0	2.236279	-3.003397	1.384294
1	0	1.503385	-1.780619	3.349905
6	0	1.258296	0.151357	2.349854
6	0	0.374084	0.875000	3.222351
6	0	-0.313328	2.006968	2.793318
1	0	0.148569	0.475765	4.208799
1	0	-1.049357	2.450681	3.460096
35	0	-1.389091	-0.252955	0.000000
35	0	-1.473781	-2.526051	0.000000

Adduct $C_{20}H_{10}$−I_2 (type: $IIB_{Cora:cc}$)
Symmetry C_s
Energy MP2 = −14602.374392 au
Standard Orientation

6	0	-0.597905	-2.453408	0.707783

6	0	-0.597905	-2.453408	-0.707783
6	0	-1.522046	-1.476828	-1.144847
6	0	-2.086336	-0.868416	0.000000
6	0	-1.522046	-1.476828	1.144847
6	0	0.525786	-2.802568	1.454478
6	0	1.607508	-3.373102	0.697016
6	0	1.607508	-3.373102	-0.697016
1	0	2.497002	-3.726245	1.214350
1	0	2.497002	-3.726245	-1.214350
6	0	0.525786	-2.802568	-1.454478
6	0	0.551473	-2.287240	-2.796679
6	0	-0.358666	-1.325285	-3.226173
1	0	1.361255	-2.570561	-3.465372
1	0	-0.225296	-0.891987	-4.214994
6	0	-1.369801	-0.797397	-2.351061
6	0	-2.030965	0.476854	-2.421669
6	0	-2.584222	1.077520	-1.293511
1	0	-2.010143	1.041632	-3.351148
1	0	-2.974694	2.088606	-1.384039
6	0	-2.525512	0.453406	0.000000
6	0	-2.584222	1.077520	1.293511
6	0	-2.030965	0.476854	2.421669
1	0	-2.974694	2.088606	1.384039
1	0	-2.010143	1.041632	3.351148
6	0	-1.369801	-0.797397	2.351061
6	0	-0.358666	-1.325285	3.226173
6	0	0.551473	-2.287240	2.796679
1	0	-0.225296	-0.891987	4.214994
1	0	1.361255	-2.570561	3.465372
53	0	1.223634	0.268411	0.000000
53	0	0.657892	2.866393	0.000000

Adduct $C_{20}H_{10}$−ClF (type: $IA_{Cora:cc}$)
Symmetry C_1
Energy MP2 = −1325.548825 au
Standard Orientation

6	0	0.344580	-1.145295	-1.112144
6	0	1.174601	-0.000177	-1.097537
6	0	0.344937	1.145201	-1.112131
6	0	-0.998962	0.707323	-1.119438
6	0	-0.999182	-0.706999	-1.119445
6	0	0.743290	-2.358801	-0.561732
6	0	2.133827	-2.428658	-0.198096
6	0	2.954860	-1.301816	-0.196046
1	0	2.541042	-3.361694	0.184993
1	0	3.968395	-1.402868	0.185301
6	0	2.457007	-0.000379	-0.554806
6	0	2.955266	1.300901	-0.196037
6	0	2.134584	2.428000	-0.198078
1	0	3.968834	1.401635	0.185310
1	0	2.542092	3.360905	0.185018
6	0	0.744027	2.358577	-0.561711

6	0	-0.335252	3.230721	-0.183748
6	0	-1.659064	2.795692	-0.174016
1	0	-0.114941	4.222803	0.203906
1	0	-2.418170	3.465707	0.223566
6	0	-2.023269	1.454748	-0.548051
6	0	-3.184854	0.697087	-0.165816
6	0	-3.185072	-0.696087	-0.165822
1	0	-4.055597	1.213189	0.232401
1	0	-4.055974	-1.211922	0.232392
6	0	-2.023722	-1.454108	-0.548064
6	0	-1.659936	-2.795168	-0.174039
6	0	-0.336261	-3.230611	-0.183774
1	0	-2.419251	-3.464950	0.223539
1	0	-0.116258	-4.222764	0.203873
17	0	0.299535	-0.000043	1.732302
9	0	-0.268967	-0.000022	3.279307

Adduct $C_{20}H_{10}$−BrF (type: $IA_{Cora:cc}$)
Symmetry C_1
Energy MP2 = −3438.588695 au
Standard Orientation

6	0	0.280292	-1.145334	-1.321542
6	0	1.110233	-0.000246	-1.325519
6	0	0.280789	1.145204	-1.321536
6	0	-1.062711	0.707365	-1.296292
6	0	-1.063018	-0.706911	-1.296294
6	0	0.694968	-2.361564	-0.789316
6	0	2.096562	-2.431407	-0.469303
6	0	2.918083	-1.304240	-0.488275
1	0	2.516289	-3.365579	-0.102979
1	0	3.943108	-1.408058	-0.139973
6	0	2.410211	-0.000528	-0.825797
6	0	2.918651	1.302964	-0.488278
6	0	2.097621	2.430490	-0.469306
1	0	3.943721	1.406336	-0.139978
1	0	2.517757	3.364480	-0.102985
6	0	0.695996	2.361257	-0.789314
6	0	-0.373714	3.234004	-0.386101
6	0	-1.696855	2.797655	-0.339170
1	0	-0.144601	4.227726	-0.008009
1	0	-2.444775	3.468855	0.077114
6	0	-2.071818	1.455815	-0.700045
6	0	-3.222834	0.697494	-0.287843
6	0	-3.223137	-0.696100	-0.287844
1	0	-4.083184	1.213023	0.132986
1	0	-4.083711	-1.211253	0.132986
6	0	-2.072451	-1.454920	-0.700046
6	0	-1.698074	-2.796922	-0.339171
6	0	-0.375122	-3.233847	-0.386102
1	0	-2.446284	-3.467799	0.077113
1	0	-0.146440	-4.227667	-0.008009
35	0	0.306623	-0.000037	1.605987

9	0	-0.240635	-0.000015	3.301641

Adduct $C_{20}H_{10}$−IF (type: $IA_{Cora:cc}$)
Symmetry C_s
Energy MP2 = −7783.976219 au
Standard Orientation

6	0	-1.522510	-0.211189	1.144448
6	0	-1.556276	-1.040166	0.000000
6	0	-1.522510	-0.211189	-1.144448
6	0	-1.453247	1.130437	-0.706858
6	0	-1.453247	1.130437	0.706858
6	0	-1.015588	-0.644958	2.364504
6	0	-0.753781	-2.059129	2.433928
6	0	-0.802596	-2.879851	1.306011
1	0	-0.409923	-2.495022	3.369152
1	0	-0.498384	-3.918440	1.412881
6	0	-1.114062	-2.360109	0.000000
6	0	-0.802596	-2.879851	-1.306011
6	0	-0.753781	-2.059129	-2.433928
1	0	-0.498384	-3.918440	-1.412881
1	0	-0.409923	-2.495022	-3.369152
6	0	-1.015588	-0.644958	-2.364504
6	0	-0.584310	0.413103	-3.238708
6	0	-0.491686	1.734499	-2.801690
1	0	-0.221487	0.174193	-4.235743
1	0	-0.056932	2.468257	-3.476729
6	0	-0.831335	2.121831	-1.457360
6	0	-0.386277	3.259792	-0.697200
6	0	-0.386277	3.259792	0.697200
1	0	0.058078	4.109050	-1.211313
1	0	0.058078	4.109050	1.211313
6	0	-0.831335	2.121831	1.457360
6	0	-0.491686	1.734499	2.801690
6	0	-0.584310	0.413103	3.238708
1	0	-0.056932	2.468257	3.476729
1	0	-0.221487	0.174193	4.235743
53	0	1.538023	-0.296477	0.000000
9	0	3.428896	0.118270	0.000000

Adduct $C_{20}H_{10}$−HF (type: $ID_{Cora:cc}$)
Symmetry C_{5v}
Energy MP2 = −866.529965 au
Standard Orientation

6	0	-0.707284	0.973493	-0.820520
6	0	0.707284	0.973493	-0.820520
6	0	-1.144410	-0.371841	-0.820520
6	0	1.144410	-0.371841	-0.820520
6	0	0.000000	-1.203303	-0.820520
6	0	-1.457025	2.005423	-0.265479
6	0	1.457025	2.005423	-0.265479
6	0	-2.357516	-0.766003	-0.265479

6	0	2.357516	-0.766003	-0.265479
6	0	0.000000	-2.478839	-0.265479
6	0	-0.696845	3.170753	0.101924
6	0	0.696845	3.170753	0.101924
1	0	-1.211442	4.046635	0.490124
1	0	1.211442	4.046635	0.490124
6	0	-2.800228	1.642556	0.101924
6	0	-3.230902	0.317078	0.101924
1	0	-3.474222	2.402629	0.490124
1	0	-4.222935	0.098329	0.490124
6	0	2.800228	1.642556	0.101924
6	0	3.230902	0.317078	0.101924
1	0	3.474222	2.402629	0.490124
1	0	4.222935	0.098329	0.490124
6	0	-2.427481	-2.155598	0.101924
6	0	-1.299962	-2.974788	0.101924
1	0	-3.358629	-2.561729	0.490124
1	0	-1.398475	-3.985864	0.490124
6	0	2.427481	-2.155598	0.101924
1	0	3.358629	-2.561729	0.490124
6	0	1.299962	-2.974788	0.101924
1	0	1.398475	-3.985864	0.490124
1	0	0.000000	0.000000	1.323893
9	0	0.000000	0.000000	2.248823

Adduct $C_{20}H_{10}$−HCl (type: $ID_{Cora:cc}$)
Symmetry C_{5v}
Energy MP2 = −1226.506152 au
Standard Orientation

6	0	-0.707529	0.973830	-0.984795
6	0	0.707529	0.973830	-0.984795
6	0	-1.144806	-0.371970	-0.984795
6	0	1.144806	-0.371970	-0.984795
6	0	0.000000	-1.203720	-0.984795
6	0	-1.455089	2.002759	-0.418899
6	0	1.455089	2.002759	-0.418899
6	0	-2.354384	-0.764986	-0.418899
6	0	2.354384	-0.764986	-0.418899
6	0	0.000000	-2.475546	-0.418899
6	0	-0.696622	3.165498	-0.043202
6	0	0.696622	3.165498	-0.043202
1	0	-1.212423	4.037030	0.353315
1	0	1.212423	4.037030	0.353315
6	0	-2.795300	1.640720	-0.043202
6	0	-3.225836	0.315666	-0.043202
1	0	-3.464784	2.400594	0.353315
1	0	-4.214103	0.094428	0.353315
6	0	2.795300	1.640720	-0.043202
6	0	3.225836	0.315666	-0.043202
1	0	3.464784	2.400594	0.353315
1	0	4.214103	0.094428	0.353315
6	0	-2.424212	-2.151478	-0.043202

6	0	-1.297054	-2.970406	-0.043202
1	0	-3.353778	-2.553382	0.353315
1	0	-1.392036	-3.978670	0.353315
6	0	2.424212	-2.151478	-0.043202
1	0	3.353778	-2.553382	0.353315
6	0	1.297054	-2.970406	-0.043202
1	0	1.392036	-3.978670	0.353315
1	0	0.000000	0.000000	1.075066
17	0	0.000000	0.000000	2.358513

Adduct $C_{20}H_{10}$−HBr (type: $ID_{Cora:cc}$)
Symmetry C_{5v}
Energy MP2 = −3339.520974 au
Standard Orientation

6	0	-0.707532	0.973835	-1.269030
6	0	0.707532	0.973835	-1.269030
6	0	-1.144811	-0.371972	-1.269030
6	0	1.144811	-0.371972	-1.269030
6	0	0.000000	-1.203726	-1.269030
6	0	-1.454930	2.002540	-0.702220
6	0	1.454930	2.002540	-0.702220
6	0	-2.354127	-0.764902	-0.702220
6	0	2.354127	-0.764902	-0.702220
6	0	0.000000	-2.475275	-0.702220
6	0	-0.696643	3.165150	-0.326078
6	0	0.696643	3.165150	-0.326078
1	0	-1.212520	4.036413	0.070986
1	0	1.212520	4.036413	0.070986
6	0	-2.794962	1.640632	-0.326078
6	0	-3.225511	0.315538	-0.326078
1	0	-3.464168	2.400495	0.070986
1	0	-4.213546	0.094145	0.070986
6	0	2.794962	1.640632	-0.326078
6	0	3.225511	0.315538	-0.326078
1	0	3.464168	2.400495	0.070986
1	0	4.213546	0.094145	0.070986
6	0	-2.424024	-2.151184	-0.326078
6	0	-1.296832	-2.970137	-0.326078
1	0	-3.353493	-2.552825	0.070986
1	0	-1.391595	-3.978228	0.070986
6	0	2.424024	-2.151184	-0.326078
1	0	3.353493	-2.552825	0.070986
6	0	1.296832	-2.970137	-0.326078
1	0	1.391595	-3.978228	0.070986
1	0	0.000000	0.000000	0.784324
35	0	0.000000	0.000000	2.205943

Adduct $C_{20}H_{10}$−HI (type: $ID_{Cora:cc}$)
Symmetry C_{5v}
Energy MP2 = −7684.855427 au
Standard Orientation

6	0	-0.707617	0.973951	-1.543018
6	0	0.707617	0.973951	-1.543018
6	0	-1.144948	-0.372016	-1.543018
6	0	1.144948	-0.372016	-1.543018
6	0	0.000000	-1.203870	-1.543018
6	0	-1.453944	2.001183	-0.971365
6	0	1.453944	2.001183	-0.971365
6	0	-2.352531	-0.764384	-0.971365
6	0	2.352531	-0.764384	-0.971365
6	0	0.000000	-2.473598	-0.971365
6	0	-0.696551	3.163213	-0.592329
6	0	0.696551	3.163213	-0.592329
1	0	-1.212861	4.032833	-0.192218
1	0	1.212861	4.032833	-0.192218
6	0	-2.793148	1.639946	-0.592329
6	0	-3.223640	0.315027	-0.592329
1	0	-3.460657	2.399713	-0.192218
1	0	-4.210247	0.092715	-0.192218
6	0	2.793148	1.639946	-0.592329
6	0	3.223640	0.315027	-0.592329
1	0	3.460657	2.399713	-0.192218
1	0	4.210247	0.092715	-0.192218
6	0	-2.422812	-2.149671	-0.592329
6	0	-1.295768	-2.968516	-0.592329
1	0	-3.351665	-2.549729	-0.192218
1	0	-1.389215	-3.975532	-0.192218
6	0	2.422812	-2.149671	-0.592329
1	0	3.351665	-2.549729	-0.192218
6	0	1.295768	-2.968516	-0.592329
1	0	1.389215	-3.975532	-0.192218
1	0	0.000000	0.000000	0.508387
53	0	0.000000	0.000000	2.120473

Adduct $C_{20}H_{10}$−F_2 (type: $ID_{Cora:cc}$)
Symmetry C_{5v}
Energy MP2 = −965.470023 au
Standard Orientation

6	0	-0.707024	0.973136	-0.988404
6	0	0.707024	0.973136	-0.988404
6	0	-1.143990	-0.371705	-0.988404
6	0	1.143990	-0.371705	-0.988404
6	0	0.000000	-1.202862	-0.988404
6	0	-1.454760	2.002306	-0.427078
6	0	1.454760	2.002306	-0.427078
6	0	-2.353852	-0.764813	-0.427078
6	0	2.353852	-0.764813	-0.427078
6	0	0.000000	-2.474986	-0.427078
6	0	-0.696475	3.166374	-0.052989
6	0	0.696475	3.166374	-0.052989
1	0	-1.212526	4.040978	0.336929
1	0	1.212526	4.040978	0.336929
6	0	-2.796178	1.640850	-0.052989

6	0	-3.226623	0.316076	-0.052989
1	0	-3.468507	2.401912	0.336929
1	0	-4.217890	0.095550	0.336929
6	0	2.796178	1.640850	-0.052989
6	0	3.226623	0.316076	-0.052989
1	0	3.468507	2.401912	0.336929
1	0	4.217890	0.095550	0.336929
6	0	-2.424608	-2.152273	-0.052989
6	0	-1.297688	-2.971028	-0.052989
1	0	-3.356181	-2.556515	0.336929
1	0	-1.394273	-3.981925	0.336929
6	0	2.424608	-2.152273	-0.052989
1	0	3.356181	-2.556515	0.336929
6	0	1.297688	-2.971028	-0.052989
1	0	1.394273	-3.981925	0.336929
9	0	0.000000	0.000000	1.646669
9	0	0.000000	0.000000	3.050497

Adduct $C_{20}H_{10}$−Cl_2 (type: $ID_{Cora:cc}$)
Symmetry C_{5v}
Energy MP2 = −1685.572025 au
Standard Orientation

6	0	-0.707420	0.973681	-1.328590
6	0	0.707420	0.973681	-1.328590
6	0	-1.144630	-0.371913	-1.328590
6	0	1.144630	-0.371913	-1.328590
6	0	0.000000	-1.203536	-1.328590
6	0	-1.454307	2.001682	-0.761185
6	0	1.454307	2.001682	-0.761185
6	0	-2.353118	-0.764574	-0.761185
6	0	2.353118	-0.764574	-0.761185
6	0	0.000000	-2.474215	-0.761185
6	0	-0.696523	3.163037	-0.380816
6	0	0.696523	3.163037	-0.380816
1	0	-1.212955	4.033855	0.016969
1	0	1.212955	4.033855	0.016969
6	0	-2.792990	1.639865	-0.380816
6	0	-3.223464	0.314999	-0.380816
1	0	-3.461600	2.400119	0.016969
1	0	-4.211248	0.092941	0.016969
6	0	2.792990	1.639865	-0.380816
6	0	3.223464	0.314999	-0.380816
1	0	3.461600	2.400119	0.016969
1	0	4.211248	0.092941	0.016969
6	0	-2.422685	-2.149545	-0.380816
6	0	-1.295688	-2.968357	-0.380816
1	0	-3.352342	-2.550500	0.016969
1	0	-1.389739	-3.976414	0.016969
6	0	2.422685	-2.149545	-0.380816
1	0	3.352342	-2.550500	0.016969
6	0	1.295688	-2.968357	-0.380816
1	0	1.389739	-3.976414	0.016969

17	0	0.000000	0.000000	1.515252
17	0	0.000000	0.000000	3.506661

Adduct $C_{20}H_{10}$–Br_2 (type: $ID_{Cora:cc}$)
Symmetry C_{5v}
Energy MP2 = −5911.634686 au
Standard Orientation

6	0	-0.707305	0.973522	-1.862987
6	0	0.707305	0.973522	-1.862987
6	0	-1.144444	-0.371852	-1.862987
6	0	1.144444	-0.371852	-1.862987
6	0	0.000000	-1.203339	-1.862987
6	0	-1.455702	2.003601	-1.301661
6	0	1.455702	2.003601	-1.301661
6	0	-2.355375	-0.765308	-1.301661
6	0	2.355375	-0.765308	-1.301661
6	0	0.000000	-2.476587	-1.301661
6	0	-0.696831	3.166213	-0.926157
6	0	0.696831	3.166213	-0.926157
1	0	-1.212463	4.039328	-0.532433
1	0	1.212463	4.039328	-0.532433
6	0	-2.795915	1.641139	-0.926157
6	0	-3.226580	0.315688	-0.926157
1	0	-3.466958	2.401342	-0.532433
1	0	-4.216301	0.095100	-0.532433
6	0	2.795915	1.641139	-0.926157
6	0	3.226580	0.315688	-0.926157
1	0	3.466958	2.401342	-0.532433
1	0	4.216301	0.095100	-0.532433
6	0	-2.424801	-2.151933	-0.926157
6	0	-1.297305	-2.971107	-0.926157
1	0	-3.355161	-2.555217	-0.532433
1	0	-1.393354	-3.980553	-0.532433
6	0	2.424801	-2.151933	-0.926157
1	0	3.355161	-2.555217	-0.532433
6	0	1.297305	-2.971107	-0.926157
1	0	1.393354	-3.980553	-0.532433
35	0	0.000000	0.000000	1.087159
35	0	0.000000	0.000000	3.365218

Adduct $C_{20}H_{10}$–I_2 (type: $ID_{Cora:cc}$)
Symmetry C_{5v}
Energy MP2 = −14602.372629 au
Standard Orientation

6	0	-2.067925	1.266707	1.039695
6	0	-1.921953	0.087200	1.805636
6	0	-0.949872	2.094028	1.294070
6	0	-0.713684	0.185545	2.533388
6	0	-0.112905	1.425833	2.217223
6	0	-2.767088	1.298868	-0.162697
6	0	-2.466186	-1.132536	1.416191

6	0	-0.462364	3.004284	0.361665
6	0	0.024507	-0.929809	2.916360
6	0	1.262937	1.626886	2.264627
6	0	-3.515711	0.105433	-0.454650
6	0	-3.371755	-1.057773	0.300704
1	0	-4.137342	0.066339	-1.346380
1	0	-3.887130	-1.955464	-0.033475
6	0	-2.411250	2.385554	-1.035881
6	0	-1.308649	3.201441	-0.785021
1	0	-2.948729	2.520192	-1.971896
1	0	-1.032265	3.938309	-1.535870
6	0	-1.833092	-2.286169	1.997811
6	0	-0.641521	-2.189183	2.715506
1	0	-2.231229	-3.277456	1.792941
1	0	-0.160126	-3.108881	3.040387
6	0	0.912609	3.382237	0.552865
6	0	1.738010	2.723277	1.463260
1	0	1.358240	4.132880	-0.096045
1	0	2.792892	2.987522	1.486339
6	0	1.413886	-0.668253	3.183145
1	0	2.051891	-1.472066	3.543657
6	0	2.006361	0.554894	2.871349
1	0	3.081692	0.653922	3.001717
53	0	0.366642	-0.321687	-0.565255
53	0	1.675357	-1.469937	-2.582912

Adduct $C_{20}H_{10}$−ClF (type: $ID_{Cora:cc}$)
Symmetry C_{5v}
Energy MP2 = −1325.548534 au
Standard Orientation

6	0	-0.707332	0.973559	-1.121837
6	0	0.707332	0.973559	-1.121837
6	0	-1.144487	-0.371866	-1.121837
6	0	1.144487	-0.371866	-1.121837
6	0	0.000000	-1.203385	-1.121837
6	0	-1.456103	2.004154	-0.563193
6	0	1.456103	2.004154	-0.563193
6	0	-2.356024	-0.765519	-0.563193
6	0	2.356024	-0.765519	-0.563193
6	0	0.000000	-2.477271	-0.563193
6	0	-0.696812	3.167114	-0.188912
6	0	0.696812	3.167114	-0.188912
1	0	-1.212182	4.041095	0.203104
1	0	1.212182	4.041095	0.203104
6	0	-2.796778	1.641400	-0.188912
6	0	-3.227431	0.315984	-0.188912
1	0	-3.468725	2.401621	0.203104
1	0	-4.217895	0.095913	0.203104
6	0	2.796778	1.641400	-0.188912
6	0	3.227431	0.315984	-0.188912
1	0	3.468725	2.401621	0.203104
1	0	4.217895	0.095913	0.203104

6	0	-2.425316	-2.152673	-0.188912
6	0	-1.297850	-2.971825	-0.188912
1	0	-3.355972	-2.556812	0.203104
1	0	-1.394620	-3.981817	0.203104
6	0	2.425316	-2.152673	-0.188912
1	0	3.355972	-2.556812	0.203104
6	0	1.297850	-2.971825	-0.188912
1	0	1.394620	-3.981817	0.203104
17	0	0.000000	0.000000	1.732286
9	0	0.000000	0.000000	3.378414

Adduct $C_{20}H_{10}$−BrF (type: $ID_{Cora:cc}$)
Symmetry C_{5v}
Energy MP2 = −3438.588416 au
Standard Orientation

6	0	-0.707274	0.973479	-1.322304
6	0	0.707274	0.973479	-1.322304
6	0	-1.144393	-0.371836	-1.322304
6	0	1.144393	-0.371836	-1.322304
6	0	0.000000	-1.203286	-1.322304
6	0	-1.457661	2.006298	-0.770252
6	0	1.457661	2.006298	-0.770252
6	0	-2.358544	-0.766337	-0.770252
6	0	2.358544	-0.766337	-0.770252
6	0	0.000000	-2.479920	-0.770252
6	0	-0.697138	3.170282	-0.400654
6	0	0.697138	3.170282	-0.400654
1	0	-1.211595	4.046406	-0.012360
1	0	1.211595	4.046406	-0.012360
6	0	-2.799690	1.642689	-0.400654
6	0	-3.230545	0.316653	-0.400654
1	0	-3.473957	2.402704	-0.012360
1	0	-4.222764	0.098113	-0.012360
6	0	2.799690	1.642689	-0.400654
6	0	3.230545	0.316653	-0.400654
1	0	3.473957	2.402704	-0.012360
1	0	4.222764	0.098113	-0.012360
6	0	-2.427441	-2.155045	-0.400654
6	0	-1.299448	-2.974579	-0.400654
1	0	-3.358619	-2.561453	-0.012360
1	0	-1.398217	-3.985769	-0.012360
6	0	2.427441	-2.155045	-0.400654
1	0	3.358619	-2.561453	-0.012360
6	0	1.299448	-2.974579	-0.400654
1	0	1.398217	-3.985769	-0.012360
35	0	0.000000	0.000000	1.611860
9	0	0.000000	0.000000	3.391606

Adduct $C_{20}H_{10}$−IF (type: $ID_{Cora:cc}$)
Symmetry C_{5v}
Energy MP2 = −7783.968546 au

Standard Orientation

6	0	0.000000	1.201743	-0.663476
6	0	-1.142925	0.371359	-0.663476
6	0	1.142925	0.371359	-0.663476
6	0	-0.706367	-0.972230	-0.663476
6	0	0.706367	-0.972230	-0.663476
6	0	0.000000	2.483952	-1.196974
6	0	-2.362379	0.767583	-1.196974
6	0	2.362379	0.767583	-1.196974
6	0	-1.460030	-2.009559	-1.196974
6	0	1.460030	-2.009559	-1.196974
6	0	-1.304088	2.981715	-1.549972
6	0	-2.432794	2.161662	-1.549972
1	0	-1.409265	3.999344	-1.919192
1	0	-3.368115	2.576156	-1.919192
6	0	1.304088	2.981715	-1.549972
6	0	2.432794	2.161662	-1.549972
1	0	1.409265	3.999344	-1.919192
1	0	3.368115	2.576156	-1.919192
6	0	-3.238765	-0.318861	-1.549972
6	0	-2.807637	-1.645734	-1.549972
1	0	-4.239089	-0.104425	-1.919192
1	0	-3.490875	-2.407192	-1.919192
6	0	3.238765	-0.318861	-1.549972
6	0	2.807637	-1.645734	-1.549972
1	0	4.239089	-0.104425	-1.919192
1	0	3.490875	-2.407192	-1.919192
6	0	-0.697579	-3.178782	-1.549972
1	0	-1.210636	-4.063882	-1.919192
6	0	0.697579	-3.178782	-1.549972
1	0	1.210636	-4.063882	-1.919192
53	0	0.000000	0.000000	2.429003
9	0	0.000000	0.000000	4.362953

Adduct $C_{24}H_{12}$
Symmetry C_i (very close to D_{6h})
Energy MP2 = −919.548821 au
Standard Orientation

6	0	-1.420922	-0.003329	0.000107
6	0	-0.713344	1.228890	0.000073
6	0	0.707578	1.232219	-0.000013
6	0	1.420922	0.003329	-0.000107
6	0	0.713344	-1.228890	-0.000073
6	0	-0.707578	-1.232219	0.000013
6	0	-2.844177	-0.006664	0.000208
6	0	-3.531714	1.234632	0.000284
6	0	-2.846488	2.427926	0.000253
1	0	-4.619189	1.230097	0.000387
1	0	-3.390716	3.369435	0.000288
6	0	-1.427859	2.459797	0.000152
6	0	-0.696634	3.675870	0.000132
6	0	0.679402	3.679094	0.000047

1	0	-1.244299	4.615384	0.000167
1	0	1.222658	4.621164	0.000057
6	0	1.416317	2.466461	-0.000033
6	0	2.835079	2.441238	-0.000135
6	0	3.525890	1.251168	-0.000224
1	0	3.374890	3.385287	-0.000124
1	0	4.613374	1.251729	-0.000326
6	0	2.844177	0.006664	-0.000208
6	0	3.531714	-1.234632	-0.000284
6	0	2.846488	-2.427926	-0.000253
1	0	4.619189	-1.230097	-0.000387
1	0	3.390716	-3.369435	-0.000288
6	0	1.427859	-2.459797	-0.000152
6	0	0.696634	-3.675870	-0.000132
6	0	-0.679402	-3.679094	-0.000047
1	0	1.244299	-4.615384	-0.000167
1	0	-1.222658	-4.621164	-0.000057
6	0	-1.416317	-2.466461	0.000033
6	0	-2.835079	-2.441238	0.000135
6	0	-3.525890	-1.251168	0.000224
1	0	-3.374890	-3.385287	0.000124
1	0	-4.613374	-1.251729	0.000326

Adduct $C_{24}H_{12}$−HF (type: ID_{Cor})
Symmetry C_{6v}
Energy MP2 = −1019.883059 au
Standard Orientation

6	0	0.711034	1.231548	-0.192526
6	0	-0.711034	1.231548	-0.192526
6	0	-1.422069	0.000000	-0.192526
6	0	-0.711034	-1.231548	-0.192526
6	0	0.711034	-1.231548	-0.192526
6	0	1.422069	0.000000	-0.192526
6	0	1.422758	2.464289	-0.184424
6	0	0.688055	3.677810	-0.180389
6	0	-0.688055	3.677810	-0.180389
1	0	1.233158	4.618630	-0.174371
1	0	-1.233158	4.618630	-0.174371
6	0	-1.422758	2.464289	-0.184424
6	0	-2.841049	2.434778	-0.180389
6	0	-3.529104	1.243032	-0.180389
1	0	-3.383272	3.377261	-0.174371
1	0	-4.616430	1.241369	-0.174371
6	0	-2.845516	0.000000	-0.184424
6	0	-3.529104	-1.243032	-0.180389
6	0	-2.841049	-2.434778	-0.180389
1	0	-4.616430	-1.241369	-0.174371
1	0	-3.383272	-3.377261	-0.174371
6	0	-1.422758	-2.464289	-0.184424
6	0	-0.688055	-3.677810	-0.180389
6	0	0.688055	-3.677810	-0.180389
1	0	-1.233158	-4.618630	-0.174371

1	0	1.233158	-4.618630	-0.174371
6	0	1.422758	-2.464289	-0.184424
6	0	2.841049	-2.434778	-0.180389
6	0	3.529104	-1.243032	-0.180389
1	0	3.383272	-3.377261	-0.174371
1	0	4.616430	-1.241369	-0.174371
6	0	2.845516	0.000000	-0.184424
6	0	3.529104	1.243032	-0.180389
6	0	2.841049	2.434778	-0.180389
1	0	4.616430	1.241369	-0.174371
1	0	3.383272	3.377261	-0.174371
1	0	0.000000	0.000000	2.033581
9	0	0.000000	0.000000	2.957455

Adduct $C_{24}H_{12}$−HCl (type: ID_{Cor})
Symmetry C_{6v}
Energy MP2 = −1379.856280 au
Standard Orientation

6	0	0.710861	1.231247	-0.366302
6	0	-0.710861	1.231247	-0.366302
6	0	-1.421722	0.000000	-0.366302
6	0	-0.710861	-1.231247	-0.366302
6	0	0.710861	-1.231247	-0.366302
6	0	1.421722	0.000000	-0.366302
6	0	1.422568	2.463960	-0.349562
6	0	0.688091	3.677565	-0.339642
6	0	-0.688091	3.677565	-0.339642
1	0	1.233292	4.618322	-0.327744
1	0	-1.233292	4.618322	-0.327744
6	0	-1.422568	2.463960	-0.349562
6	0	-2.840819	2.434687	-0.339642
6	0	-3.528910	1.242878	-0.339642
1	0	-3.382938	3.377223	-0.327744
1	0	-4.616230	1.241099	-0.327744
6	0	-2.845136	0.000000	-0.349562
6	0	-3.528910	-1.242878	-0.339642
6	0	-2.840819	-2.434687	-0.339642
1	0	-4.616230	-1.241099	-0.327744
1	0	-3.382938	-3.377223	-0.327744
6	0	-1.422568	-2.463960	-0.349562
6	0	-0.688091	-3.677565	-0.339642
6	0	0.688091	-3.677565	-0.339642
1	0	-1.233292	-4.618322	-0.327744
1	0	1.233292	-4.618322	-0.327744
6	0	1.422568	-2.463960	-0.349562
6	0	2.840819	-2.434687	-0.339642
6	0	3.528910	-1.242878	-0.339642
1	0	3.382938	-3.377223	-0.327744
1	0	4.616230	-1.241099	-0.327744
6	0	2.845136	0.000000	-0.349562
6	0	3.528910	1.242878	-0.339642
6	0	2.840819	2.434687	-0.339642

1	0	4.616230	1.241099	-0.327744
1	0	3.382938	3.377223	-0.327744
1	0	0.000000	0.000000	1.798517
17	0	0.000000	0.000000	3.079984

Adduct $C_{24}H_{12}$−HBr (type: ID_{Cor})
Symmetry C_{6v}
Energy MP2 = −3492.870486 au
Standard Orientation

6	0	0.710813	1.231164	-0.680842
6	0	-0.710813	1.231164	-0.680842
6	0	-1.421626	0.000000	-0.680842
6	0	-0.710813	-1.231164	-0.680842
6	0	0.710813	-1.231164	-0.680842
6	0	1.421626	0.000000	-0.680842
6	0	1.422504	2.463850	-0.661650
6	0	0.688103	3.677493	-0.650078
6	0	-0.688103	3.677493	-0.650078
1	0	1.233350	4.618216	-0.636583
1	0	-1.233350	4.618216	-0.636583
6	0	-1.422504	2.463850	-0.661650
6	0	-2.840751	2.434661	-0.650078
6	0	-3.528854	1.242832	-0.650078
1	0	-3.382817	3.377220	-0.636583
1	0	-4.616167	1.240996	-0.636583
6	0	-2.845009	0.000000	-0.661650
6	0	-3.528854	-1.242832	-0.650078
6	0	-2.840751	-2.434661	-0.650078
1	0	-4.616167	-1.240996	-0.636583
1	0	-3.382817	-3.377220	-0.636583
6	0	-1.422504	-2.463850	-0.661650
6	0	-0.688103	-3.677493	-0.650078
6	0	0.688103	-3.677493	-0.650078
1	0	-1.233350	-4.618216	-0.636583
1	0	1.233350	-4.618216	-0.636583
6	0	1.422504	-2.463850	-0.661650
6	0	2.840751	-2.434661	-0.650078
6	0	3.528854	-1.242832	-0.650078
1	0	3.382817	-3.377220	-0.636583
1	0	4.616167	-1.240996	-0.636583
6	0	2.845009	0.000000	-0.661650
6	0	3.528854	1.242832	-0.650078
6	0	2.840751	2.434661	-0.650078
1	0	4.616167	1.240996	-0.636583
1	0	3.382817	3.377220	-0.636583
1	0	0.000000	0.000000	1.474614
35	0	0.000000	0.000000	2.894277

Adduct $C_{24}H_{12}$−HI (type: ID_{Cor})
Symmetry C_{6v}
Energy MP2 = −7838.220220 au

Standard Orientation

6	0	0.710716	1.230997	-0.975330
6	0	-0.710716	1.230997	-0.975330
6	0	-1.421433	0.000000	-0.975330
6	0	-0.710716	-1.230997	-0.975330
6	0	0.710716	-1.230997	-0.975330
6	0	1.421433	0.000000	-0.975330
6	0	1.422362	2.463604	-0.953260
6	0	0.688114	3.677330	-0.939349
6	0	-0.688114	3.677330	-0.939349
1	0	1.233428	4.617997	-0.923836
1	0	-1.233428	4.617997	-0.923836
6	0	-1.422362	2.463604	-0.953260
6	0	-2.840604	2.434589	-0.939349
6	0	-3.528718	1.242741	-0.939349
1	0	-3.382589	3.377178	-0.923836
1	0	-4.616017	1.240819	-0.923836
6	0	-2.844725	0.000000	-0.953260
6	0	-3.528718	-1.242741	-0.939349
6	0	-2.840604	-2.434589	-0.939349
1	0	-4.616017	-1.240819	-0.923836
1	0	-3.382589	-3.377178	-0.923836
6	0	-1.422362	-2.463604	-0.953260
6	0	-0.688114	-3.677330	-0.939349
6	0	0.688114	-3.677330	-0.939349
1	0	-1.233428	-4.617997	-0.923836
1	0	1.233428	-4.617997	-0.923836
6	0	1.422362	-2.463604	-0.953260
6	0	2.840604	-2.434589	-0.939349
6	0	3.528718	-1.242741	-0.939349
1	0	3.382589	-3.377178	-0.923836
1	0	4.616017	-1.240819	-0.923836
6	0	2.844725	0.000000	-0.953260
6	0	3.528718	1.242741	-0.939349
6	0	2.840604	2.434589	-0.939349
1	0	4.616017	1.240819	-0.923836
1	0	3.382589	3.377178	-0.923836
1	0	0.000000	0.000000	1.159503
53	0	0.000000	0.000000	2.773375

Adduct $C_{24}H_{12}$−F_2 (type: ID_{Cor})
Symmetry C_{6v}
Energy MP2 = −1118.822131 au
Standard Orientation

6	0	0.710471	1.230571	-0.361727
6	0	-0.710471	1.230571	-0.361727
6	0	-1.420941	0.000000	-0.361727
6	0	-0.710471	-1.230571	-0.361727
6	0	0.710471	-1.230571	-0.361727
6	0	1.420941	0.000000	-0.361727
6	0	1.422048	2.463059	-0.354476
6	0	0.688019	3.677338	-0.350118

6	0	-0.688019	3.677338	-0.350118
1	0	1.233515	4.618103	-0.345304
1	0	-1.233515	4.618103	-0.345304
6	0	-1.422048	2.463059	-0.354476
6	0	-2.840659	2.434511	-0.350118
6	0	-3.528678	1.242827	-0.350118
1	0	-3.382637	3.377307	-0.345304
1	0	-4.616152	1.240796	-0.345304
6	0	-2.844096	0.000000	-0.354476
6	0	-3.528678	-1.242827	-0.350118
6	0	-2.840659	-2.434511	-0.350118
1	0	-4.616152	-1.240796	-0.345304
1	0	-3.382637	-3.377307	-0.345304
6	0	-1.422048	-2.463059	-0.354476
6	0	-0.688019	-3.677338	-0.350118
6	0	0.688019	-3.677338	-0.350118
1	0	-1.233515	-4.618103	-0.345304
1	0	1.233515	-4.618103	-0.345304
6	0	1.422048	-2.463059	-0.354476
6	0	2.840659	-2.434511	-0.350118
6	0	3.528678	-1.242827	-0.350118
1	0	3.382637	-3.377307	-0.345304
1	0	4.616152	-1.240796	-0.345304
6	0	2.844096	0.000000	-0.354476
6	0	3.528678	1.242827	-0.350118
6	0	2.840659	2.434511	-0.350118
1	0	4.616152	1.240796	-0.345304
1	0	3.382637	3.377307	-0.345304
9	0	0.000000	0.000000	2.361706
9	0	0.000000	0.000000	3.764450

Adduct $C_{24}H_{12}{-}Cl_2$ (type: ID_{Cor})
Symmetry C_{6v}
Energy MP2 = −1838.920129 au
Standard Orientation

6	0	0.710551	1.230711	-0.722943
6	0	-0.710551	1.230711	-0.722943
6	0	-1.421102	0.000000	-0.722943
6	0	-0.710551	-1.230711	-0.722943
6	0	0.710551	-1.230711	-0.722943
6	0	1.421102	0.000000	-0.722943
6	0	1.422022	2.463014	-0.699651
6	0	0.688053	3.677039	-0.684643
6	0	-0.688053	3.677039	-0.684643
1	0	1.233554	4.617662	-0.668431
1	0	-1.233554	4.617662	-0.668431
6	0	-1.422022	2.463014	-0.699651
6	0	-2.840383	2.434391	-0.684643
6	0	-3.528436	1.242648	-0.684643
1	0	-3.382236	3.377120	-0.668431
1	0	-4.615790	1.240542	-0.668431
6	0	-2.844044	0.000000	-0.699651

6	0	-3.528436	-1.242648	-0.684643
6	0	-2.840383	-2.434391	-0.684643
1	0	-4.615790	-1.240542	-0.668431
1	0	-3.382236	-3.377120	-0.668431
6	0	-1.422022	-2.463014	-0.699651
6	0	-0.688053	-3.677039	-0.684643
6	0	0.688053	-3.677039	-0.684643
1	0	-1.233554	-4.617662	-0.668431
1	0	1.233554	-4.617662	-0.668431
6	0	1.422022	-2.463014	-0.699651
6	0	2.840383	-2.434391	-0.684643
6	0	3.528436	-1.242648	-0.684643
1	0	3.382236	-3.377120	-0.668431
1	0	4.615790	-1.240542	-0.668431
6	0	2.844044	0.000000	-0.699651
6	0	3.528436	1.242648	-0.684643
6	0	2.840383	2.434391	-0.684643
1	0	4.615790	1.240542	-0.668431
1	0	3.382236	3.377120	-0.668431
17	0	0.000000	0.000000	2.196361
17	0	0.000000	0.000000	4.187687

Adduct $C_{24}H_{12}$−Br_2 (type: ID_{Cor})
Symmetry C_{6v}
Energy MP2 = −6064.982882 au
Standard Orientation

6	0	0.710668	1.230913	-1.306422
6	0	-0.710668	1.230913	-1.306422
6	0	-1.421336	0.000000	-1.306422
6	0	-0.710668	-1.230913	-1.306422
6	0	0.710668	-1.230913	-1.306422
6	0	1.421336	0.000000	-1.306422
6	0	1.422115	2.463175	-1.279876
6	0	0.688079	3.677078	-1.262392
6	0	-0.688079	3.677078	-1.262392
1	0	1.233542	4.617670	-1.243600
1	0	-1.233542	4.617670	-1.243600
6	0	-1.422115	2.463175	-1.279876
6	0	-2.840403	2.434433	-1.262392
6	0	-3.528482	1.242645	-1.262392
1	0	-3.382249	3.377114	-1.243600
1	0	-4.615791	1.240556	-1.243600
6	0	-2.844230	0.000000	-1.279876
6	0	-3.528482	-1.242645	-1.262392
6	0	-2.840403	-2.434433	-1.262392
1	0	-4.615791	-1.240556	-1.243600
1	0	-3.382249	-3.377114	-1.243600
6	0	-1.422115	-2.463175	-1.279876
6	0	-0.688079	-3.677078	-1.262392
6	0	0.688079	-3.677078	-1.262392
1	0	-1.233542	-4.617670	-1.243600
1	0	1.233542	-4.617670	-1.243600

6	0	1.422115	-2.463175	-1.279876
6	0	2.840403	-2.434433	-1.262392
6	0	3.528482	-1.242645	-1.262392
1	0	3.382249	-3.377114	-1.243600
1	0	4.615791	-1.240556	-1.243600
6	0	2.844230	0.000000	-1.279876
6	0	3.528482	1.242645	-1.262392
6	0	2.840403	2.434433	-1.262392
1	0	4.615791	1.240556	-1.243600
1	0	3.382249	3.377114	-1.243600
35	0	0.000000	0.000000	1.702674
35	0	0.000000	0.000000	3.980817

Adduct $C_{24}H_{12}$–I_2 (type: ID_{Cor})
Symmetry C_{6v}
Energy MP2 = −14755.721007 au
Standard Orientation

6	0	0.710707	1.230981	-1.825873
6	0	-0.710707	1.230981	-1.825873
6	0	-1.421414	0.000000	-1.825873
6	0	-0.710707	-1.230981	-1.825873
6	0	0.710707	-1.230981	-1.825873
6	0	1.421414	0.000000	-1.825873
6	0	1.422079	2.463113	-1.794779
6	0	0.688098	3.676879	-1.773425
6	0	-0.688098	3.676879	-1.773425
1	0	1.233569	4.617365	-1.750967
1	0	-1.233569	4.617365	-1.750967
6	0	-1.422079	2.463113	-1.794779
6	0	-2.840222	2.434350	-1.773425
6	0	-3.528320	1.242529	-1.773425
1	0	-3.381971	3.376985	-1.750967
1	0	-4.615540	1.240380	-1.750967
6	0	-2.844158	0.000000	-1.794779
6	0	-3.528320	-1.242529	-1.773425
6	0	-2.840222	-2.434350	-1.773425
1	0	-4.615540	-1.240380	-1.750967
1	0	-3.381971	-3.376985	-1.750967
6	0	-1.422079	-2.463113	-1.794779
6	0	-0.688098	-3.676879	-1.773425
6	0	0.688098	-3.676879	-1.773425
1	0	-1.233569	-4.617365	-1.750967
1	0	1.233569	-4.617365	-1.750967
6	0	1.422079	-2.463113	-1.794779
6	0	2.840222	-2.434350	-1.773425
6	0	3.528320	-1.242529	-1.773425
1	0	3.381971	-3.376985	-1.750967
1	0	4.615540	-1.240380	-1.750967
6	0	2.844158	0.000000	-1.794779
6	0	3.528320	1.242529	-1.773425
6	0	2.840222	2.434350	-1.773425
1	0	4.615540	1.240380	-1.750967

1	0	3.381971	3.376985	-1.750967
53	0	0.000000	0.000000	1.299359
53	0	0.000000	0.000000	3.965578

Adduct $C_{24}H_{12}$−ClF (type: ID_{Cor})
Symmetry C_{6v}
Energy MP2 = −1478.897820 au
Standard Orientation

6	0	0.710708	1.230983	-0.523200
6	0	-0.710708	1.230983	-0.523200
6	0	-1.421417	0.000000	-0.523200
6	0	-0.710708	-1.230983	-0.523200
6	0	0.710708	-1.230983	-0.523200
6	0	1.421417	0.000000	-0.523200
6	0	1.422232	2.463378	-0.502886
6	0	0.688061	3.677287	-0.490015
6	0	-0.688061	3.677287	-0.490015
1	0	1.233425	4.617978	-0.475811
1	0	-1.233425	4.617978	-0.475811
6	0	-1.422232	2.463378	-0.502886
6	0	-2.840593	2.434522	-0.490015
6	0	-3.528654	1.242765	-0.490015
1	0	-3.382574	3.377166	-0.475811
1	0	-4.615999	1.240812	-0.475811
6	0	-2.844464	0.000000	-0.502886
6	0	-3.528654	-1.242765	-0.490015
6	0	-2.840593	-2.434522	-0.490015
1	0	-4.615999	-1.240812	-0.475811
1	0	-3.382574	-3.377166	-0.475811
6	0	-1.422232	-2.463378	-0.502886
6	0	-0.688061	-3.677287	-0.490015
6	0	0.688061	-3.677287	-0.490015
1	0	-1.233425	-4.617978	-0.475811
1	0	1.233425	-4.617978	-0.475811
6	0	1.422232	-2.463378	-0.502886
6	0	2.840593	-2.434522	-0.490015
6	0	3.528654	-1.242765	-0.490015
1	0	3.382574	-3.377166	-0.475811
1	0	4.615999	-1.240812	-0.475811
6	0	2.844464	0.000000	-0.502886
6	0	3.528654	1.242765	-0.490015
6	0	2.840593	2.434522	-0.490015
1	0	4.615999	1.240812	-0.475811
1	0	3.382574	3.377166	-0.475811
17	0	0.000000	0.000000	2.428378
9	0	0.000000	0.000000	4.071945

Adduct $C_{24}H_{12}$−BrF (type: ID_{Cor})
Symmetry C_{6v}
Energy MP2 = −3591.937507 au
Standard Orientation

6	0	0.710856	1.231239	-0.763966
6	0	-0.710856	1.231239	-0.763966
6	0	-1.421712	0.000000	-0.763966
6	0	-0.710856	-1.231239	-0.763966
6	0	0.710856	-1.231239	-0.763966
6	0	1.421712	0.000000	-0.763966
6	0	1.422323	2.463535	-0.739574
6	0	0.688075	3.677283	-0.723615
6	0	-0.688075	3.677283	-0.723615
1	0	1.233386	4.617922	-0.706167
1	0	-1.233386	4.617922	-0.706167
6	0	-1.422323	2.463535	-0.739574
6	0	-2.840583	2.434532	-0.723615
6	0	-3.528658	1.242751	-0.723615
1	0	-3.382545	3.377105	-0.706167
1	0	-4.615931	1.240817	-0.706167
6	0	-2.844645	0.000000	-0.739574
6	0	-3.528658	-1.242751	-0.723615
6	0	-2.840583	-2.434532	-0.723615
1	0	-4.615931	-1.240817	-0.706167
1	0	-3.382545	-3.377105	-0.706167
6	0	-1.422323	-2.463535	-0.739574
6	0	-0.688075	-3.677283	-0.723615
6	0	0.688075	-3.677283	-0.723615
1	0	-1.233386	-4.617922	-0.706167
1	0	1.233386	-4.617922	-0.706167
6	0	1.422323	-2.463535	-0.739574
6	0	2.840583	-2.434532	-0.723615
6	0	3.528658	-1.242751	-0.723615
1	0	3.382545	-3.377105	-0.706167
1	0	4.615931	-1.240817	-0.706167
6	0	2.844645	0.000000	-0.739574
6	0	3.528658	1.242751	-0.723615
6	0	2.840583	2.434532	-0.723615
1	0	4.615931	1.240817	-0.706167
1	0	3.382545	3.377105	-0.706167
35	0	0.000000	0.000000	2.243255
9	0	0.000000	0.000000	4.020871

Adduct $C_{24}H_{12}$−IF (type: ID_{Cor})
Symmetry C_{6v}
Energy MP2 = −7937.325535 au
Standard Orientation

6	0	-1.231424	0.710963	-0.983954
6	0	-1.231424	-0.710963	-0.983954
6	0	0.000000	-1.421926	-0.983954
6	0	1.231424	-0.710963	-0.983954
6	0	1.231424	0.710963	-0.983954
6	0	0.000000	1.421926	-0.983954
6	0	-2.463518	1.422313	-0.954384
6	0	-3.677030	0.688081	-0.933838
6	0	-3.677030	-0.688081	-0.933838

1	0	-4.617551	1.233367	-0.911877
1	0	-4.617551	-1.233367	-0.911877
6	0	-2.463518	-1.422313	-0.954384
6	0	-2.434410	-2.840360	-0.933838
6	0	-1.242619	-3.528442	-0.933838
1	0	-3.376903	-3.382233	-0.911877
1	0	-1.240648	-4.615600	-0.911877
6	0	0.000000	-2.844625	-0.954384
6	0	1.242619	-3.528442	-0.933838
6	0	2.434410	-2.840360	-0.933838
1	0	1.240648	-4.615600	-0.911877
1	0	3.376903	-3.382233	-0.911877
6	0	2.463518	-1.422313	-0.954384
6	0	3.677030	-0.688081	-0.933838
6	0	3.677030	0.688081	-0.933838
1	0	4.617551	-1.233367	-0.911877
1	0	4.617551	1.233367	-0.911877
6	0	2.463518	1.422313	-0.954384
6	0	2.434410	2.840360	-0.933838
6	0	1.242619	3.528442	-0.933838
1	0	3.376903	3.382233	-0.911877
1	0	1.240648	4.615600	-0.911877
6	0	0.000000	2.844625	-0.954384
6	0	-1.242619	3.528442	-0.933838
6	0	-2.434410	2.840360	-0.933838
1	0	-1.240648	4.615600	-0.911877
1	0	-3.376903	3.382233	-0.911877
53	0	0.000000	0.000000	2.105807
9	0	0.000000	0.000000	4.039033

Adduct C_6H_6
Symmetry D_{6h}
Energy MP2 = −231.622427 au
Standard Orientation

6	0	0.000000	1.395330	0.000000
6	0	1.208391	0.697665	0.000000
6	0	1.208391	-0.697665	0.000000
6	0	0.000000	-1.395330	0.000000
6	0	-1.208391	-0.697665	0.000000
6	0	-1.208391	0.697665	0.000000
1	0	0.000000	2.480666	0.000000
1	0	2.148320	1.240333	0.000000
1	0	2.148320	-1.240333	0.000000
1	0	0.000000	-2.480666	0.000000
1	0	-2.148320	-1.240333	0.000000
1	0	-2.148320	1.240333	0.000000

Adduct C_6H_6−HF (type: ID_{Bzn})
Symmetry C_{6v}
Energy MP2 = −331.958210 au
Standard Orientation

6	0	0.000000	1.396596	-0.588618
6	0	1.209488	0.698298	-0.588618
6	0	-1.209488	0.698298	-0.588618
6	0	1.209488	-0.698298	-0.588618
6	0	-1.209488	-0.698298	-0.588618
6	0	0.000000	-1.396596	-0.588618
1	0	0.000000	2.481468	-0.578677
1	0	2.149014	1.240734	-0.578677
1	0	-2.149014	1.240734	-0.578677
1	0	2.149014	-1.240734	-0.578677
1	0	-2.149014	-1.240734	-0.578677
1	0	0.000000	-2.481468	-0.578677
1	0	0.000000	0.000000	1.633724
9	0	0.000000	0.000000	2.558730

Adduct C_6H_6−HCl (type: ID_{Bzn})
Symmetry C_{6v}
Energy MP2 = −691.928976 au
Standard Orientation

6	0	0.000000	1.396481	-1.036932
6	0	1.209385	0.698241	-1.036926
6	0	-1.209385	0.698241	-1.036926
6	0	1.209385	-0.698241	-1.036926
6	0	-1.209385	-0.698241	-1.036926
6	0	0.000000	-1.396481	-1.036932
1	0	0.000000	2.481523	-1.028256
1	0	2.149061	1.240757	-1.028190
1	0	-2.149061	1.240757	-1.028190
1	0	2.149061	-1.240757	-1.028190
1	0	-2.149061	-1.240757	-1.028190
1	0	0.000000	-2.481523	-1.028256
1	0	0.000000	0.000000	1.205240
17	0	0.000000	0.000000	2.487850

Adduct C_6H_6−HBr (type: ID_{Bzn})
Symmetry C_{6v}
Energy MP2 = −2804.942234 au
Standard Orientation

6	0	0.000000	1.396439	-1.681325
6	0	1.209352	0.698219	-1.681325
6	0	-1.209352	0.698220	-1.681325
6	0	1.209352	-0.698220	-1.681325
6	0	-1.209352	-0.698220	-1.681325
6	0	0.000000	-1.396439	-1.681325
1	0	0.000000	2.481546	-1.673814
1	0	2.149082	1.240773	-1.673814
1	0	-2.149082	1.240773	-1.673814
1	0	2.149082	-1.240773	-1.673814
1	0	-2.149082	-1.240773	-1.673814
1	0	0.000000	-2.481546	-1.673814
1	0	0.000000	0.000000	0.579190

35	0	0.000000	0.000000	1.999754

Adduct C_6H_6−HI (type: ID_{Bzn})
Symmetry C_{6v}
Energy MP2 = −7150.290414 au
Standard Orientation

6	0	0.000000	1.396244	-2.171691
6	0	1.209183	0.698122	-2.171691
6	0	-1.209183	0.698122	-2.171691
6	0	1.209183	-0.698122	-2.171691
6	0	-1.209183	-0.698122	-2.171691
6	0	0.000000	-1.396244	-2.171691
1	0	0.000000	2.481421	-2.165441
1	0	2.148974	1.240711	-2.165441
1	0	-2.148974	1.240711	-2.165441
1	0	2.148974	-1.240711	-2.165441
1	0	-2.148974	-1.240711	-2.165441
1	0	0.000000	-2.481421	-2.165441
1	0	0.000000	0.000000	0.103172
53	0	0.000000	0.000000	1.718308

Adduct C_6H_6−F_2 (type: ID_{Bzn})
Symmetry C_{6v}
Energy MP2 = −430.893808 au
Standard Orientation

6	0	0.000000	1.395506	-1.081674
6	0	1.208544	0.697753	-1.081674
6	0	-1.208544	0.697753	-1.081674
6	0	1.208544	-0.697753	-1.081674
6	0	-1.208544	-0.697753	-1.081674
6	0	0.000000	-1.395506	-1.081674
1	0	0.000000	2.480841	-1.079006
1	0	2.148471	1.240420	-1.079006
1	0	-2.148471	1.240420	-1.079006
1	0	2.148471	-1.240421	-1.079006
1	0	-2.148471	-1.240420	-1.079006
1	0	0.000000	-2.480841	-1.079006
9	0	0.000000	0.000000	1.822378
9	0	0.000000	0.000000	3.223656

Adduct C_6H_6−Cl_2 (type: ID_{Bzn})
Symmetry C_{6v}
Energy MP2 = −1150.988862 au
Standard Orientation

6	0	0.000000	1.395972	-1.835061
6	0	1.208947	0.697986	-1.835061
6	0	-1.208947	0.697986	-1.835061
6	0	1.208947	-0.697986	-1.835061
6	0	-1.208947	-0.697986	-1.835061
6	0	0.000000	-1.395972	-1.835061
1	0	0.000000	2.481280	-1.829306

1	0	2.148852	1.240640	-1.829306
1	0	-2.148852	1.240640	-1.829306
1	0	2.148852	-1.240640	-1.829306
1	0	-2.148852	-1.240640	-1.829306
1	0	0.000000	-2.481280	-1.829306
17	0	0.000000	0.000000	1.270776
17	0	0.000000	0.000000	3.260874

Adduct C_6H_6−Br_2 (type: ID_{Bzn})
Symmetry C_{6v}
Energy MP2 = −5377.050710 au
Standard Orientation

6	0	0.000000	1.396194	-2.727293
6	0	1.209139	0.698097	-2.727293
6	0	-1.209139	0.698097	-2.727293
6	0	1.209139	-0.698097	-2.727293
6	0	-1.209139	-0.698097	-2.727293
6	0	0.000000	-1.396194	-2.727293
1	0	0.000000	2.481520	-2.722577
1	0	2.149059	1.240760	-2.722577
1	0	-2.149059	1.240760	-2.722577
1	0	2.149059	-1.240760	-2.722577
1	0	-2.149059	-1.240760	-2.722577
1	0	0.000000	-2.481520	-2.722577
35	0	0.000000	0.000000	0.498281
35	0	0.000000	0.000000	2.773662

Adduct C_6H_6−I_2 (type: ID_{Bzn})
Symmetry C_{6v}
Energy MP2 = −14067.786555 au
Standard Orientation

6	0	0.000000	1.396238	-3.370057
6	0	1.209178	0.698119	-3.370057
6	0	-1.209178	0.698119	-3.370057
6	0	1.209178	-0.698119	-3.370057
6	0	-1.209178	-0.698119	-3.370057
6	0	0.000000	-1.396238	-3.370057
1	0	0.000000	2.481534	-3.365425
1	0	2.149071	1.240767	-3.365425
1	0	-2.149071	1.240767	-3.365425
1	0	2.149071	-1.240767	-3.365425
1	0	-2.149071	-1.240767	-3.365425
1	0	0.000000	-2.481534	-3.365425
53	0	0.000000	0.000000	0.003905
53	0	0.000000	0.000000	2.666182

Adduct C_6H_6−ClF (type: ID_{Bzn})
Symmetry C_{6v}
Energy MP2 = −790.968094 au
Standard Orientation

6	0	0.000000	1.396172	-1.411154

6	0	1.209120	0.698086	-1.411154
6	0	-1.209120	0.698086	-1.411154
6	0	1.209120	-0.698086	-1.411154
6	0	-1.209120	-0.698086	-1.411154
6	0	0.000000	-1.396172	-1.411154
1	0	0.000000	2.481406	-1.405287
1	0	2.148961	1.240703	-1.405287
1	0	-2.148961	1.240703	-1.405287
1	0	2.148961	-1.240703	-1.405287
1	0	-2.148961	-1.240703	-1.405287
1	0	0.000000	-2.481406	-1.405287
17	0	0.000000	0.000000	1.709828
9	0	0.000000	0.000000	3.351799

Adduct C_6H_6−BrF (type: ID_{Bzn})
Symmetry C_{6v}
Energy MP2 = −2904.006822 au
Standard Orientation

6	0	0.000000	1.396442	-1.825225
6	0	1.209354	0.698221	-1.825225
6	0	-1.209354	0.698221	-1.825225
6	0	1.209354	-0.698221	-1.825225
6	0	-1.209354	-0.698221	-1.825225
6	0	0.000000	-1.396442	-1.825225
1	0	0.000000	2.481654	-1.819727
1	0	2.149175	1.240827	-1.819727
1	0	-2.149175	1.240827	-1.819727
1	0	2.149175	-1.240827	-1.819727
1	0	-2.149175	-1.240827	-1.819727
1	0	0.000000	-2.481654	-1.819727
35	0	0.000000	0.000000	1.378483
9	0	0.000000	0.000000	3.153286

Adduct C_6H_6−IF (type: ID_{Bzn})
Symmetry C_{6v}
Energy MP2 = −7249.392607 au
Standard Orientation

6	0	0.000000	1.396577	-2.145721
6	0	1.209471	0.698288	-2.145721
6	0	-1.209471	0.698288	-2.145721
6	0	1.209471	-0.698288	-2.145721
6	0	-1.209471	-0.698288	-2.145721
6	0	0.000000	-1.396577	-2.145721
1	0	0.000000	2.481720	-2.138921
1	0	2.149233	1.240860	-2.138921
1	0	-2.149233	1.240860	-2.138921
1	0	2.149233	-1.240860	-2.138921
1	0	-2.149233	-1.240860	-2.138921
1	0	0.000000	-2.481720	-2.138921
53	0	0.000000	0.000000	1.172720
9	0	0.000000	0.000000	3.102812
